# Updated systematic review and meta-analysis: taking the next step in physical activity behavioral interventions for post-treatment breast cancer survivors

**DOI:** 10.1007/s10549-025-07892-3

**Published:** 2026-01-10

**Authors:** Brianna N. Leitzelar, Alana R. Willis, Sarah N. Price, Janet A. Tooze, Helena M. VonVille, Rachel Lintz, Shirley M. Bluethmann

**Affiliations:** 1 School of Kinesiology, University of Minnesota-Twin Cities, Minneapolis, MN USA; 2 Department of Social Sciences and Health Policy, Wake Forest University School of Medicine, Winston-Salem, NC USA; 3 Department of Biostatistics and Data Science, Wake Forest University School of Medicine, Winston-Salem, NC USA; 4 Health Sciences Library System, University of Pittsburgh, Pittsburgh, PA USA; 5 Department of Public Health Sciences, Penn State College of Medicine, Hershey, PA USA

**Keywords:** Breast cancer, Survivorship, Physical activity, Behavior change, Cancer rehabilitation

## Abstract

**Purpose:**

To provide an updated review of the literature on physical activity (PA) intervention studies, their characteristics, and their effect size estimates for PA behavior change among early post-treatment breast cancer survivors (BCS).

**Methods:**

Eligible studies were published between 2014–2025 in English, were quasi- or randomized controlled trials, studied BCS ≤ 5 years post-treatment, tested a PA intervention, and assessed PA behavior. We searched PubMed, APA PsycINFO, Embase, and CINAHL (latest search October 2025; CINAHL June 2020). Extracted data included study, participant, intervention, and outcome descriptors. The ROB 2 assessed risk of bias. A random effects model on post-intervention Cohen’s d standardized mean differences (SMD) values meta-analysis was performed.

**Results:**

Twenty-two RCTs with a total sample size of 2,390 (mean = 109, range = 26–692) were included. All included BCS were female, were on average 57 years old, and predominantly (> 60%) non-Hispanic White. Most study populations were mixed in terms of cancer stage and treatment type. Intervention duration ranged from 6–104 weeks. All studies except one were partially or fully home-based. All behavioral counseling interventions were theory-based. The overall SMD was d = 0.36 (95% confidence interval: 0.22, 0.50) in favor of the intervention. Two studies had some concerns for risk of bias; all others were rated as low.

**Conclusion:**

The present updated review found a small-to-moderate positive effect of PA interventions on PA behavior change among early post-treatment BCS. We note some shifts in the participant samples and study design since the originally published review. Practical implications for improving the reporting of future research include following established reporting guidelines to enhance reporting transparency, which would allow for more precise quantification of specific intervention effects and deeper contextual understanding of this body of work.

**Supplementary Information:**

The online version contains supplementary material available at 10.1007/s10549-025-07892-3.

## Introduction

Steady increases in breast cancer incidence, paired with high 5-year survival rates (95%), highlights a need to understand breast cancer survivorship, or the period of time following primary active treatment. The completion of primary breast cancer treatment is a life-changing event for many survivors, representing both an opportunity and a challenge. Challenges may span physical effects such as managing long-term sequalae of cancer treatment, and psychosocial, such as reestablishing routines and relationships or coping with psychological distress [1, 2]. On the other hand, beginning a new post-treatment stage of breast cancer survivorship offers an opportunity to engage in health promotion behaviors [3], though adopting, returning to, or maintaining a physically active lifestyle following treatment is challenging [4, 5]. Physical activity (PA) is often a safe and impactful component of survivorship planning guidelines for cancer survivors [6–8]. Evidence-based recommendations for physical activity include 150–300 min of moderate-to-vigorous intensity aerobic PA and 2 days per week of muscle strengthening activity [7, 8]. Although muscle strengthening activity is an important aspect of improving strength and health post-treatment [8, 9], the evidence to-date overwhelmingly lies in understanding aerobic physical activity and the present review focuses mainly on aerobic PA. Post-treatment PA is associated with reduced risk of cancer recurrence, mitigated cancer treatment side effects, and enhanced quality of life [8, 10, 11]. Despite these benefits, only 40–50% of BCS meet aerobic PA recommendations [12, 13], reflecting a need for interventions to support PA behavior change. In 2015, Bluethmann and associates (2015) published a systematic review and meta-analysis to rigorously assess the effectiveness of interventions for PA behavior change (i.e., behavioral PA interventions) among recent, post-treatment BCS which demonstrated that published interventions had a moderate effect for short-term behavior change [14].

Given it has been 10 years since the original review was published and there has been a noticeable uptick in published papers related to physical activity and breast cancer around 2015 [15], an updated review of the literature is warranted. The purpose of the present systematic review and meta-analysis is to update the literature from the original review published in 2015 [14]. The aims are to: (1) describe the characteristics of PA behavior interventions for BCS, including targeted populations, intervention features, and use of behavior theory and to (2) determine effect size estimates for behavior change from these PA interventions. For the purposes of this paper, intervention is defined as a strategy or set of strategies, often derived from behavior change theories, to influence health behaviors, such as PA [16]. We chose the term PA, rather than exercise, to include interventions that target moderate-to-vigorous intensity physical activity (MVPA) but may not require access to exercise facilities or equipment.

## Methods

Reporting of this review has been done in accordance with the Preferred Reporting of Systematic Reviews and Meta-Analysis (PRISMA) guidelines [17]. The protocol was pre-registered at PROSPERO (registration number: CRD42020192951).

### Eligibility criteria

Eligibility criteria were identical to the original systematic review [14]; the only difference was publication dates. Eligible studies: (1) studied BCS ≤ 5 years post-treatment or could provide data specific to BCS ≤ 5 years post-treatment; (2) included a PA intervention; (3) assessed PA behavior change; (4) utilized a randomized controlled trial (RCT) or quasi-experimental trial with a comparison group design; and (5) been published in English in a research journal from 2013 forward. Studies included in the original SR were excluded.

### Information sources & search strategy

A health sciences librarian with systematic review experience (HV) conducted all searches of: PubMed (National Library of Medicine; last search October 2025), APA PsycINFO (Ovid; last search: October 2025), Embase (Elsevier; last search: October 2025), and CINAHL (EBSCO; last search June 2020). The initial search in CINAHL in June 2020 did not return unique reports, thus this database was not included in subsequent searches. Concepts used to develop the searches were: breast cancer, survivorship, trials/evaluation studies. Data related to the searches as well as the full search strategies can be found in Online Resource 1.

### Study selection

Studies were selected in a two-stage process by two independent reviewers (BNL, SM, SMB) using a series of Excel Workbooks developed for systematic reviews [18]. The reviewers independently screened citations and abstracts for eligibility, excluding those that did not meet the criteria. Conflicting classifications were resolved through verbal discussion. Working independently again, reviewers then assessed the full text articles of the remaining studies to determine inclusion. The reviewers met to resolve any discrepancies and finalized the list of studies meeting eligibility criteria for inclusion.

### Data collection process

Data abstraction techniques followed the same procedure as the original review [14]. Using an Excel workbook, two coders (BNL, SNP, SM) independently extracted data from the included studies. All discrepancies were checked against the original manuscript and resolved through discussion with the study team. Four studies required contact with the corresponding author to obtain data specific to BCS [19], for BCS within 5 years post-treatment [20, 21], or for intervention-level descriptive data [22].

### Data items

PA behavior immediately following the intervention was the main outcome of interest. Relevant outcomes included minutes per week, Metabolic Equivalent Task hours per week, times per week, steps per day, and categorical variables such as proportion of participants meeting PA guidelines or within PA activity level groups (i.e., insufficiently active, moderately active, active). In cases when multiple methods (e.g., self-report, device-monitored) were utilized to assess PA, we selected self-reported PA outcomes in alignment with the data utilized to develop national PA guidelines [23]. When multiple PA outcomes were available, the outcome most closely aligned with MVPA was included. For example, if a study reported total walking minutes, total lifestyle activity, and MVPA, MVPA was the outcome selected for the meta-analysis.

Other data items included participant, study, and intervention characteristics (Online Resource 2). In brief, participant characteristics included: age, age-related eligibility criteria, race and/or ethnicity, years since diagnosis or treatment, stage at diagnosis, treatment type, geographic origin, income level, and baseline PA levels. Study characteristics included study type, eligibility criteria, sample size, number of treatment groups, and any baseline equivalence testing between groups. Intervention characteristics included intervention features, setting, duration (number of sessions, minutes per session), and frequency; use of behavioral theory, and if authors anchored PA goals to national PA guidelines. Consistent with the original review [14], we categorized interventions based on the level of supervision required by the intervention. Categories were low (e.g., little or no PA oversight), medium (i.e., behavioral counseling present but no structured PA), and high (i.e., multi-component intervention with structured participant interactions and/or supervised PA).

### Study risk of bias assessment

Authors RL and BL conducted the risk of bias assessment using the Cochrane Collective ROB 2 tool [24, 25], assessing 5 intervention features separately (randomization process, deviations from intended interventions, missing outcome data, measurement of the outcome, and selection of the reported result). Each study was then assigned an overall bias rating (low, some concerns, and high) depending on the number of features judged low, some concerns, and high following the published guidance [24].

### Synthesis methods

Studies were included in the synthesis if they reported outcomes for both a treatment and a control group. Studies with compatible control groups and outcome measures relevant to the planned synthesis were included in the quantitative analysis. To enhance precision of the effect size, outcome data from both the original and updated searches were also included. Only studies identified in the updated search were included in the qualitative synthesis. The qualitative synthesis for the original search was previously published [14].

Data was compiled by BNL in Microsoft Excel and provided to the biostatistical team for analysis. The intervention effect was calculated by comparing post-intervention data between treatment and control groups. For most studies, Cohen’s d was calculated using the post-intervention means and pooled standard deviations (SDs) for the treatment and control groups using the standard formula [26, 27]. For studies that reported only change score from baseline, we estimated post-intervention means using the baseline data and assuming the same standard deviation as at baseline in accordance with Wilson (2017) [27]. Cohen’s d for studies reporting categorical outcomes were calculated using the 2× 2 frequency table method [27]. Cohen’s d was selected as the standardized effect size to ensure comparability across studies.

A random effects meta-analysis was conducted to account for between-study variability in effect sizes. Heterogeneity was assessed using the I^2^ statistic and Cochran’s Q test. To evaluate the statistical significance of the pooled effect estimate, a Z-test was performed. Statistical significance was determined at a two-sided alpha level of 0.05. Forest plots were generated to visually display individual study results and the overall pooled estimates. All analyses were performed in RStudio version 4.2.1 using metafor, effect size, and meta packages.

To further explore potential sources of heterogeneity among study results, subgroup analyses were conducted using random effects models. Between-study heterogeneity within subgroups was assessed using the I^2^ statistic and Cochran’s Q test. To evaluate whether effect sizes differed significantly between subgroups, a chi-squared (Q) test for heterogeneity between groups was performed. Subgroups were defined based on the level of supervision (low, medium, high), duration of the intervention (≤ 12 weeks, > 12 weeks), average participant age weighted by the study sample size (< 60 and ≥ 60 years), eligibility criteria (BCS, BCS and BMI > 25, Other), and the type of PA outcome assessed (device-monitored MVPA, self-reported MVPA or MET-hours/week, proportion of BCS meeting guidelines, and other).

## Results

### Study selection and characteristics

**Fig. 1:**
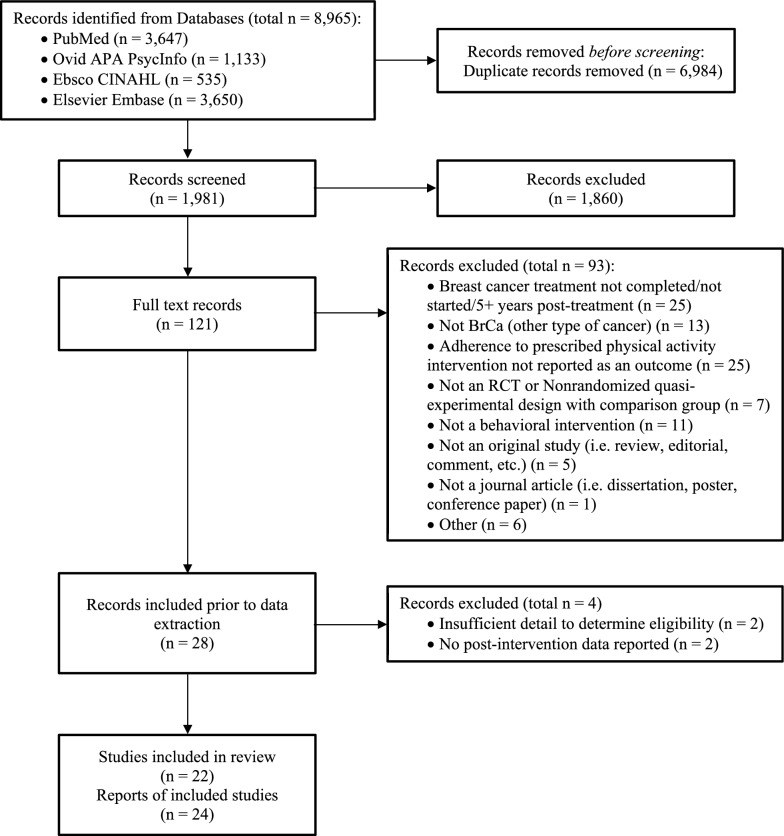
PRISMA-based study selection flowchart

Nearly 2,000 unique items were found during the database searches (Fig. 1). Twenty-eight reports appeared to meet inclusion criteria during full text review, but four reports were excluded during data extraction. Two were excluded due to missing data which prevented the study team from confirming alignment with eligibility criteria [28, 29]. Two were excluded because they reported data beyond the end of the intervention (≥ 3 months) [30, 31], which reflects PA behavioral maintenance [32] rather than PA behavior change, which is the focus of the present analysis. We ultimately included 22 unique RCTs and 24 reports (Table 1). In ten studies, the treatment group was the PA intervention and the control group was usual care [19, 20, 33–40]. In other studies, the control groups were no intervention [41, 42], waitlist [21, 22, 43–45], and attention placebo [46, 47]. In two cases, a group-based PA intervention (treatment) was compared to an individual PA intervention (control) [48, 49], with the group-based intervention being selected as the treatment group and one study compared moderate-intensity to low-intensity PA, with moderate-intensity PA being selected as the treatment group [50]. Nine studies focused on specific subpopulations of post-treatment BCS, including BCS with BMI > 25 kg/m^2^ [33–35, 37, 38], BCS taking aromatase inhibitors (two of which also required participants to report pain) [36, 44, 48], or BCS experiencing fatigue or other symptoms [39, 42].

**Table 1:** Characteristics of participants and physical activity interventions

Study first author (year) ^a^ Country of origin	Sample size ^x^ mean age ^b^ majority race/ethnicity ^c^	Primary intervention components	Intervention setting intervention duration (weeks)	Number (Frequency) of intervention sessions	Theory use reported	Primary method of PA behavior assessment ^d^
Kotte (2025) Sweden	N = 101, 97 ^e^ 55.2 NR	Virtual group exercise	Home 12	24 (2 sessions per week)	NR	Godin Leisure-Time Exercise Questionnaire
Mulero Portela (2025) United States	N = 101, 64 60.9 Hispanic ^f^	Individual training, home exercise equipment and heart rate monitors, culturally adapted workbook	Home, Clinic 24	7 (3 sessions during month 1, followed by 1 session every 4–6 weeks)	TPB	International Physical Activity Questionnaire (IPAQ)- long form
Pinto (2025) United States	N = 61, 60 57.6 White	Fitbit Inspire 2, online behavioral coaching (email or phone)	Home, Clinic 12	12 (1 session per week)	SCT, TTM	ActiGraph® GT3X +
Weemaes (2024) Netherlands	N = 53, 46 ^e^ 53.3 NR	Behavioral coaching (initial in-person intake, remainder by email or phone)	Home 24	15 (12 weekly sessions, 3 monthly sessions)	COM-B	Self-administered log (7-day PA record)
Han (2023) Korea	N = 50, 46 48.9 NR	Supervised exercise program tapered to home-based training; educational workbook and PA log; exercise videos; group-based social capital sessions	Home, Clinic 12	26 (6 weekly in-person supervised sessions, 12 biweekly home-based exercise sessions per week; 8 group social capital sessions across 12 weeks)	SCT	Global Physical Activity Questionnaire (GPAQ)
Puklin (2023) United States	N = 179, 118 g 57.1 White	Pedometer, exercise workbook, self-guided videos	Home 24	No formal sessions; web content released weekly	SCT	Interviewer-administered PA questionnaire
Terranova (2022) Australia	N = 159, 129 55.4 NR	Behavioral counseling-telephone	Home 52	22 (6 weekly calls, 10 biweekly calls, 6 monthly calls)	SCT	ActiGraph® GT3X +
Wang (2021) United States	N = 60, 49 56.0 White	In-person personal training, goal setting	Home, Clinic up to 30 (mean = 18)	3 (1 every 2–16 weeks)	NR	Pedometer (New Lifestyles, Inc. NL-2000i)
Hardcastle (2019, 2023^a^) United Kingdom	N = 66, 66 ^e^ 62.9 White	Behavioral Counseling- phone/video	Home 12	4 required (weeks 1, 2, 4, & 8) and 2 optional (weeks 6 and 10)	HAPA	Actigraph® GT9X +
McNeil (2019) Canada	N = 45, 27 59.0 White	Exercise prescription, Optional phone coaching, PA tracker (Polar A360® accelerometer) and diary	Home 12	No formal sessions	NR	ActiGraph® GT3X +
Lynch (2019) Australia	N = 83, 71 61.6 NR	Activity tracker, At-home exercise, Phone coaching, Workbook	Home, Clinic 12	6 (1 face-to-face session, 2 biweekly phone calls, 1 monthly call)	NR	Actigraph® GT3X +
Leach (2019a, 2019b) United States	N = 27, 24 52.0 White	Education, Group-based supervised exercise	Clinic 8	16 (2 sessions per week)	SCT	International Physical Activity Questionnaire (IPAQ)- short form
Nyrop (2013, 2017^a^) United States	N = 78, 53 63.8 White	Self-directed walking program, workbook	Home 6	No formal sessions	SCT	Self-administered walking log
Desbiens (2017) Canada	N = 26, 15 69.1 NR	Group exercise, Individual exercise with video-based instruction	Home, Clinic 12	24 (2 sessions per week)	NR	Godin Leisure-Time Exercise Questionnaire ^h^
Rogers (2012, 2015^a^, 2023) United States	N = 153, 153 g 54.4 White	Counseling sessions, group-based supervised exercise	Home, Community 12	25 (12 supervised exercise sessions tapered over 6 weeks, 3 in-person counseling sessions every 2 weeks, 6 discussion group sessions spread over 9 weeks)	SCT	Godin Leisure-Time Exercise Questionnaire
Sheppard (2015) United States	N = 31, 22 54.7 African American ^f^	Group exercise, phone coaching, tailored exercise materials	Home, Clinic 12	12 (6 biweekly in-person group exercise and education sessions; 6 individual phone calls on alternating weeks)	SCT, TPB	International Physical Activity Questionnaire (IPAQ)- short form
Mama (2015) United States	N = 89, 53 58.5 Hispanic ^f^	At-home exercise, phone counseling, exercise equipment, optional group-based exercise, tailored newsletters	Home, Community 16	32 required (2 home-based sessions per week) and 4 optional (1 group exercise session per month)	SCT	International Physical Activity Questionnaire (IPAQ)- short form
Harrigan (2015) United States	N = 100, 61 59.0 White	Yamax pedometer, exercise workbook, behavioral counseling (in-person or phone)	Home, Clinic 24	11 (4 weekly sessions, 4 biweekly sessions, 3 monthly sessions)	SCT	Interviewer-administered PA questionnaire
Rock (2013, 2015^a^) United States	N = 692, 506 57.0 White	Home training, group training, phone-based surveys	Home, Community 104	26 required (16 weekly group sessions, 4 biweekly group sessions, 6 monthly group sessions) and 24–38 optional (based on individual needs spread out across the intervention period)	SCT	Godin Leisure-Time Exercise Questionnaire
Irwin (2015^a^), Arem (2016) United States	N = 121, 83 61.0 White	Group-based supervised exercise, individual training	Home, Community 52	104 (2 sessions per week)	NR	Interviewer-administered PA questionnaire
Demark-Wahnefried (2014)	N = 68, 63 ^e^ 61.3 White	Newsletters, optional call line, tailored workbooks	Home 52	No formal sessions	SCT	Godin Leisure-Time Exercise Questionnaire
Lee (2014) Korea	N = 59, 57 42.4 NR	Educational booklets, web-based education	Home, Community 12	No formal sessions	TTM	Self-administered log (7-day PA record) ^i^

^a^ Indicates main outcomes paper used for analysis, in addition to related papers

^b^ Mean age is reported in years. Where not explicitly reported (e.g., provided mean age by treatment arm), overall mean age was estimated based on other data provided

^x^ Sample size reported as number randomized, number analyzed at post-intervention

^c^ Predominant racial/ethnic descriptors applied when participants from a specific group represented 60% or more of the sample. In cases where less than 60% of any one group was represented, the study population was described as a “mix” (MXD) of racial/ethnic groups

^d^ Assessment tool listed provided appropriate data to calculate post-intervention means and standard deviations for effect size estimates unless otherwise noted (see Desbiens 2014; Lee 2014)

^e^ BCS only

^f^ Racial and ethnic terminology reflects the language used in the original study reports

^g^ < 5 years only

^h^ Effect size calculated using categorical outcome data. Participants were classified as insufficiently active, moderately active, and active. Moderately active and active groups were combined and Cohen's d was calculated using the 2 × 2 frequency table method described by Wilson (2017)

^i^ Effect size calculated using categorical outcome data. Participants were classified as meeting PA guidelines or not meeting PA guidelines. Cohen's d was calculated using the 2 × 2 frequency table method described by Wilson (2017)

*COM-B* Capability, Opportunity, Motivation-Behavior theory, *HAPA* Health Action Process Approach, *NR* not reported, *PA* physical activity, *SCT* Social Cognitive Theory, *TPB* Theory of Planned Behavior, *TTM* Transtheoretical Model of Behavior Change

### Study population and description

A total of 2,390 BCS were included across the 22 studies (mean sample size = 109, range = 26–692). Thirteen RCTs were conducted in the United States, two of which were conducted partially or fully in Puerto Rico; two in Australia; two in Canada; two in Korea; one in the Netherlands, one in Sweden, and one in the United Kingdom. Out of those who reported race or ethnicity data (k = 14), most included majority White BCS (> 60% of sample) (k = 12). The two other studies recruited BCS who self-identified as African American [38] or Mexican–American or Puerto Rican [22, 50]. All included BCS were female, and the weighted mean age of participants was 57 years (range = 42–69). Reporting of education and income data was inconsistent across studies.

Cancer treatment characteristics (e.g., stage at diagnosis, treatment type) were largely similar across studies. Most samples (k = 16) were of mixed stage at diagnosis (i.e., ≥ 60% of the sample was *not* diagnosed with the same stage). Two studies recruited primarily BCS with Stage I disease [36, 47] and one study reported the number of participants with metastases in lymph nodes [42] and three studies did not report staging data [19, 40, 43]. It is also important to note that BCS with Stage IV disease were excluded from all but 4 studies [22, 42–44]. Three papers did not include data on treatment type [22, 34, 38]. BCS included in the remaining studies underwent a combination of surgery, chemotherapy, and/or radiation treatment.

### Intervention design

Intervention delivery settings were mostly home-based mixed with clinic-based or community-based settings. All but one study included a home-based component [49]. Twelve studies took place in a mixed setting including sessions in a clinic or community setting combined with home settings [20–22, 35–39, 43, 47, 48, 50]. Nine interventions were entirely home-based [19, 33, 34, 40–42, 44–46]. Participants in these studies developed PA goals and carried out these goals at home.

There was a wide range of intervention durations, number and frequency of sessions, length of sessions, and level of supervision. The most frequent intervention duration was 12 weeks (range: 6 to 104 weeks). The number of intervention sessions ranged from 0 to 50 sessions lasting 10 to 120 min long. Seven interventions started with high frequency contact (1-3x/week) and tapered off to low (e.g., 1 session per month) to no contact [19–21, 33, 35, 37, 42]. Seven interventions held a consistent schedule of one [47] or two times per week [36, 40, 48, 49] or once every two weeks [22, 38]. Wang et al. (2021) delivered 3 sessions over up to 30 weeks [43] and Mulero Portela delivered 7 sessions across 6 months (3 in month 1) [50]. In one study, the intervention consisted of 1 in-person supervised exercise session per week for 6 weeks then progressed to 2 home-based exercise sessions per week for 12 weeks and 8 small group social capital sessions across the intervention duration [39]. Five studies held no formal intervention session [34, 41, 44–46]. Eight interventions were classified as high level of supervision [20, 36, 38–40, 43, 48, 49], ten as medium [19, 21, 22, 33, 35, 37, 41, 42, 47, 50], and four as low [34, 44–46].

A variety of intervention strategies were reported. Seven studies focused on multiple behavior change (PA, diet), with a primary intervention target of weight loss [33–35, 37, 38, 45, 46]. Behavioral targets in these studies included both increases in PA and dietary changes such as eating 5 or more servings of fruits and vegetables per day, limiting fat intake, or reducing overall caloric intake. Most studies allowed participants to choose their preferred PA modality. In 15 cases, PA targets aligned with aerobic PA guidelines [7, 8] of achieving 150 min of aerobic MVPA per week. Nine studies also incorporated weight training [22, 33, 36, 39, 40, 43, 48–50]. Eight interventions included supervised exercise either in individual [36, 39, 49] or group [20, 22, 36, 38, 40, 48, 49] format. Seven interventions provided pedometers, heart rate monitors, or activity trackers for participants to self-monitor PA [19, 33, 37, 41, 45, 47, 49]. Behavioral counseling was included in 9 interventions. Counseling was mostly delivered individually over the phone, but one study also offered video sessions [19], and three studies delivered behavioral content in-person in group settings [20, 35, 38]. Sixteen out of the twenty-two studies reported the use of behavioral theory to guide their intervention [19, 20, 22, 33–35, 37–39, 42, 44–47, 49, 50]. The most utilized theoretical model was the Social Cognitive Theory [20, 22, 31, 33–35, 37, 39, 44, 45, 47] to enhance self-efficacy, outcome expectations, and/or self-management strategies to support behavior change. Other behavioral theories utilized included the Health Action Process Approach [19], the Theory of Planned Behavior [38, 50], the Transtheoretical Model of Behavior Change [46], and the COM-B Model for Behavior Change [42].

### Methods of PA assessment

PA outcomes included in our meta-analysis were assessed using both self-report (k = 16) and device-monitoring (k = 6). Self-reported data primarily came from validated surveys including the International Physical Activity Questionnaire-short form (IPAQ-SF) (k = 4) [22, 31, 38, 50],the Godin Leisure-Time Exercise Questionnaire (k = 5) [20, 34, 35, 40, 48], and the Global Physical Activity Questionnaire (k = 1) [39]. Three studies utilized an interviewer administered PA questionnaire, [36, 37, 45] and three studies utilized an exercise log [42, 44, 46]. Device-monitored data came from accelerometry [19, 21, 33, 41, 47] and pedometers [43]. PA outcomes included minutes of MVPA per week [19–21, 34–37, 40–42, 45, 47], number of steps per day [43], activity category [48], activity counts [33], metabolic equivalent hours per week [39, 49], proportion of participants meeting PA guidelines [46], total PA minutes per week [38], total leisure-time PA [50], and number of walking minutes per week [44].

### Risk of bias assessment

Most studies had low risk for bias, but there were some concerns for three studies. The primary concerns were related to lack of reported blinding of participants and/or assessors, potentially introducing a risk of bias in judging results. To determine if these variations produced bias, we conducted a preliminary analysis with all studies compared to an analysis with the three studies in question removed (data not reported). This did not change the result; therefore, we elected to keep the studies in the sample for final analysis.

### Meta-analysis

**Fig. 2:**
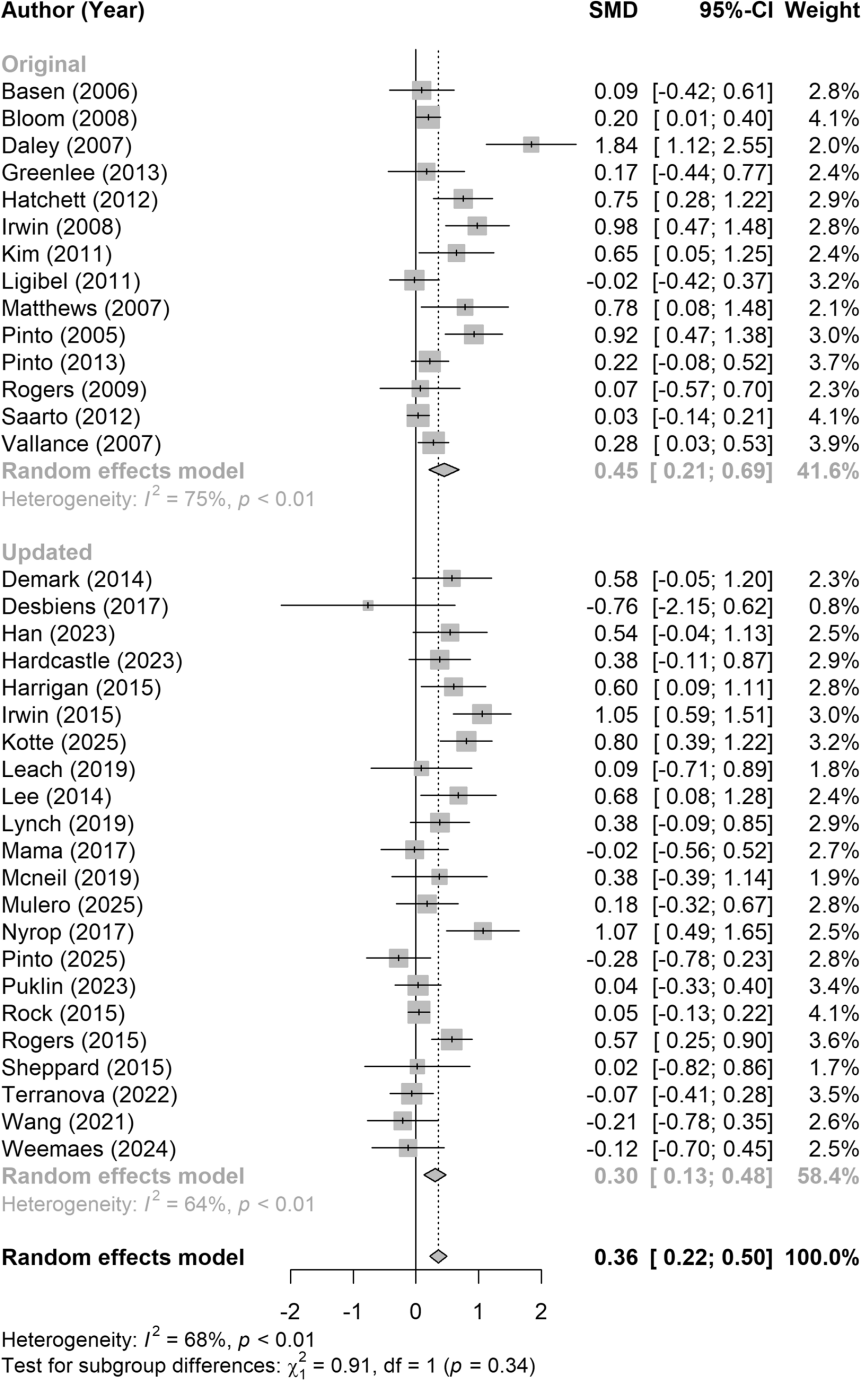
Forest plot illustrating the standardized mean difference (SMD; Cohen’s d) in post-intervention physical activity outcomes between intervention and control groups, with 95% confidence intervals (CIs) including studies from the 2015 review* and the updated review. SMDs were calculated following the methods described by Wilson (2017) [27]. Data were pooled using a random-effects model, and statistical significance was assessed using a Z-test. Each gray box represents the SMD for an individual study, with horizontal lines indicating the corresponding 95% CI. The vertical dotted line denotes the pooled effect estimate, and the gray diamond represents its 95% CI. Positive values indicate a favorable effect in the direction of the intervention group. *data for Pinto 2013 was updated since the publishing of the 2015 review

**Fig. 3:**
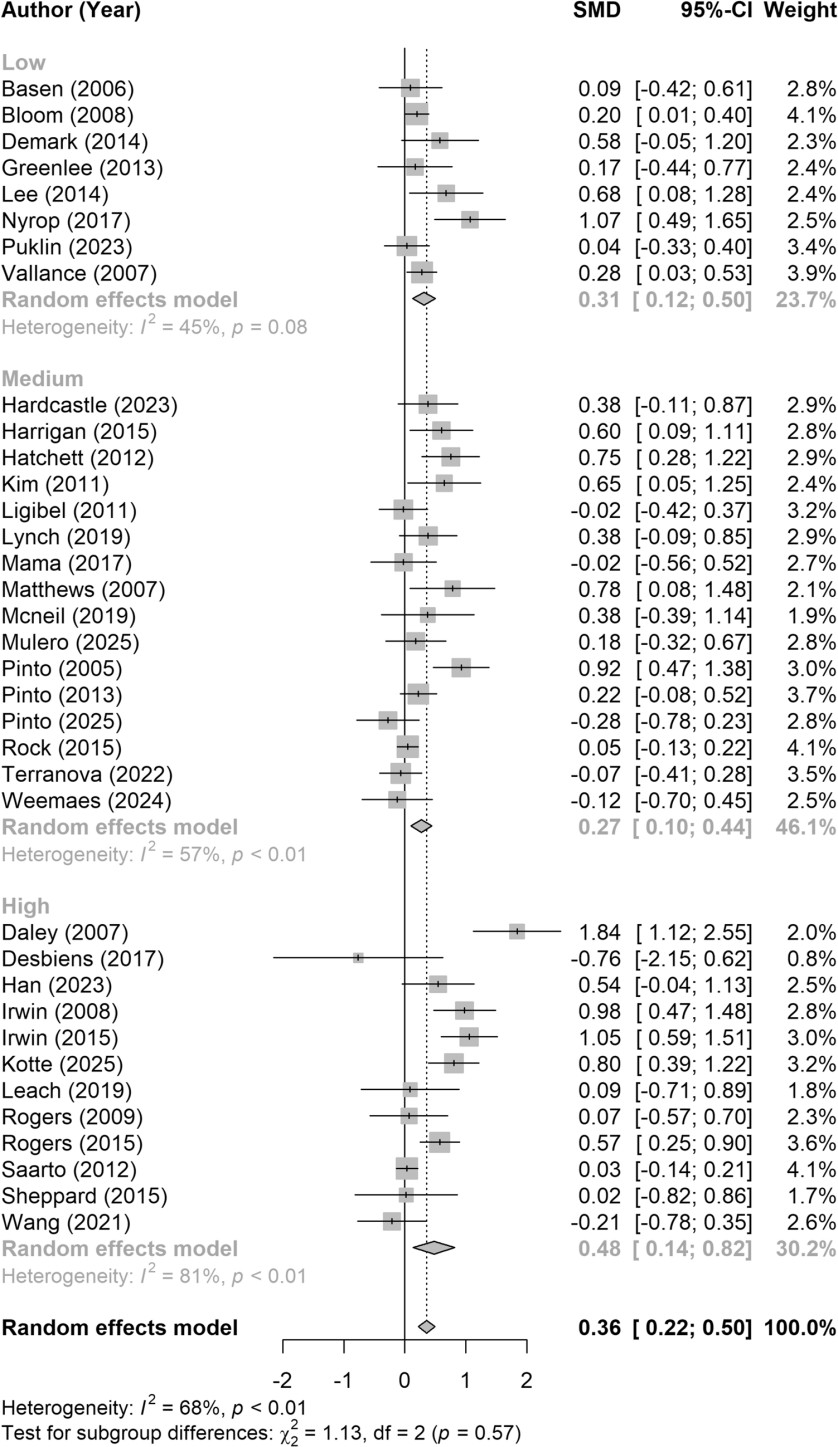
Forest plot illustrating the subgroup effects of supervision level on the intervention’s impact on physical activity outcomes. Studies were categorized into three levels of supervision: low (minimal or no physical activity oversight), medium (behavioral counseling without structured physical activity), and high (multi-component interventions including structured interactions and/or supervised physical activity). Standardized mean differences (SMD; Cohen’s d) with 95% confidence intervals (CIs) were calculated using the methods described by Wilson (2017) [27]. Data were pooled within each subgroup using a random-effects model, and subgroup differences were tested using a chi-squared test. Each gray box represents the SMD for an individual study, with horizontal lines indicating the corresponding 95% CI. Gray diamonds indicate the pooled effect and 95% CI for each subgroup and the overall effect. The vertical dotted line represents the overall pooled effect across all studies. Positive values reflect a favorable effect in the direction of the intervention group

The average overall SMD for all studies, including the original and updated, was 0.36 with a p < 0.01 (Fig. 2), indicating a small-to-moderate overall effect. Effect sizes for individual studies from the original review can be found in the original report [14]. Effect sizes for the updated search ranged from − 0.76. to 1.07. Out of 22 studies, 11 positively favored the treatment group and 6 were statistically significant. Eight demonstrated negligible intervention effects (absolute value of d < 0.20), and three favored the control group but were not statistically significant. In alignment with the original meta-analysis, results for level of supervision are presented in Fig. 3. There was no significant subgroup effect of level of supervision, nor for all other tested subgroups (p > 0.05). The remaining subgroup results are presented in Online Resource 3.

## Discussion

This review provides an updated meta-analysis and synthesis of literature examining the effectiveness of behavioral PA interventions among BCS ≤ 5 years following cancer treatment. Twenty-two RCTs were identified in our search, including 2,390 participants across 7 countries. Intervention strategies included individual and group-based exercise, behavioral counseling, educational materials, and a combination of these. The meta-analysis revealed small to large effects across individual studies, with an overall small effect in favor of the intervention. 

Included participants in the present analysis were older (mean age 57 vs. 49 years), and more studies enrolled rural or racially and ethnically diverse populations compared to the original review. United States population is aging and growing more diverse [51], a demographic shift that is reflected in cancer survivors [52]. In fact, older adults (i.e., 65 years or older) now represent more than two thirds of all survivors [53] and it is projected that the population of survivors from racially and ethnically underserved groups will grow by 99%, whereas a 31% increase is projected for non-Hispanic white groups [52]. Only 36% of participants in cancer clinical trials are 65 years or older [4] and survivors from racial and ethnic minoritized groups remain underrepresented [54]. Although several studies included underserved and racially diverse participants, only a few were specifically tailored to these populations. Tailoring interventions to diverse groups may improve relevance and effectiveness, while still ensuring designs remains appropriate for studies with larger proportions of certain populations. The present findings reflect a positive shift, but greater efforts are needed to study populations representative of the cancer survivor population at large.

It is interesting to note that, contrary to the demographic makeup of participants, study and intervention characteristics were mostly similar between the present and original findings. All but one intervention in the present analysis included a home-based component. Home-based exercise programs may be more accessible, particularly for post-treatment cancer survivors [55]. A rise in technology-enhanced interventions may continue to contribute to the feasibility and accessibility of home-based physical activity interventions. It is important to note that our analyses revealed no significant influence of level of supervision on the intervention effect, suggesting that frequent contact between interventionists or researchers may not be necessary to support behavior change. This is encouraging as it suggests flexibility in the types and amounts of strategies and interventions used to promote PA behavior change may not influence behavioral outcomes.

The overall effect was similar for the present analysis compared to the previous analysis (d = 0.47, 95% CI [0.23, 0.67]). It is interesting to note that two studies in the present analysis compared individual to group-based PA interventions [48, 49], whereas all studies in the previous review had non-exercise control conditions. Comparing two intervention strategies reflects a recognition of the importance of PA among post-treatment BCS. Researchers are beginning to test different strategies to determine the most effective way to promote PA among post-treatment BCS. The progression to testing different strategies, combined with the increase in number of studies, could suggest a greater interest in building PA into cancer recovery plans. We plan to conduct a deeper analysis of behavior theory and its impact on PA behavior change as a next step in this line of research to better support the integration of PA into cancer recovery plans.

We note several shifts in the literature since the updated review, yet more questions remain. Although we did observe more studies with underserved populations (older, rural-dwelling, racially and ethnically diverse), more research is needed in these areas. It is important to highlight ongoing or upcoming work that was found in our search but did not meet inclusion criteria, including studies of rural [56, 57], underserved racial and ethnic groups [58–60], and older [61, 62] BCS. These studies are reflective of the continued promising trajectory of PA-related research among BCS. There was limited inclusion of BCS with stage IV disease, however, and there remains a gap in understanding the effectiveness of behavioral PA interventions among the growing population of metavivors (i.e., individuals living with advanced cancer) [63].

The present analysis also does not reflect current trends in translational research, including trials which include biomarkers and digital and virtual formats, including AI approaches [64]. There is rising interest in precision medicine, or tailored approaches based on individual patient factors to target a desired health outcome [65]. Including biomarkers in this body of work would bridge understanding between behavioral and biological factors, thereby broadening the mechanistic understanding of this body of work. This is important not only for behavioral outcomes, but for the impact of changing behavior on health outcomes such as reducing fatigue and pain or improving function, which are common long-term symptoms experienced by BCS. As technology advances there is increasing interest in understanding its utility for broadening the feasibility and scalability of participating in PA interventions. These could potentially be areas of interest in the future to continue to evolve to meet the needs of cancer survivors.

Strengths and limitations of the present analysis should be discussed. Strengths include the large sample of studies that met our eligibility criteria, rigorous methodology, and the inclusion of the bias assessment (ROB 2). Included studies reflect international and domestic research and include diverse populations that are more reflective of the wider BCS population than previously included. However, like all systematic reviews and meta-analyses, we could only analyze what was available in the studies, leading to an inherent risk of bias. Some studies were missing key details, and we attempted to clarify these details with the study team. Methodological changes to the updated review enhanced the rigor and reproducibility of this work, however, may affect the consistency in findings between the original and updated review papers. It is also important to note that the review period spanned the COVID-19 global pandemic, a period when research was paused, refocused, and/or adapted for remote delivery. Changes in intervention or study design, and ongoing challenges with the sociopolitical environment or access to safe spaces to PA engagement may have affected the results of these studies, introducing some historical bias into our results.

Some practical implications for strengthening the reporting of future research can be gleaned from this work. Providing clearer reporting of intervention components and delivery would help improve transparency and reproducibility of behavioral interventions. Using established reporting guidelines, such as CONSRT [66] or TIDieR [67], would be beneficial. Similarly, parsing out the aerobic and muscle strengthening components of the PA guidelines would enhance precision. Not only would this enhance reporting quality, but it would facilitate future efforts in synthesizing intervention components, allowing for better quantification of the impact of individual intervention components on the intervention effect. Because of the growing recognition of the importance of PA for post-treatment BCS, it is possible that the definition of usual care is shifting to providing PA guidance in survivorship care plans. Providing more details to allow for systematic recording of usual care conditions would be beneficial. Adding contextual detail, such as study setting, geographical locations, and population characteristics would aid in interpretation of the findings across diverse participant groups, as these factors are important in understanding the feasibility and generalizability of this body of work.

## Conclusion

Adopting or maintaining a physically active lifestyle facilitates longevity, health, and well-being during the transition from active to long-term recovery after breast cancer treatment. The present systematic review and meta-analysis provided an updated review of RCTs published between 2013 and 2025 testing interventions designed to increase PA among recent post-treatment BCS. Overall, the data suggests interventions have a modest, positive influence on PA levels. The findings also offer insights into the progress of the field over the past 10, such as a limited increased focus on older, more diverse populations. We highlight a need to continue this trajectory and comment on potential trends in the field such as technology-enhanced interventions and incorporation of biomarkers.

## Supplementary Information

Below is the link to the electronic supplementary material.

Supplementary file1 (PDF 394 KB)

Supplementary file2 (PDF 98 KB)

Supplementary file3 (PDF 1147 KB)

## Data Availability

The datasets generated during and/or analyzed during the current study are not publicly available to preserve privacy and intellectual property but are available from the corresponding author on reasonable request.
