# Supplementary material for: Updated systematic review and meta-analysis: taking the next step in physical activity behavioral interventions for post-treatment breast cancer survivors
Source: Breast Cancer Res Treat. 2026 Jan 10;215(2):56. doi: 10.1007/s10549-025-07892-3 (PMC12790553; doi:10.1007/s10549-025-07892-3)
Supplement: Supplementary file 1 — Supplementary file1 (PDF 394 KB) [file 10549_2025_7892_MOESM1_ESM.pdf]

Online Resource 1: Search Strategy

Article Title: Updated systematic review and meta-analysis: taking the next step in physical activity behavioral interventions for post-treatment breast cancer survivors

Journal name: Breast Cancer Research and Treatment

Authors: Brianna N Leitzelar[a,b], Alana R. Willis[c], Sarah N. Price[b], Janet A. Tooze[c], Helena M. VonVille[d], Rachel Lintz[e], Shirley M. Bluethmann[b]

Affiliations:
[a] School of Kinesiology, University of Minnesota-Twin Cities, Minneapolis, MN, USA
[b] Department of Social Sciences and Health Policy, Wake Forest University School of Medicine, Winston-Salem, NC, USA
[c] Department of Biostatistics and Data Science, Wake Forest University School of Medicine, Winston-Salem, NC, USA
[d] Health Sciences Library System, University of Pittsburgh, Pittsburgh, PA, USA
[e] Department of Public Health Sciences, Penn State College of Medicine, Hershey, PA, USA

Corresponding Author:
Brianna N. Leitzelar
Email: leitz025@umn.edu

Online Resource Contents

Table 1: Summary of Literature Databases Searched....................2
- Table 1a: PubMed search strategy....................3
- Table 1b: APA PsycInfo® search strategy....................16
- Table 1c: Embase® search strategy....................28
- Table 1d: CINAHL® search strategy....................44

The template is based on:

Niederstadt C, Droste S. Reporting and presenting information retrieval processes: the need for optimizing common practice in health technology assessment. Int J Technol Assess Health Care. 2010;26(4):450-7.

And follows the reporting principles recommended in PRISMA-S:
Rethlefsen ML, Kirtley S, Waffenschmidt S, Ayala AP, Moher D, Page MJ, Koffel JB; PRISMA-S Group. PRISMA-S: an extension to the PRISMA Statement for Reporting Literature Searches in Systematic Reviews. Syst Rev. 2021 Jan 26;10(1):39. doi: 10.1186/s13643-020-01542-z. PMID: 33499930; PMCID: PMC7839230.

Deduplication methods:

Bramer WM, Giustini D, de Jonge GB, Holland L, Bekhuis T. De-duplication of database search results for systematic reviews in EndNote. J Med Libr Assoc. 2016 Jul;104(3):240-3. doi: 10.3163/1536-5050.104.3.014
René Otten, Ralph de Vries, & Linda Schoonmade. (2019, December 12). Amsterdam Efficient Deduplication (AED) method - manual. Zenodo.org . http://doi.org/10.5281/zenodo.3741885

Table 1: Summary of Literature Databases Searched

Table	Vendor/ Interface	Database	Date searched	Database update	Searcher(s)
1a	National Library of Medicine (US)	PubMed	June 25, 2020; update May 5, 2022; update March 20, 2023; update October 13, 2025	June 25, 2020; update May 5, 2022; update March 20, 2023; update October 11, 2025 (note: gov't shutdown impacted processing of records	Helena M. VonVille
1b	Ovid	APA PsycInfo®	June 22, 2020; updated May 5, 2022; updated March 20, 2023; update October 13, 2025	1806 to June Week 3 2020; update: 1806 to April Week 4 2022; update: 1806 to March Week 2 2023; updated: 1806 to October Week 1 2025	Helena M. VonVille
1c	Elsevier	Embase	June 25, 2020; update 1: May 5, 2022; update 2: March 20, 2023; update October 13, 2025	June 25, 2020; update 1: May 5, 2022; update 2: March 20, 2023; update 3: October 13, 2025	Helena M. VonVille
1d	Ebsco	CINAHL	June 25, 2020	June 25, 2020	Helena M. VonVille

Table 1a: PubMed search strategy

Provider/Interface	National Library of Medicine
Database	PubMed
Date searched	June 25, 2020; update May 5, 2022; update March 20, 2023; update October 13, 2025
Database update	June 25, 2020; update May 5, 2022; update March 20, 2023; update October 11, 2025 (note: gov't shutdown impacted processing of records
Search developer(s)	Helena M. VonVille
Limit to English	Yes
Date Range	2013:2020; update 2013:3000
Publication Types	No limit by publication type
Search filter source	https://hsls.libguides.com/PubMed-search-filters/experimental-studies Note: The filters are no longer available in their original form and have been updated.

**June 25, 2020 search**
(((("Breast Cancer Lymphedema"[mesh:noexp] OR "Breast Neoplasms"[mesh:noexp] OR "Carcinoma, Ductal, Breast"[mesh:noexp] OR "Hereditary Breast and Ovarian Cancer Syndrome"[Mesh] OR "Triple Negative Breast Neoplasms"[Mesh:noexp]) OR ((breast[tiab] AND (cancer*[tiab] OR carcinoma*[tiab] OR neoplasm*[tiab] OR tumor*[tiab] OR tumour*[tiab])))) AND (("cancer survivors"[mesh:noexp] OR survivors[mesh:noexp]) OR (survivor*[tiab]))) AND (("Circuit-Based Exercise"[mesh:noexp] OR "Dance Therapy"[mesh:noexp] OR "Endurance Training"[mesh:noexp] OR "Exercise"[mesh:noexp] OR "Exercise Therapy"[Mesh:NoExp] OR "Exercise Tolerance"[mesh:noexp] OR "Gymnastics"[mesh:noexp] OR "High-Intensity Interval Training"[mesh:noexp] OR "Jogging"[mesh:noexp] OR "Motion Therapy, Continuous Passive"[Mesh:NoExp] OR "Motor Activity"[mesh:noexp] OR "Muscle Stretching Exercises"[mesh:noexp] OR "Physical Conditioning, Human"[mesh:noexp] OR "Physical Endurance"[mesh:noexp] OR "Plyometric Exercise"[mesh:noexp] OR "Qigong"[mesh:noexp] OR "Resistance Training"[mesh:noexp] OR "Running"[mesh:noexp] OR "Stair Climbing"[mesh:noexp] OR "Swimming"[mesh:noexp] OR "Tai Ji"[mesh:noexp] OR "Walking"[mesh:noexp] OR "Yoga"[mesh:noexp]) OR (aerobics[tiab] OR ballet[tiab] OR bicycling[tiab] OR biking[tiab] OR calisthenics[tiab] OR "circuit training"[tiab] OR dance[tiab] OR exercise[tiab] OR physical activit*[tiab] OR "Physical Endurance"[tiab] OR "Plyometric*"[tiab] OR running[tiab] OR swimming[tiab] OR "tai chi"[tiab] OR "tai ji"[tiab] OR walking[tiab] OR yoga[tiab] OR ((strength[tiab] OR resistance[tiab]) AND training[tiab])))) AND (("Clinical Trial" [PTYP:NoExp] OR "Adaptive Clinical Trial" [PTYP:NoExp] OR "clinical trial, phase ii"[PTYP] OR "clinical trial, phase iii"[PTYP] OR "clinical trial, phase iv"[PTYP] OR "controlled clinical trial"[PTYP] OR "multicenter study"[PTYP] OR "randomized controlled trial"[PTYP] OR "Clinical Trials as Topic"[mesh:noexp] OR "clinical trials, phase ii as topic"[MeSH:noexp] OR "clinical trials, phase iii as topic"[MeSH:noexp] OR "clinical trials, phase iv as topic"[MeSH:noexp] OR "controlled clinical trials as topic"[MeSH:noexp] OR "Non-Randomized Controlled Trials as Topic"[MeSH:noexp] OR "randomized controlled trials as topic"[MeSH:noexp] OR "Equivalence Trials as Topic"[MeSH:noexp] OR "Intention to Treat Analysis"[MeSH:noexp] OR "Pragmatic Clinical Trials as Topic"[MeSH:noexp] OR "early termination of clinical trials"[MeSH:noexp] OR "multicenter studies as topic"[MeSH:noexp] OR “Double-Blind Method”[Mesh] OR "phase II"[tiab] OR "phase III"[tiab] OR "phase IV"[tiab] OR "phase 2"[tiab] OR "phase 3"[tiab] OR "phase 4"[tiab] OR ((randomised[TIAB] OR randomized[TIAB]) AND (trial[TIAB] OR trials[tiab])) OR ((single[TIAB] OR double[TIAB] OR doubled[TIAB] OR triple[TIAB] OR tripled[TIAB] OR treble[TIAB] OR treble[TIAB]) AND (blind*[TIAB] OR mask*[TIAB])) OR ("4 arm"[tiab] OR "four arm"[tiab])) OR (evaluation study[PT:NOEXP] OR "evaluation studies as topic"[MESH:NOEXP] OR "program evaluation"[MESH:NOEXP] OR "validation study"[PT:NOEXP] OR "validation studies as topic"[MESH:NOEXP] OR (pre-[TIAB] AND post-[TIAB]) OR (pretest[TIAB] AND posttest[TIAB]) OR (program*[TIAB] AND (evaluate[TIAB] OR evaluated[TIAB] OR evaluates[TIAB] OR evaluating[TIAB] OR evaluation[TIAB] OR evaluations[TIAB] OR evaluator[TIAB] OR

evaluators[TIAB])) OR effectiveness[TIAB] OR intervention[TIAB])) AND English[la] AND 2013:2020[dp]
**May 2022 Updated search with items from previous search removed**
(((("Breast Cancer Lymphedema"[mesh:noexp] OR "Breast Neoplasms"[mesh:noexp] OR "Carcinoma, Ductal, Breast"[mesh:noexp] OR "Hereditary Breast and Ovarian Cancer Syndrome"[Mesh] OR "Triple Negative Breast Neoplasms"[Mesh:noexp]) OR ((breast[tiab] AND (cancer*[tiab] OR carcinoma*[tiab] OR neoplasm*[tiab] OR tumor*[tiab] OR tumour*[tiab])))) AND (("cancer survivors"[mesh:noexp] OR survivors[mesh:noexp]) OR (survivor*[tiab]))) AND (("Circuit-Based Exercise"[mesh:noexp] OR "Dance Therapy"[mesh:noexp] OR "Endurance Training"[mesh:noexp] OR "Exercise"[mesh:noexp] OR "Exercise Therapy"[Mesh:NoExp] OR "Exercise Tolerance"[mesh:noexp] OR "Gymnastics"[mesh:noexp] OR "High-Intensity Interval Training"[mesh:noexp] OR "Jogging"[mesh:noexp] OR "Motion Therapy, Continuous Passive"[Mesh:NoExp] OR "Motor Activity"[mesh:noexp] OR "Muscle Stretching Exercises"[mesh:noexp] OR "Physical Conditioning, Human"[mesh:noexp] OR "Physical Endurance"[mesh:noexp] OR "Plyometric Exercise"[mesh:noexp] OR "Qigong"[mesh:noexp] OR "Resistance Training"[mesh:noexp] OR "Running"[mesh:noexp] OR "Stair Climbing"[mesh:noexp] OR "Swimming"[mesh:noexp] OR "Tai Ji"[mesh:noexp] OR "Walking"[mesh:noexp] OR "Yoga"[mesh:noexp]) OR (aerobics[tiab] OR ballet[tiab] OR bicycling[tiab] OR biking[tiab] OR calisthenics[tiab] OR "circuit training"[tiab] OR dance[tiab] OR exercise[tiab] OR physical activit*[tiab] OR "Physical Endurance"[tiab] OR "Plyometric*"[tiab] OR running[tiab] OR swimming[tiab] OR "tai chi"[tiab] OR "tai ji"[tiab] OR walking[tiab] OR yoga[tiab] OR ((strength[tiab] OR resistance[tiab]) AND training[tiab])))) AND (("Clinical Trial" [PTYP:NoExp] OR "Adaptive Clinical Trial" [PTYP:NoExp] OR "clinical trial, phase ii"[PTYP] OR "clinical trial, phase iii"[PTYP] OR "clinical trial, phase iv"[PTYP] OR "controlled clinical trial"[PTYP] OR "multicenter study"[PTYP] OR "randomized controlled trial"[PTYP] OR "Clinical Trials as Topic"[mesh:noexp] OR "clinical trials, phase ii as topic"[MeSH:noexp] OR "clinical trials, phase iii as topic"[MeSH:noexp] OR "clinical trials, phase iv as topic"[MeSH:noexp] OR "controlled clinical trials as topic"[MeSH:noexp] OR "Non-Randomized Controlled Trials as Topic"[MeSH:noexp] OR "randomized controlled trials as topic"[MeSH:noexp] OR "Equivalence Trials as Topic"[MeSH:noexp] OR "Intention to Treat Analysis"[MeSH:noexp] OR "Pragmatic Clinical Trials as Topic"[MeSH:noexp] OR "early termination of clinical trials"[MeSH:noexp] OR "multicenter studies as topic"[MeSH:noexp] OR “Double-Blind Method”[Mesh] OR "phase II"[tiab] OR "phase III"[tiab] OR "phase IV"[tiab] OR "phase 2"[tiab] OR "phase 3"[tiab] OR "phase 4"[tiab] OR ((randomised[TIAB] OR randomized[TIAB]) AND (trial[TIAB] OR trials[tiab])) OR ((single[TIAB] OR double[TIAB] OR doubled[TIAB] OR triple[TIAB] OR tripled[TIAB] OR treble[TIAB] OR treble[TIAB]) AND (blind*[TIAB] OR mask*[TIAB])) OR ("4 arm"[tiab] OR "four arm"[tiab])) OR (evaluation study[PT:NOEXP] OR "evaluation studies as topic"[MESH:NOEXP] OR "program evaluation"[MESH:NOEXP] OR "validation study"[PT:NOEXP] OR "validation studies as topic"[MESH:NOEXP] OR (pre-[TIAB] AND post-[TIAB]) OR (pretest[TIAB] AND posttest[TIAB]) OR (program*[TIAB] AND (evaluate[TIAB] OR evaluated[TIAB] OR evaluates[TIAB] OR evaluating[TIAB] OR evaluation[TIAB] OR evaluations[TIAB] OR evaluator[TIAB] OR evaluators[TIAB])) OR effectiveness[TIAB] OR intervention[TIAB])) AND English[la] AND 2013:3000[dp] NOT (26869680 OR 32535764 OR 23378138 OR 24176784 OR 29409128 OR 23635341 OR 25645134 OR 30694978 OR 31014267 OR 26068412 OR 30982113 OR 29746264 OR 29168064 OR 27490111 OR 23274615 OR 30368741 OR 26303657 OR 30340503 OR 31309977 OR 23529000 OR 24043292 OR 27738306 OR 27416835 OR 31791246 OR 29260391 OR 22822181 OR 26327866 OR 25171662 OR 27543047 OR 28666465 OR 24644304 OR 27524376 OR 23293010 OR 30320304 OR 26801931 OR 26703650 OR 27312845 OR 31759342 OR 24212124 OR 28632240 OR 26713501 OR 26315617 OR 31144265 OR 29057279 OR 27988868 OR 30789349 OR 28922074 OR 30554276 OR 24633590 OR 30912011 OR 30445595 OR 28470368 OR 26840439 OR 29498250 OR 30609982 OR 31168711 OR 31264183 OR 23766391 OR 25903195 OR 31475306 OR 22535636 OR 25148839 OR 31617564 OR 22879576 OR 28525464 OR 29404842 OR 24419734 OR 23538987 OR 29086523 OR 23579251 OR 29617707 OR 28721384 OR 30663156 OR 30862228 OR 29869823 OR 30562305 OR 31041685 OR 26769117 OR 30554923 OR 27873046 OR 28736768 OR 26000593 OR 26640827 OR 24707505 OR 27857276 OR 26935995 OR 29644889 OR 23439424 OR 30067063 OR

25576214 OR 27146839 OR 25920528 OR 24805910 OR 30400784 OR 25179578 OR 25672583 OR
28070770 OR 24804802 OR 26970957 OR 25708515 OR 27646264 OR 28588911 OR 28347291 OR
28624949 OR 27565830 OR 27838922 OR 26679449 OR 29082588 OR 25394834 OR 23612029 OR
25132146 OR 31600424 OR 30972643 OR 25614924 OR 31206630 OR 28894513 OR 24648018 OR
22672363 OR 25833781 OR 26984227 OR 32130627 OR 30339481 OR 26286486 OR 25602030 OR
25567329 OR 27531024 OR 28636526 OR 30485297 OR 23915045 OR 30543479 OR 29243165 OR
23519041 OR 24310809 OR 26850265 OR 26395825 OR 27925359 OR 28075038 OR 23430010 OR
25313756 OR 28639097 OR 23876573 OR 31853701 OR 28026901 OR 23213072 OR 24319454 OR
27197276 OR 30767964 OR 28427925 OR 31415063 OR 31444192 OR 25965782 OR 31227537 OR
31605312 OR 26820653 OR 26199085 OR 32348688 OR 27893938 OR 26175059 OR 25814054 OR
25730596 OR 31028212 OR 23017985 OR 31087419 OR 29409922 OR 29880779 OR 30874927 OR
24869977 OR 31689771 OR 31804383 OR 28745131 OR 27539586 OR 27530961 OR 28702218 OR
22831916 OR 31617073 OR 26880252 OR 29356607 OR 23184120 OR 28414894 OR 27821958 OR
27635602 OR 27295811 OR 27161493 OR 32019093 OR 28417203 OR 27893212 OR 31173341 OR
27110131 OR 27258052 OR 24337598 OR 27742668 OR 28178355 OR 29026725 OR 29470705 OR
29961039 OR 23963636 OR 29936464 OR 23989030 OR 25337546 OR 29259819 OR 32443355 OR
29654416 OR 29470804 OR 23749481 OR 30980431 OR 27516182 OR 29141853 OR 30077574 OR
27417791 OR 25781872 OR 25853030 OR 25181937 OR 31236699 OR 31763746 OR 25417174 OR
32404725 OR 29076388 OR 25980790 OR 28551083 OR 23906102 OR 26373696 OR 32307828 OR
28410171 OR 24222132 OR 30874369 OR 30840336 OR 23326466 OR 28965138 OR 27470258 OR
32314110 OR 22573338 OR 28392934 OR 28717378 OR 29943097 OR 23575310 OR 25899303 OR
26782031 OR 26543382 OR 23860950 OR 24894145 OR 25724409 OR 29511965 OR 23620675 OR
28639157 OR 31121916 OR 27883168 OR 32020356 OR 31998651 OR 27601139 OR 31994181 OR
26016834 OR 25711667 OR 25845088 OR 31454519 OR 26543789 OR 23244677 OR 32510461 OR
24596359 OR 26925999 OR 27912947 OR 28150044 OR 29645327 OR 25975457 OR 26677207 OR
32415529 OR 30864301 OR 26655430 OR 23620504 OR 32249355 OR 24495696 OR 25681782 OR
31290395 OR 28228661 OR 29471477 OR 28665541 OR 30117760 OR 27737976 OR 23615146 OR
26756114 OR 28620700 OR 28161788 OR 27192633 OR 31319398 OR 26512712 OR 32234652 OR
30488344 OR 30723698 OR 26304504 OR 30850143 OR 32111767 OR 25349070 OR 29847241 OR
28991039 OR 31354000 OR 30912010 OR 26586495 OR 25901376 OR 30500981 OR 29932460 OR
28836379 OR 30233121 OR 25037667 OR 27498099 OR 23177321 OR 25749601 OR 22996743 OR
26945570 OR 29587682 OR 28253845 OR 27840340 OR 30658629 OR 32232722 OR 28189100 OR
26427563 OR 31244536 OR 28624715 OR 27334210 OR 32304828 OR 27981118 OR 32306100 OR
27718533 OR 32298407 OR 23026681 OR 26855719 OR 32031221 OR 24916951 OR 32462950 OR
30584782 OR 23504137 OR 24390808 OR 27531738 OR 26251693 OR 27562357 OR 27887610 OR
25711654 OR 24997174 OR 29526243 OR 28410164 OR 28900822 OR 27254272 OR 24065550 OR
30229557 OR 27333128 OR 29388687 OR 24335923 OR 31520215 OR 22710259 OR 23604998 OR
25833659 OR 25545411 OR 28258052 OR 25338995 OR 24870782 OR 29273954 OR 32521652 OR
31642349 OR 29593021 OR 25073541 OR 28935620 OR 28561685 OR 30256657 OR 28410162 OR
23054109 OR 26284121 OR 24389524 OR 27793125 OR 27863058 OR 32558129 OR 28427084 OR
22544562 OR 32379234 OR 29043460 OR 26602701 OR 24845177 OR 27061740 OR 25060288 OR
31012968 OR 30766696 OR 28845551 OR 31605514 OR 31377976 OR 24123482 OR 29390904 OR
24569944 OR 28444534 OR 25792854 OR 31745553 OR 29698983 OR 27832718 OR 31187251 OR
28288595 OR 31773148 OR 25367403 OR 31165653 OR 31217021 OR 31237570 OR 30180192 OR
26915025 OR 26059936 OR 26896305 OR 27037811 OR 23940231 OR 23947581 OR 24739260 OR
24317968 OR 31970652 OR 25088804 OR 23439658 OR 29553651 OR 27529826 OR 29944706 OR
31389108 OR 28756991 OR 23564803 OR 28856470 OR 25304986 OR 31561728 OR 24578086 OR
23514347 OR 29330081 OR 28166765 OR 25784579 OR 23242613 OR 32414347 OR 27612561 OR
30402781 OR 32059728 OR 29402761 OR 28097626 OR 26110777 OR 29637645 OR 31269917 OR
25110844 OR 28766098 OR 28247126 OR 23402226 OR 30272836 OR 30244781 OR 23456557 OR
30361831 OR 26026737 OR 28538261 OR 23179496 OR 32240461 OR 31046885 OR 26051073 OR
26923090 OR 27232866 OR 32193692 OR 25975675 OR 29881639 OR 29252103 OR 25752971 OR
27857248 OR 32546796 OR 23443319 OR 27821934 OR 27368882 OR 29579014 OR 27668296 OR
32385847 OR 28410174 OR 23505170 OR 30138021 OR 29770553 OR 24105359 OR 29789774 OR

27884071 OR 26606746 OR 24834485 OR 24882370 OR 24131016 OR 30794107 OR 25832894 OR 31694687 OR 23829442 OR 32103358 OR 23663078 OR 28553684 OR 31367917 OR 26829081 OR 30917793 OR 32407010 OR 24013569 OR 31752830 OR 28602712 OR 32395379 OR 23515464 OR 27409075 OR 30021554 OR 30202391 OR 30129907 OR 27464488 OR 27252076 OR 24486636 OR 29501739 OR 29344401 OR 31996235 OR 23041586 OR 25427610 OR 30128855 OR 32009481 OR 27543065 OR 26932848 OR 23888337 OR 24596360 OR 26518022 OR 29740782 OR 27433356 OR 24911404 OR 26988367 OR 28698390 OR 31012970 OR 30312118 OR 28926676 OR 23918953 OR 25124456 OR 26887585 OR 30062572 OR 24708832 OR 25452437 OR 32090192 OR 30071146 OR 27129840 OR 32147571 OR 30247961 OR 26598750 OR 28627254 OR 27187092 OR 27629548 OR 32406224 OR 29188593 OR 26706665 OR 29688834 OR 23266440 OR 27909546 OR 23488617 OR 29319387 OR 32307459 OR 30826763 OR 31912361 OR 31712954 OR 30638093 OR 32415386 OR 26715294 OR 26593858 OR 29683152 OR 32326769 OR 28411330 OR 26282657 OR 31033073 OR 25417947 OR 31773112 OR 29187408 OR 26059937 OR 31003402 OR 29395306 OR 24894838 OR 30783858 OR 29474729 OR 28084890 OR 28634625 OR 28838886 OR 27752993 OR 22451113 OR 26202538 OR 32298326 OR 23907996 OR 27785639 OR 30775929 OR 25751587 OR 28881471 OR 31076901 OR 29097298 OR 27881518 OR 27455846 OR 25563360 OR 27100859 OR 31860135 OR 26362355 OR 30077954 OR 30659130 OR 31041830 OR 29222705 OR 28489508 OR 25793000 OR 24254037 OR 25749595 OR 25555831 OR 32314053 OR 29325658 OR 23799886 OR 27332968 OR 25757733 OR 26973913 OR 26960972 OR 31272399 OR 24192320 OR 31275735 OR 30415752 OR 32344683 OR 32384481 OR 27858198 OR 28470507 OR 26777589 OR 25526904 OR 27976933 OR 23793468 OR 28012121 OR 24829866 OR 23969632 OR 25186211 OR 29162619 OR 25338320 OR 30539102 OR 27766453 OR 31933148 OR 27226430 OR 28741202 OR 31780003 OR 27293508 OR 30060250 OR 28444532 OR 27868156 OR 27557405 OR 31395070 OR 26714371 OR 22864470 OR 23370582 OR 29129704 OR 27865259 OR 24856854 OR 25935584 OR 25964257 OR 26605003 OR 24987274 OR 24891269 OR 31099634 OR 29478430 OR 32441691 OR 29952241 OR 28739540 OR 28242595 OR 28803675 OR 25739642 OR 25336068 OR 30933317 OR 24703167 OR 24470004

March 2023 Updated search with items from previous search removed

(((("Breast Cancer Lymphedema"[mesh:noexp] OR "Breast Neoplasms"[mesh:noexp] OR "Carcinoma, Ductal, Breast"[mesh:noexp] OR "Hereditary Breast and Ovarian Cancer Syndrome"[Mesh] OR "Triple Negative Breast Neoplasms"[Mesh:noexp]) OR ((breast[tiab] AND (cancer*[tiab] OR carcinoma*[tiab] OR neoplasm*[tiab] OR tumor*[tiab] OR tumour*[tiab])))) AND (("cancer survivors"[mesh:noexp] OR survivors[mesh:noexp]) OR (survivor*[tiab]))) AND (("Circuit-Based Exercise"[mesh:noexp] OR "Dance Therapy"[mesh:noexp] OR "Endurance Training"[mesh:noexp] OR "Exercise"[mesh:noexp] OR "Exercise Therapy"[Mesh:NoExp] OR "Exercise Tolerance"[mesh:noexp] OR "Gymnastics"[mesh:noexp] OR "High-Intensity Interval Training"[mesh:noexp] OR "Jogging"[mesh:noexp] OR "Motion Therapy, Continuous Passive"[Mesh:NoExp] OR "Motor Activity"[mesh:noexp] OR "Muscle Stretching Exercises"[mesh:noexp] OR "Physical Conditioning, Human"[mesh:noexp] OR "Physical Endurance"[mesh:noexp] OR "Plyometric Exercise"[mesh:noexp] OR "Qigong"[mesh:noexp] OR "Resistance Training"[mesh:noexp] OR "Running"[mesh:noexp] OR "Stair Climbing"[mesh:noexp] OR "Swimming"[mesh:noexp] OR "Tai Ji"[mesh:noexp] OR "Walking"[mesh:noexp] OR "Yoga"[mesh:noexp]) OR (aerobics[tiab] OR ballet[tiab] OR bicycling[tiab] OR biking[tiab] OR calisthenics[tiab] OR "circuit training"[tiab] OR dance[tiab] OR exercise[tiab] OR physical activit*[tiab] OR "Physical Endurance"[tiab] OR "Plyometric*"[tiab] OR running[tiab] OR swimming[tiab] OR "tai chi"[tiab] OR "tai ji"[tiab] OR walking[tiab] OR yoga[tiab] OR ((strength[tiab] OR resistance[tiab]) AND training[tiab])))) AND (("Clinical Trial" [PTYP:NoExp] OR "Adaptive Clinical Trial" [PTYP:NoExp] OR "clinical trial, phase ii"[PTYP] OR "clinical trial, phase iii"[PTYP] OR "clinical trial, phase iv"[PTYP] OR "controlled clinical trial"[PTYP] OR "multicenter study"[PTYP] OR "randomized controlled trial"[PTYP] OR "Clinical Trials as Topic"[mesh:noexp] OR "clinical trials, phase ii as topic"[MeSH:noexp] OR "clinical trials, phase iii as topic"[MeSH:noexp] OR "clinical trials, phase iv as topic"[MeSH:noexp] OR "controlled clinical trials as topic"[MeSH:noexp] OR "Non-Randomized Controlled Trials as Topic"[MeSH:noexp] OR "randomized controlled trials as topic"[MeSH:noexp] OR "Equivalence Trials as Topic"[MeSH:noexp] OR "Intention to Treat Analysis"[MeSH:noexp] OR "Pragmatic Clinical Trials as Topic"[MeSH:noexp] OR "early termination of clinical trials"[MeSH:noexp] OR "multicenter studies

as topic"[MeSH:noexp] OR “Double-Blind Method”[Mesh] OR "phase II"[tiab] OR "phase III"[tiab] OR "phase IV"[tiab] OR "phase 2"[tiab] OR "phase 3"[tiab] OR "phase 4"[tiab] OR ((randomised[TIAB] OR randomized[TIAB]) AND (trial[TIAB] OR trials[tiab])) OR ((single[TIAB] OR double[TIAB] OR doubled[TIAB] OR triple[TIAB] OR tripled[TIAB] OR treble[TIAB] OR treble[TIAB]) AND (blind*[TIAB] OR mask*[TIAB])) OR ("4 arm"[tiab] OR "four arm"[tiab])) OR (evaluation study[PT:NOEXP] OR "evaluation studies as topic"[MESH:NOEXP] OR "program evaluation"[MESH:NOEXP] OR "validation study"[PT:NOEXP] OR "validation studies as topic"[MESH:NOEXP] OR (pre-[TIAB] AND post-[TIAB]) OR (pretest[TIAB] AND posttest[TIAB]) OR (program*[TIAB] AND (evaluate[TIAB] OR evaluated[TIAB] OR evaluates[TIAB] OR evaluating[TIAB] OR evaluation[TIAB] OR evaluations[TIAB] OR evaluator[TIAB] OR evaluators[TIAB])) OR effectiveness[TIAB] OR intervention[TIAB])) AND English[la] AND 2013:3000[dp] NOT (22451113 OR 22535636 OR 22544562 OR 22573338 OR 22672363 OR 22710259 OR 22822181 OR 22831916 OR 22864470 OR 22879576 OR 22996743 OR 23017985 OR 23026681 OR 23041586 OR 23054109 OR 23177321 OR 23179496 OR 23184120 OR 23213072 OR 23242613 OR 23244677 OR 23266440 OR 23274615 OR 23293010 OR 23326466 OR 23370582 OR 23378138 OR 23402226 OR 23430010 OR 23439424 OR 23439658 OR 23443319 OR 23456557 OR 23488617 OR 23504137 OR 23505170 OR 23514347 OR 23515464 OR 23519041 OR 23529000 OR 23538987 OR 23564803 OR 23575310 OR 23579251 OR 23604998 OR 23612029 OR 23615146 OR 23620504 OR 23620675 OR 23635341 OR 23663078 OR 23749481 OR 23766391 OR 23793468 OR 23799886 OR 23829442 OR 23860950 OR 23876573 OR 23888337 OR 23906102 OR 23907996 OR 23915045 OR 23918953 OR 23940231 OR 23947581 OR 23963636 OR 23969632 OR 23989030 OR 24013569 OR 24043292 OR 24065550 OR 24105359 OR 24123482 OR 24131016 OR 24176784 OR 24192320 OR 24212124 OR 24222132 OR 24254037 OR 24310809 OR 24317968 OR 24319454 OR 24335923 OR 24337598 OR 24389524 OR 24390808 OR 24419734 OR 24470004 OR 24486636 OR 24495696 OR 24569944 OR 24578086 OR 24596359 OR 24596360 OR 24633590 OR 24644304 OR 24648018 OR 24703167 OR 24707505 OR 24708832 OR 24739260 OR 24804802 OR 24805910 OR 24829866 OR 24834485 OR 24845177 OR 24856854 OR 24869977 OR 24870782 OR 24882370 OR 24891269 OR 24894145 OR 24894838 OR 24911404 OR 24916951 OR 24987274 OR 24997174 OR 25037667 OR 25060288 OR 25073541 OR 25088804 OR 25110844 OR 25124456 OR 25132146 OR 25148839 OR 25171662 OR 25179578 OR 25181937 OR 25186211 OR 25304986 OR 25313756 OR 25336068 OR 25337546 OR 25338320 OR 25338995 OR 25349070 OR 25367403 OR 25394834 OR 25417174 OR 25417947 OR 25427610 OR 25452437 OR 25526904 OR 25545411 OR 25555831 OR 25563360 OR 25567329 OR 25576214 OR 25602030 OR 25614924 OR 25645134 OR 25672583 OR 25681782 OR 25708515 OR 25711654 OR 25711667 OR 25724409 OR 25730596 OR 25739642 OR 25749595 OR 25749601 OR 25751587 OR 25752971 OR 25757733 OR 25781872 OR 25784579 OR 25792854 OR 25793000 OR 25814054 OR 25832894 OR 25833659 OR 25833781 OR 25845088 OR 25853030 OR 25899303 OR 25901376 OR 25903195 OR 25920528 OR 25935584 OR 25964257 OR 25965782 OR 25975457 OR 25975675 OR 25980790 OR 26000593 OR 26016834 OR 26026737 OR 26051073 OR 26059936 OR 26059937 OR 26068412 OR 26110777 OR 26175059 OR 26199085 OR 26202538 OR 26251693 OR 26282657 OR 26284121 OR 26286486 OR 26303657 OR 26304504 OR 26315617 OR 26327866 OR 26362355 OR 26373696 OR 26395825 OR 26427563 OR 26512712 OR 26518022 OR 26543382 OR 26543789 OR 26586495 OR 26593858 OR 26598750 OR 26602701 OR 26605003 OR 26606746 OR 26640827 OR 26655430 OR 26677207 OR 26679449 OR 26703650 OR 26706665 OR 26713501 OR 26714371 OR 26715294 OR 26756114 OR 26769117 OR 26777589 OR 26782031 OR 26801931 OR 26820653 OR 26829081 OR 26840439 OR 26850265 OR 26855719 OR 26869680 OR 26880252 OR 26887585 OR 26896305 OR 26915025 OR 26923090 OR 26925999 OR 26932848 OR 26935995 OR 26945570 OR 26960972 OR 26970957 OR 26973913 OR 26984227 OR 26988367 OR 27037811 OR 27061740 OR 27100859 OR 27110131 OR 27129840 OR 27146839 OR 27161493 OR 27187092 OR 27192633 OR 27197276 OR 27226430 OR 27232866 OR 27252076 OR 27254272 OR 27258052 OR 27293508 OR 27295811 OR 27312845 OR 27332968 OR 27333128 OR 27334210 OR 27368882 OR 27409075 OR 27416835 OR 27417791 OR 27433356 OR 27455846 OR 27464488 OR 27470258 OR 27490111 OR 27498099 OR 27516182 OR 27524376 OR 27529826 OR 27530961 OR 27531024 OR 27531738 OR 27539586 OR 27543047 OR 27543065 OR 27557405 OR 27562357 OR 27565830 OR 27601139 OR 27612561 OR 27629548 OR 27635602 OR 27646264 OR

27668296 OR 27718533 OR 27737976 OR 27738306 OR 27742668 OR 27752993 OR 27766453 OR 27785639 OR 27793125 OR 27821934 OR 27821958 OR 27832718 OR 27838922 OR 27840340 OR 27857248 OR 27857276 OR 27858198 OR 27863058 OR 27865259 OR 27868156 OR 27873046 OR 27881518 OR 27883168 OR 27884071 OR 27887610 OR 27893212 OR 27893938 OR 27909546 OR 27912947 OR 27925359 OR 27976933 OR 27981118 OR 27988868 OR 28012121 OR 28026901 OR 28070770 OR 28075038 OR 28084890 OR 28097626 OR 28150044 OR 28161788 OR 28166765 OR 28178355 OR 28189100 OR 28228661 OR 28242595 OR 28247126 OR 28253845 OR 28258052 OR 28288595 OR 28347291 OR 28392934 OR 28410162 OR 28410164 OR 28410171 OR 28410174 OR 28411330 OR 28414894 OR 28417203 OR 28427084 OR 28427925 OR 28444532 OR 28444534 OR 28470368 OR 28470507 OR 28489508 OR 28525464 OR 28538261 OR 28551083 OR 28553684 OR 28561685 OR 28588911 OR 28602712 OR 28620700 OR 28624715 OR 28624949 OR 28627254 OR 28632240 OR 28634625 OR 28636526 OR 28639097 OR 28639157 OR 28665541 OR 28666465 OR 28698390 OR 28702218 OR 28717378 OR 28721384 OR 28736768 OR 28739540 OR 28741202 OR 28745131 OR 28756991 OR 28766098 OR 28803675 OR 28836379 OR 28838886 OR 28845551 OR 28856470 OR 28881471 OR 28894513 OR 28900822 OR 28922074 OR 28926676 OR 28935620 OR 28965138 OR 28991039 OR 29026725 OR 29043460 OR 29057279 OR 29076388 OR 29082588 OR 29086523 OR 29097298 OR 29129704 OR 29141853 OR 29162619 OR 29168064 OR 29187408 OR 29188593 OR 29222705 OR 29243165 OR 29252103 OR 29259819 OR 29260391 OR 29273954 OR 29319387 OR 29325658 OR 29330081 OR 29344401 OR 29356607 OR 29388687 OR 29390904 OR 29395306 OR 29402761 OR 29404842 OR 29409128 OR 29409922 OR 29470705 OR 29470804 OR 29471477 OR 29474729 OR 29478430 OR 29498250 OR 29501739 OR 29511965 OR 29526243 OR 29553651 OR 29579014 OR 29587682 OR 29593021 OR 29617707 OR 29637645 OR 29644889 OR 29645327 OR 29654416 OR 29683152 OR 29688834 OR 29698983 OR 29740782 OR 29746264 OR 29770553 OR 29789774 OR 29847241 OR 29869823 OR 29880779 OR 29881639 OR 29932460 OR 29936464 OR 29943097 OR 29944706 OR 29952241 OR 29961039 OR 30021554 OR 30060250 OR 30062572 OR 30067063 OR 30071146 OR 30077574 OR 30077954 OR 30117760 OR 30128855 OR 30129907 OR 30138021 OR 30180192 OR 30202391 OR 30229557 OR 30233121 OR 30244781 OR 30247961 OR 30256657 OR 30272836 OR 30312118 OR 30320304 OR 30339481 OR 30340503 OR 30361831 OR 30368741 OR 30376366 OR 30400784 OR 30402781 OR 30415752 OR 30445595 OR 30485297 OR 30488344 OR 30500981 OR 30539102 OR 30543479 OR 30554276 OR 30554923 OR 30562305 OR 30584782 OR 30609982 OR 30638093 OR 30658629 OR 30659130 OR 30663156 OR 30694978 OR 30723698 OR 30766696 OR 30767964 OR 30775929 OR 30783858 OR 30789349 OR 30794107 OR 30826763 OR 30840336 OR 30850143 OR 30862228 OR 30864301 OR 30874369 OR 30874927 OR 30912010 OR 30912011 OR 30917793 OR 30933317 OR 30972643 OR 30980431 OR 30982113 OR 31003402 OR 31012968 OR 31012970 OR 31014267 OR 31028212 OR 31033073 OR 31041685 OR 31041830 OR 31046885 OR 31076901 OR 31087419 OR 31099634 OR 31121916 OR 31144265 OR 31165653 OR 31168711 OR 31173341 OR 31187251 OR 31187411 OR 31206630 OR 31217021 OR 31227537 OR 31236699 OR 31237570 OR 31244536 OR 31264183 OR 31269917 OR 31272399 OR 31275735 OR 31290395 OR 31309977 OR 31319398 OR 31354000 OR 31367917 OR 31377976 OR 31389108 OR 31395070 OR 31415063 OR 31444192 OR 31454519 OR 31475306 OR 31520215 OR 31561728 OR 31600424 OR 31604709 OR 31605312 OR 31605514 OR 31617073 OR 31617564 OR 31642349 OR 31689771 OR 31694687 OR 31712954 OR 31745553 OR 31752830 OR 31759342 OR 31763746 OR 31773112 OR 31773148 OR 31780003 OR 31791246 OR 31804383 OR 31853701 OR 31860135 OR 31912361 OR 31933148 OR 31964669 OR 31970652 OR 31994181 OR 31996235 OR 31998651 OR 32009481 OR 32019093 OR 32020356 OR 32020706 OR 32031221 OR 32059728 OR 32071117 OR 32090192 OR 32103358 OR 32111767 OR 32112354 OR 32130627 OR 32147571 OR 32193692 OR 32232722 OR 32234652 OR 32240461 OR 32249355 OR 32298326 OR 32298407 OR 32304828 OR 32306100 OR 32307459 OR 32307828 OR 32314053 OR 32314110 OR 32326769 OR 32344683 OR 32348688 OR 32379234 OR 32384481 OR 32385847 OR 32395379 OR 32404725 OR 32406224 OR 32407010 OR 32414347 OR 32415386 OR 32415529 OR 32441691 OR 32443355 OR 32462950 OR 32510461 OR 32521652 OR 32535764 OR 32546796 OR 32558129 OR 32599641 OR 32605075 OR 32607599 OR 32616883 OR 32631302 OR 32664375 OR 32664946 OR 32673270 OR 32673271 OR 32678375 OR 32681303 OR 32694366 OR 32698957 OR 32699997 OR 32754489 OR 32763011 OR 32803384 OR 32808383 OR 32819304 OR 32859615 OR 32868938 OR

32875536 OR 32877271 OR 32883829 OR 32885221 OR 32886189 OR 32906761 OR 32924562 OR 32930924 OR 32940768 OR 32955446 OR 32960338 OR 33010183 OR 33025451 OR 33025453 OR 33036706 OR 33059741 OR 33064204 OR 33072603 OR 33076863 OR 33089371 OR 33105793 OR 33109503 OR 33123481 OR 33128705 OR 33163551 OR 33172957 OR 33178447 OR 33194622 OR 33212425 OR 33218558 OR 33225208 OR 33289669 OR 33292676 OR 33305350 OR 33312762 OR 33312937 OR 33396551 OR 33403402 OR 33423594 OR 33452652 OR 33467265 OR 33473331 OR 33475511 OR 33482396 OR 33491338 OR 33503496 OR 33539554 OR 33550878 OR 33564742 OR 33570477 OR 33588948 OR 33613314 OR 33618699 OR 33624172 OR 33677463 OR 33677781 OR 33689150 OR 33689553 OR 33709302 OR 33737299 OR 33752433 OR 33754246 OR 33786507 OR 33787643 OR 33801189 OR 33807611 OR 33811517 OR 33815828 OR 33823832 OR 33840972 OR 33847588 OR 33849087 OR 33883475 OR 33890857 OR 33896706 OR 33912450 OR 33929533 OR 33935799 OR 33950477 OR 33954891 OR 33956212 OR 33961667 OR 33976224 OR 33977342 OR 33978132 OR 33983599 OR 33989021 OR 33992279 OR 34011028 OR 34049837 OR 34066752 OR 34089422 OR 34125072 OR 34130213 OR 34135964 OR 34146655 OR 34147107 OR 34160294 OR 34161602 OR 34189070 OR 34204528 OR 34207765 OR 34213086 OR 34225091 OR 34258817 OR 34274495 OR 34281126 OR 34290337 OR 34305108 OR 34311713 OR 34313858 OR 34314878 OR 34328623 OR 34344171 OR 34355298 OR 34357555 OR 34359567 OR 34365016 OR 34389552 OR 34413391 OR 34426142 OR 34427806 OR 34436552 OR 34444332 OR 34456250 OR 34461975 OR 34463835 OR 34465518 OR 34467791 OR 34480935 OR 34491527 OR 34498828 OR 34503198 OR 34524631 OR 34546611 OR 34554567 OR 34559724 OR 34567220 OR 34578787 OR 34578984 OR 34579142 OR 34586509 OR 34589720 OR 34590926 OR 34593453 OR 34598902 OR 34609311 OR 34638355 OR 34639353 OR 34649267 OR 34651554 OR 34664019 OR 34674806 OR 34677269 OR 34684403 OR 34712520 OR 34721081 OR 34729835 OR 34733065 OR 34741653 OR 34777343 OR 34778552 OR 34789309 OR 34812521 OR 34816921 OR 34823494 OR 34836345 OR 34887491 OR 34898001 OR 34922621 OR 34989962 OR 34994945 OR 35012011 OR 35022884 OR 35025064 OR 35040076 OR 35045736 OR 35057525 OR 35087367 OR 35091272 OR 35107399 OR 35109799 OR 35110796 OR 35112212 OR 35130491 OR 35140880 OR 35156143 OR 35171707 OR 35182789 OR 35191900 OR 35200150 OR 35207294 OR 35244811 OR 35246219 OR 35253020 OR 35253682 OR 35257993 OR 35265005 OR 35277360 OR 35278203 OR 35289219 OR 35289926 OR 35314958 OR 35326679 OR 35328964 OR 35331230 OR 35342756 OR 35347550 OR 35363152 OR 35363502 OR 35366505 OR 35391574 OR 35398558 OR 35401377 OR 35416341 OR 35428285 OR 35431214 OR 35441995 OR 35455864 OR 35460441 OR 35471614 OR 35477489 OR 35477651 OR 35486425 OR 35488902 OR 35509876)
**October 2025 updated search**<br>**Note:** Trials filter was updated
(("Breast Cancer Lymphedema"[mesh:noexp] OR "Breast Neoplasms"[mesh:noexp] OR "Carcinoma, Ductal, Breast"[mesh:noexp] OR "Hereditary Breast AND Ovarian Cancer Syndrome"[Mesh] OR "Triple Negative Breast Neoplasms"[Mesh:noexp]) OR (breast[tiab] AND (cancer*[tiab] OR carcinoma*[tiab] OR neoplasm*[tiab] OR tumor*[tiab] OR tumour*[tiab]))) AND ("cancer survivors"[mesh:noexp] OR survivors[mesh:noexp] OR survivor*[tiab]) AND ("Circuit-Based Exercise"[mesh:noexp] OR "Dance Therapy"[mesh:noexp] OR "Endurance Training"[mesh:noexp] OR "Exercise"[mesh:noexp] OR "Exercise Therapy"[Mesh:NoExp] OR "Exercise Tolerance"[mesh:noexp] OR "Gymnastics"[mesh:noexp] OR "High-Intensity Interval Training"[mesh:noexp] OR "Jogging"[mesh:noexp] OR "Motion Therapy, Continuous Passive"[Mesh:NoExp] OR "Motor Activity"[mesh:noexp] OR "Muscle Stretching Exercises"[mesh:noexp] OR "Physical Conditioning, Human"[mesh:noexp] OR "Physical Endurance"[mesh:noexp] OR "Plyometric Exercise"[mesh:noexp] OR "Qigong"[mesh:noexp] OR "Resistance Training"[mesh:noexp] OR "Running"[mesh:noexp] OR "Stair Climbing"[mesh:noexp] OR "Swimming"[mesh:noexp] OR "Tai Ji"[mesh:noexp] OR "Walking"[mesh:noexp] OR "Yoga"[mesh:noexp] OR aerobics[tiab] OR ballet[tiab] OR bicycling[tiab] OR biking[tiab] OR calisthenics[tiab] OR "circuit training"[tiab] OR dance[tiab] OR exercise[tiab] OR physical activit*[tiab] OR "Physical Endurance"[tiab] OR "Plyometric*"[tiab] OR running[tiab] OR swimming[tiab] OR "tai chi"[tiab] OR "tai ji"[tiab] OR walking[tiab] OR yoga[tiab] OR ((strength[tiab] OR resistance[tiab]) AND training[tiab])) AND ("Adaptive Clinical Trial" [PTYP:NoExp] OR "Clinical Trial" [PTYP:NoExp] OR "clinical trial, phase i"[PTYP] OR "clinical

trial, phase ii"[PTYP] OR "clinical trial, phase iii"[PTYP] OR "clinical trial, phase iv"[PTYP] OR "controlled clinical trial"[PTYP] OR "Equivalence Trial"[PTYP] OR "multicenter study"[PTYP] OR "pragmatic clinical trial"[PTYP] OR "randomized controlled trial"[PTYP] OR "adaptive clinical trials as topic"[MeSH:noexp] OR "Clinical Studies as Topic"[mesh:noexp] OR "Clinical Trials as Topic"[mesh:noexp] OR "clinical trials, phase i as topic"[MeSH:noexp] OR "clinical trials, phase ii as topic"[MeSH:noexp] OR "clinical trials, phase iii as topic"[MeSH:noexp] OR "clinical trials, phase iv as topic"[MeSH:noexp] OR "controlled clinical trials as topic"[MeSH:noexp] OR "Double-Blind Method"[Mesh] OR "early termination of clinical trials"[MeSH:noexp] OR "Equivalence Trials as Topic"[MeSH:noexp] OR "Intention to Treat Analysis"[MeSH:noexp] OR "multicenter studies as topic"[MeSH:noexp] OR "Non-Randomized Controlled Trials as Topic"[MeSH:noexp] OR "Pragmatic Clinical Trials as Topic"[MeSH:noexp] OR "randomized controlled trials as topic"[MeSH:noexp] OR "2 arm"[TIAB] OR "two arm"[TIAB] OR "3 arm"[TIAB] OR "three arm"[TIAB] OR "4 arm"[TIAB] OR "four arm"[TIAB] OR "clinical studies"[TIAB:~3] OR "clinical study"[TIAB:~3] OR "clinical trial"[TIAB:~3] OR "clinical trials"[TIAB:~3] OR "controlled studies"[TIAB:~3] OR "controlled study"[TIAB:~3] OR "controlled trial"[TIAB:~3] OR "controlled trials"[TIAB:~3] OR ("cross-over"[TIAB] AND "over trial"[TIAB:~3]) OR ("cross-over"[TIAB] AND "over trials"[TIAB:~3]) OR "crossover trial"[TIAB:~3] OR "crossover trials"[TIAB:~3] OR "equivalent study"[TIAB:~3] OR "equivalent trial"[TIAB:~3] OR "equivalent trials"[TIAB:~3] OR intervention[TI] OR "pragmatic study"[TIAB:~3] OR "pragmatic trial"[TIAB:~3] OR "pragmatic trials"[TIAB:~3] OR "randomised cross"[TIAB] OR "randomised crossover"[TIAB] OR "randomized cross"[TIAB:~3] OR "randomized crossover"[TIAB:~3] OR "randomised studies"[TIAB:~3] OR "randomised study"[TIAB:~3] OR "randomised trial"[TIAB:~3] OR "randomised trials"[TIAB:~3] OR "randomized studies"[TIAB:~3] OR "randomized study"[TIAB:~3] OR "randomized trial"[TIAB:~3] OR "randomized trials"[TIAB:~3] OR "stepped wedge"[TIAB] OR ("window opportunity"[tiab:~1] AND trial[tiab]) OR "phase I"[TIAB] OR "phase II"[TIAB] OR "phase III"[TIAB] OR "phase IV"[TIAB] OR "phase 1"[TIAB] OR "phase 2"[TIAB] OR "phase 3"[TIAB] OR "phase 4"[TIAB] OR "single blind"[TIAB] OR "single blinded"[TIAB] OR "single mask"[TIAB] OR "single masked"[TIAB] OR "double blind"[TIAB] OR "double blinded"[TIAB] OR "double mask"[TIAB] OR "double masked"[TIAB] OR "triple blind"[TIAB] OR "triple blinded"[TIAB] OR "triple masked"[TIAB] OR "tripled blinded"[TIAB] OR (("comparative study"[PT:NOEXP] OR comparative[TI] OR "comparative analysis"[TIAB:~2] OR "comparative outcome"[TIAB] OR "comparative outcomes"[TIAB] OR "comparative study"[TIAB:~4] OR "compared to"[TIAB] OR "compared with"[TIAB] OR comparing[TI] OR "comparison between"[TI] OR "comparison of"[TI] OR versus[ti] OR vs[ti] OR "superiority analysis"[TIAB:~2]) AND (controlled[TIAB] OR inferiority[TIAB] OR randomised[TIAB] OR randomized[TIAB] OR trial[TI] OR trials[TI]))) AND English[la] AND 2013:3000[dp] NOT (22451113 OR 22535636 OR 22544562 OR 22573338 OR 22672363 OR 22710259 OR 22822181 OR 22831916 OR 22864470 OR 22879576 OR 22996743 OR 23017985 OR 23026681 OR 23041586 OR 23054109 OR 23177321 OR 23179496 OR 23184120 OR 23213072 OR 23242613 OR 23244677 OR 23266440 OR 23274615 OR 23293010 OR 23326466 OR 23370582 OR 23378138 OR 23402226 OR 23430010 OR 23439424 OR 23439658 OR 23443319 OR 23456557 OR 23488617 OR 23504137 OR 23505170 OR 23514347 OR 23515464 OR 23519041 OR 23529000 OR 23538987 OR 23564803 OR 23575310 OR 23579251 OR 23604998 OR 23612029 OR 23615146 OR 23620504 OR 23620675 OR 23635341 OR 23663078 OR 23749481 OR 23766391 OR 23793468 OR 23799886 OR 23829442 OR 23860950 OR 23876573 OR 23888337 OR 23906102 OR 23907996 OR 23915045 OR 23918953 OR 23940231 OR 23947581 OR 23963636 OR 23969632 OR 23989030 OR 24013569 OR 24043292 OR 24065550 OR 24105359 OR 24123482 OR 24131016 OR 24176784 OR 24192320 OR 24212124 OR 24222132 OR 24254037 OR 24310809 OR 24317968 OR 24319454 OR 24335923 OR 24337598 OR 24389524 OR 24390808 OR 24419734 OR 24470004 OR 24486636 OR 24495696 OR 24569944 OR 24578086 OR 24596359 OR 24596360 OR 24633590 OR 24644304 OR 24648018 OR 24703167 OR 24707505 OR 24708832 OR 24739260 OR 24804802 OR 24805910 OR 24829866 OR 24834485 OR 24845177 OR 24856854 OR 24869977 OR 24870782 OR 24882370 OR 24891269 OR 24894145 OR 24894838 OR 24911404 OR 24916951 OR 24987274 OR 24997174 OR 25037667 OR 25060288 OR 25073541 OR 25088804 OR 25110844 OR 25124456 OR 25132146 OR 25148839 OR 25171662 OR 25179578 OR 25181937 OR 25186211 OR 25304986 OR 25313756 OR 25336068 OR 25337546 OR

25338320 OR 25338995 OR 25349070 OR 25367403 OR 25394834 OR 25417174 OR 25417947 OR 25427610 OR 25452437 OR 25526904 OR 25545411 OR 25555831 OR 25563360 OR 25567329 OR 25576214 OR 25602030 OR 25614924 OR 25645134 OR 25672583 OR 25681782 OR 25708515 OR 25711654 OR 25711667 OR 25724409 OR 25730596 OR 25739642 OR 25749595 OR 25749601 OR 25751587 OR 25752971 OR 25757733 OR 25781872 OR 25784579 OR 25792854 OR 25793000 OR 25814054 OR 25832894 OR 25833659 OR 25833781 OR 25845088 OR 25853030 OR 25899303 OR 25901376 OR 25903195 OR 25920528 OR 25935584 OR 25964257 OR 25965782 OR 25975457 OR 25975675 OR 25980790 OR 26000593 OR 26016834 OR 26026737 OR 26051073 OR 26059936 OR 26059937 OR 26068412 OR 26110777 OR 26175059 OR 26199085 OR 26202538 OR 26251693 OR 26282657 OR 26284121 OR 26286486 OR 26303657 OR 26304504 OR 26315617 OR 26327866 OR 26362355 OR 26373696 OR 26395825 OR 26427563 OR 26512712 OR 26518022 OR 26543382 OR 26543789 OR 26586495 OR 26593858 OR 26598750 OR 26602701 OR 26605003 OR 26606746 OR 26640827 OR 26655430 OR 26677207 OR 26679449 OR 26703650 OR 26706665 OR 26713501 OR 26714371 OR 26715294 OR 26756114 OR 26769117 OR 26777589 OR 26782031 OR 26801931 OR 26820653 OR 26829081 OR 26840439 OR 26850265 OR 26855719 OR 26869680 OR 26880252 OR 26887585 OR 26896305 OR 26915025 OR 26923090 OR 26925999 OR 26932848 OR 26935995 OR 26945570 OR 26960972 OR 26970957 OR 26973913 OR 26984227 OR 26988367 OR 27037811 OR 27061740 OR 27100859 OR 27110131 OR 27129840 OR 27146839 OR 27161493 OR 27187092 OR 27192633 OR 27197276 OR 27226430 OR 27232866 OR 27252076 OR 27254272 OR 27258052 OR 27293508 OR 27295811 OR 27312845 OR 27332968 OR 27333128 OR 27334210 OR 27368882 OR 27409075 OR 27416835 OR 27417791 OR 27433356 OR 27455846 OR 27464488 OR 27470258 OR 27490111 OR 27498099 OR 27516182 OR 27524376 OR 27529826 OR 27530961 OR 27531024 OR 27531738 OR 27539586 OR 27543047 OR 27543065 OR 27557405 OR 27562357 OR 27565830 OR 27601139 OR 27612561 OR 27629548 OR 27635602 OR 27646264 OR 27668296 OR 27718533 OR 27737976 OR 27738306 OR 27742668 OR 27752993 OR 27766453 OR 27785639 OR 27793125 OR 27821934 OR 27821958 OR 27832718 OR 27838922 OR 27840340 OR 27857248 OR 27857276 OR 27858198 OR 27863058 OR 27865259 OR 27868156 OR 27873046 OR 27881518 OR 27883168 OR 27884071 OR 27887610 OR 27893212 OR 27893938 OR 27909546 OR 27912947 OR 27925359 OR 27976933 OR 27981118 OR 27988868 OR 28012121 OR 28026901 OR 28070770 OR 28075038 OR 28084890 OR 28097626 OR 28150044 OR 28161788 OR 28166765 OR 28178355 OR 28189100 OR 28228661 OR 28242595 OR 28247126 OR 28253845 OR 28258052 OR 28288595 OR 28347291 OR 28392934 OR 28410162 OR 28410164 OR 28410171 OR 28410174 OR 28411330 OR 28414894 OR 28417203 OR 28427084 OR 28427925 OR 28444532 OR 28444534 OR 28470368 OR 28470507 OR 28489508 OR 28525464 OR 28538261 OR 28551083 OR 28553684 OR 28561685 OR 28588911 OR 28602712 OR 28620700 OR 28624715 OR 28624949 OR 28627254 OR 28632240 OR 28634625 OR 28636526 OR 28639097 OR 28639157 OR 28665541 OR 28666465 OR 28698390 OR 28702218 OR 28717378 OR 28721384 OR 28736768 OR 28739540 OR 28741202 OR 28745131 OR 28756991 OR 28766098 OR 28803675 OR 28836379 OR 28838886 OR 28845551 OR 28856470 OR 28881471 OR 28894513 OR 28900822 OR 28922074 OR 28926676 OR 28935620 OR 28965138 OR 28991039 OR 29026725 OR 29043460 OR 29057279 OR 29076388 OR 29082588 OR 29086523 OR 29097298 OR 29129704 OR 29141853 OR 29162619 OR 29168064 OR 29187408 OR 29188593 OR 29222705 OR 29243165 OR 29252103 OR 29259819 OR 29260391 OR 29273954 OR 29319387 OR 29325658 OR 29330081 OR 29344401 OR 29356607 OR 29388687 OR 29390904 OR 29395306 OR 29402761 OR 29404842 OR 29409128 OR 29409922 OR 29470705 OR 29470804 OR 29471477 OR 29474729 OR 29478430 OR 29498250 OR 29501739 OR 29511965 OR 29526243 OR 29553651 OR 29579014 OR 29587682 OR 29593021 OR 29617707 OR 29637645 OR 29644889 OR 29645327 OR 29654416 OR 29683152 OR 29688834 OR 29698983 OR 29740782 OR 29746264 OR 29770553 OR 29789774 OR 29847241 OR 29869823 OR 29880779 OR 29881639 OR 29932460 OR 29936464 OR 29943097 OR 29944706 OR 29952241 OR 29961039 OR 30021554 OR 30060250 OR 30062572 OR 30067063 OR 30071146 OR 30077574 OR 30077954 OR 30117760 OR 30128855 OR 30129907 OR 30138021 OR 30180192 OR 30202391 OR 30229557 OR 30233121 OR 30244781 OR 30247961 OR 30256657 OR 30272836 OR 30312118 OR 30320304 OR 30339481 OR 30340503 OR 30361831 OR 30368741 OR 30400784 OR 30402781 OR 30415752 OR 30445595 OR 30485297 OR 30488344 OR 30500981 OR 30539102 OR 30543479 OR 30554276 OR 30554923 OR 30562305 OR 30584782 OR 30609982 OR

30638093 OR 30658629 OR 30659130 OR 30663156 OR 30694978 OR 30723698 OR 30766696 OR 30767964 OR 30775929 OR 30783858 OR 30789349 OR 30794107 OR 30826763 OR 30840336 OR 30850143 OR 30862228 OR 30864301 OR 30874369 OR 30874927 OR 30912010 OR 30912011 OR 30917793 OR 30933317 OR 30972643 OR 30980431 OR 30982113 OR 31003402 OR 31012968 OR 31012970 OR 31014267 OR 31028212 OR 31033073 OR 31041685 OR 31041830 OR 31046885 OR 31076901 OR 31087419 OR 31099634 OR 31121916 OR 31144265 OR 31165653 OR 31168711 OR 31173341 OR 31187251 OR 31206630 OR 31217021 OR 31227537 OR 31236699 OR 31237570 OR 31244536 OR 31264183 OR 31269917 OR 31272399 OR 31275735 OR 31290395 OR 31309977 OR 31319398 OR 31354000 OR 31367917 OR 31377976 OR 31389108 OR 31395070 OR 31415063 OR 31444192 OR 31454519 OR 31475306 OR 31520215 OR 31561728 OR 31600424 OR 31605312 OR 31605514 OR 31617073 OR 31617564 OR 31642349 OR 31689771 OR 31694687 OR 31712954 OR 31745553 OR 31752830 OR 31759342 OR 31763746 OR 31773112 OR 31773148 OR 31780003 OR 31791246 OR 31804383 OR 31853701 OR 31860135 OR 31912361 OR 31933148 OR 31970652 OR 31994181 OR 31996235 OR 31998651 OR 32009481 OR 32019093 OR 32020356 OR 32031221 OR 32059728 OR 32090192 OR 32103358 OR 32111767 OR 32130627 OR 32147571 OR 32193692 OR 32232722 OR 32234652 OR 32240461 OR 32249355 OR 32298326 OR 32298407 OR 32304828 OR 32306100 OR 32307459 OR 32307828 OR 32314053 OR 32314110 OR 32326769 OR 32344683 OR 32348688 OR 32379234 OR 32384481 OR 32385847 OR 32395379 OR 32404725 OR 32406224 OR 32407010 OR 32414347 OR 32415386 OR 32415529 OR 32441691 OR 32443355 OR 32462950 OR 32510461 OR 32521652 OR 32535764 OR 32546796 OR 32558129 OR 35533876 OR 35578196 OR 35619910 OR 35620805 OR 35639865 OR 35641019 OR 35681702 OR 35700977 OR 35712474 OR 35731554 OR 35733142 OR 35736016 OR 35738114 OR 35749052 OR 35749369 OR 35750628 OR 35756985 OR 35771607 OR 35775225 OR 35778782 OR 35788372 OR 35795051 OR 35829982 OR 35841719 OR 35853152 OR 35861215 OR 35881067 OR 35894167 OR 35895236 OR 35896295 OR 35902321 OR 35930212 OR 35940850 OR 35952956 OR 35954479 OR 35992855 OR 35993633 OR 35994321 OR 36004842 OR 36012025 OR 36066332 OR 36076044 OR 36084228 OR 36092248 OR 36110260 OR 36141372 OR 36158576 OR 36170550 OR 36201052 OR 36207046 OR 36212795 OR 36220629 OR 36231542 OR 36238756 OR 36254717 OR 36259288 OR 36263723 OR 36269525 OR 36301400 OR 36311982 OR 36314766 OR 36336249 OR 36342348 OR 36400655 OR 36401194 OR 36403344 OR 36407802 OR 36412916 OR 36441715 OR 36451297 OR 36453589 OR 36462139 OR 36468339 OR 36474321 OR 36479658 OR 36482346 OR 36512140 OR 36512157 OR 36517097 OR 36525089 OR 36526826 OR 36535489 OR 36539160 OR 36542098 OR 36542473 OR 36550258 OR 36568235 OR 36579554 OR 36600391 OR 36612340 OR 36618335 OR 36652087 OR 36658635 OR 36674436 OR 36683179 OR 36689989 OR 36692626 OR 36694356 OR 36695128 OR 36711885 OR 36715766 OR 36723801 OR 36736725 OR 36761969 OR 36776335 OR 36777230 OR 36790946 OR 36792620 OR 36800978 OR 36814313 OR 36826074 OR 36828705 OR 36836931 OR 36843066 OR 36847633 OR 36855214 OR 36874708 OR 36890601 OR 36892780 OR 36894449 OR 36901594 OR 36904284 OR 36912039 OR 36920460 OR 36933050 OR 22451113 OR 22535636 OR 22544562 OR 22573338 OR 22672363 OR 22710259 OR 22822181 OR 22831916 OR 22864470 OR 22879576 OR 22996743 OR 23017985 OR 23026681 OR 23041586 OR 23054109 OR 23177321 OR 23179496 OR 23184120 OR 23213072 OR 23242613 OR 23244677 OR 23266440 OR 23274615 OR 23293010 OR 23326466 OR 23370582 OR 23378138 OR 23402226 OR 23430010 OR 23439424 OR 23439658 OR 23443319 OR 23456557 OR 23488617 OR 23504137 OR 23505170 OR 23514347 OR 23515464 OR 23519041 OR 23529000 OR 23538987 OR 23564803 OR 23575310 OR 23579251 OR 23604998 OR 23612029 OR 23615146 OR 23620504 OR 23620675 OR 23635341 OR 23663078 OR 23749481 OR 23766391 OR 23793468 OR 23799886 OR 23829442 OR 23860950 OR 23876573 OR 23888337 OR 23906102 OR 23907996 OR 23915045 OR 23918953 OR 23940231 OR 23947581 OR 23963636 OR 23969632 OR 23989030 OR 24013569 OR 24043292 OR 24065550 OR 24105359 OR 24123482 OR 24131016 OR 24176784 OR 24192320 OR 24212124 OR 24222132 OR 24254037 OR 24310809 OR 24317968 OR 24319454 OR 24335923 OR 24337598 OR 24389524 OR 24390808 OR 24419734 OR 24470004 OR 24486636 OR 24495696 OR 24569944 OR 24578086 OR 24596359 OR 24596360 OR 24633590 OR 24644304 OR 24648018 OR 24703167 OR 24707505 OR 24708832 OR 24739260 OR 24804802 OR 24805910 OR 24829866 OR 24834485 OR 24845177 OR 24856854 OR 24869977 OR 24870782 OR 24882370 OR 24891269 OR 24894145 OR 24894838 OR 24911404 OR 24916951 OR

24987274 OR 24997174 OR 25037667 OR 25060288 OR 25073541 OR 25088804 OR 25110844 OR 25124456 OR 25132146 OR 25148839 OR 25171662 OR 25179578 OR 25181937 OR 25186211 OR 25304986 OR 25313756 OR 25336068 OR 25337546 OR 25338320 OR 25338995 OR 25349070 OR 25367403 OR 25394834 OR 25417174 OR 25417947 OR 25427610 OR 25452437 OR 25526904 OR 25545411 OR 25555831 OR 25563360 OR 25567329 OR 25576214 OR 25602030 OR 25614924 OR 25645134 OR 25672583 OR 25681782 OR 25708515 OR 25711654 OR 25711667 OR 25724409 OR 25730596 OR 25739642 OR 25749595 OR 25749601 OR 25751587 OR 25752971 OR 25757733 OR 25781872 OR 25784579 OR 25792854 OR 25793000 OR 25814054 OR 25832894 OR 25833659 OR 25833781 OR 25845088 OR 25853030 OR 25899303 OR 25901376 OR 25903195 OR 25920528 OR 25935584 OR 25964257 OR 25965782 OR 25975457 OR 25975675 OR 25980790 OR 26000593 OR 26016834 OR 26026737 OR 26051073 OR 26059936 OR 26059937 OR 26068412 OR 26110777 OR 26175059 OR 26199085 OR 26202538 OR 26251693 OR 26282657 OR 26284121 OR 26286486 OR 26303657 OR 26304504 OR 26315617 OR 26327866 OR 26362355 OR 26373696 OR 26395825 OR 26427563 OR 26512712 OR 26518022 OR 26543382 OR 26543789 OR 26586495 OR 26593858 OR 26598750 OR 26602701 OR 26605003 OR 26606746 OR 26640827 OR 26655430 OR 26677207 OR 26679449 OR 26703650 OR 26706665 OR 26713501 OR 26714371 OR 26715294 OR 26756114 OR 26769117 OR 26777589 OR 26782031 OR 26801931 OR 26820653 OR 26829081 OR 26840439 OR 26850265 OR 26855719 OR 26869680 OR 26880252 OR 26887585 OR 26896305 OR 26915025 OR 26923090 OR 26925999 OR 26932848 OR 26935995 OR 26945570 OR 26960972 OR 26970957 OR 26973913 OR 26984227 OR 26988367 OR 27037811 OR 27061740 OR 27100859 OR 27110131 OR 27129840 OR 27146839 OR 27161493 OR 27187092 OR 27192633 OR 27197276 OR 27226430 OR 27232866 OR 27252076 OR 27254272 OR 27258052 OR 27293508 OR 27295811 OR 27312845 OR 27332968 OR 27333128 OR 27334210 OR 27368882 OR 27409075 OR 27416835 OR 27417791 OR 27433356 OR 27455846 OR 27464488 OR 27470258 OR 27490111 OR 27498099 OR 27516182 OR 27524376 OR 27529826 OR 27530961 OR 27531024 OR 27531738 OR 27539586 OR 27543047 OR 27543065 OR 27557405 OR 27562357 OR 27565830 OR 27601139 OR 27612561 OR 27629548 OR 27635602 OR 27646264 OR 27668296 OR 27718533 OR 27737976 OR 27738306 OR 27742668 OR 27752993 OR 27766453 OR 27785639 OR 27793125 OR 27821934 OR 27821958 OR 27832718 OR 27838922 OR 27840340 OR 27857248 OR 27857276 OR 27858198 OR 27863058 OR 27865259 OR 27868156 OR 27873046 OR 27881518 OR 27883168 OR 27884071 OR 27887610 OR 27893212 OR 27893938 OR 27909546 OR 27912947 OR 27925359 OR 27976933 OR 27981118 OR 27988868 OR 28012121 OR 28026901 OR 28070770 OR 28075038 OR 28084890 OR 28097626 OR 28150044 OR 28161788 OR 28166765 OR 28178355 OR 28189100 OR 28228661 OR 28242595 OR 28247126 OR 28253845 OR 28258052 OR 28288595 OR 28347291 OR 28392934 OR 28410162 OR 28410164 OR 28410171 OR 28410174 OR 28411330 OR 28414894 OR 28417203 OR 28427084 OR 28427925 OR 28444532 OR 28444534 OR 28470368 OR 28470507 OR 28489508 OR 28525464 OR 28538261 OR 28551083 OR 28553684 OR 28561685 OR 28588911 OR 28602712 OR 28620700 OR 28624715 OR 28624949 OR 28627254 OR 28632240 OR 28634625 OR 28636526 OR 28639097 OR 28639157 OR 28665541 OR 28666465 OR 28698390 OR 28702218 OR 28717378 OR 28721384 OR 28736768 OR 28739540 OR 28741202 OR 28745131 OR 28756991 OR 28766098 OR 28803675 OR 28836379 OR 28838886 OR 28845551 OR 28856470 OR 28881471 OR 28894513 OR 28900822 OR 28922074 OR 28926676 OR 28935620 OR 28965138 OR 28991039 OR 29026725 OR 29043460 OR 29057279 OR 29076388 OR 29082588 OR 29086523 OR 29097298 OR 29129704 OR 29141853 OR 29162619 OR 29168064 OR 29187408 OR 29188593 OR 29222705 OR 29243165 OR 29252103 OR 29259819 OR 29260391 OR 29273954 OR 29319387 OR 29325658 OR 29330081 OR 29344401 OR 29356607 OR 29388687 OR 29390904 OR 29395306 OR 29402761 OR 29404842 OR 29409128 OR 29409922 OR 29470705 OR 29470804 OR 29471477 OR 29474729 OR 29478430 OR 29498250 OR 29501739 OR 29511965 OR 29526243 OR 29553651 OR 29579014 OR 29587682 OR 29593021 OR 29617707 OR 29637645 OR 29644889 OR 29645327 OR 29654416 OR 29683152 OR 29688834 OR 29698983 OR 29740782 OR 29746264 OR 29770553 OR 29789774 OR 29847241 OR 29869823 OR 29880779 OR 29881639 OR 29932460 OR 29936464 OR 29943097 OR 29944706 OR 29952241 OR 29961039 OR 30021554 OR 30060250 OR 30062572 OR 30067063 OR 30071146 OR 30077574 OR 30077954 OR 30117760 OR 30128855 OR 30129907 OR 30138021 OR 30180192 OR 30202391 OR 30229557 OR 30233121 OR 30244781 OR 30247961 OR 30256657 OR 30272836 OR 30312118 OR 30320304 OR

30339481 OR 30340503 OR 30361831 OR 30368741 OR 30376366 OR 30400784 OR 30402781 OR 30415752 OR 30445595 OR 30485297 OR 30488344 OR 30500981 OR 30539102 OR 30543479 OR 30554276 OR 30554923 OR 30562305 OR 30584782 OR 30609982 OR 30638093 OR 30658629 OR 30659130 OR 30663156 OR 30694978 OR 30723698 OR 30766696 OR 30767964 OR 30775929 OR 30783858 OR 30789349 OR 30794107 OR 30826763 OR 30840336 OR 30850143 OR 30862228 OR 30864301 OR 30874369 OR 30874927 OR 30912010 OR 30912011 OR 30917793 OR 30933317 OR 30972643 OR 30980431 OR 30982113 OR 31003402 OR 31012968 OR 31012970 OR 31014267 OR 31028212 OR 31033073 OR 31041685 OR 31041830 OR 31046885 OR 31076901 OR 31087419 OR 31099634 OR 31121916 OR 31144265 OR 31165653 OR 31168711 OR 31173341 OR 31187251 OR 31187411 OR 31206630 OR 31217021 OR 31227537 OR 31236699 OR 31237570 OR 31244536 OR 31264183 OR 31269917 OR 31272399 OR 31275735 OR 31290395 OR 31309977 OR 31319398 OR 31354000 OR 31367917 OR 31377976 OR 31389108 OR 31395070 OR 31415063 OR 31444192 OR 31454519 OR 31475306 OR 31520215 OR 31561728 OR 31600424 OR 31604709 OR 31605312 OR 31605514 OR 31617073 OR 31617564 OR 31642349 OR 31689771 OR 31694687 OR 31712954 OR 31745553 OR 31752830 OR 31759342 OR 31763746 OR 31773112 OR 31773148 OR 31780003 OR 31791246 OR 31804383 OR 31853701 OR 31860135 OR 31912361 OR 31933148 OR 31964669 OR 31970652 OR 31994181 OR 31996235 OR 31998651 OR 32009481 OR 32019093 OR 32020356 OR 32020706 OR 32031221 OR 32059728 OR 32071117 OR 32090192 OR 32103358 OR 32111767 OR 32112354 OR 32130627 OR 32147571 OR 32193692 OR 32232722 OR 32234652 OR 32240461 OR 32249355 OR 32298326 OR 32298407 OR 32304828 OR 32306100 OR 32307459 OR 32307828 OR 32314053 OR 32314110 OR 32326769 OR 32344683 OR 32348688 OR 32379234 OR 32384481 OR 32385847 OR 32395379 OR 32404725 OR 32406224 OR 32407010 OR 32414347 OR 32415386 OR 32415529 OR 32441691 OR 32443355 OR 32462950 OR 32510461 OR 32521652 OR 32535764 OR 32546796 OR 32558129 OR 32599641 OR 32605075 OR 32607599 OR 32616883 OR 32631302 OR 32664375 OR 32664946 OR 32673270 OR 32673271 OR 32678375 OR 32681303 OR 32694366 OR 32698957 OR 32699997 OR 32754489 OR 32763011 OR 32803384 OR 32808383 OR 32819304 OR 32859615 OR 32868938 OR 32875536 OR 32877271 OR 32883829 OR 32885221 OR 32886189 OR 32906761 OR 32924562 OR 32930924 OR 32940768 OR 32955446 OR 32960338 OR 33010183 OR 33025451 OR 33025453 OR 33036706 OR 33059741 OR 33064204 OR 33072603 OR 33076863 OR 33089371 OR 33105793 OR 33109503 OR 33123481 OR 33128705 OR 33163551 OR 33172957 OR 33178447 OR 33194622 OR 33212425 OR 33218558 OR 33225208 OR 33289669 OR 33292676 OR 33305350 OR 33312762 OR 33312937 OR 33396551 OR 33403402 OR 33423594 OR 33452652 OR 33467265 OR 33473331 OR 33475511 OR 33482396 OR 33491338 OR 33503496 OR 33539554 OR 33550878 OR 33564742 OR 33570477 OR 33588948 OR 33613314 OR 33618699 OR 33624172 OR 33677463 OR 33677781 OR 33689150 OR 33689553 OR 33709302 OR 33737299 OR 33752433 OR 33754246 OR 33786507 OR 33787643 OR 33801189 OR 33807611 OR 33811517 OR 33815828 OR 33823832 OR 33840972 OR 33847588 OR 33849087 OR 33883475 OR 33890857 OR 33896706 OR 33912450 OR 33929533 OR 33935799 OR 33950477 OR 33954891 OR 33956212 OR 33961667 OR 33976224 OR 33977342 OR 33978132 OR 33983599 OR 33989021 OR 33992279 OR 34011028 OR 34049837 OR 34066752 OR 34089422 OR 34125072 OR 34130213 OR 34135964 OR 34146655 OR 34147107 OR 34160294 OR 34161602 OR 34189070 OR 34204528 OR 34207765 OR 34213086 OR 34225091 OR 34258817 OR 34274495 OR 34281126 OR 34290337 OR 34305108 OR 34311713 OR 34313858 OR 34314878 OR 34328623 OR 34344171 OR 34355298 OR 34357555 OR 34359567 OR 34365016 OR 34389552 OR 34413391 OR 34426142 OR 34427806 OR 34436552 OR 34444332 OR 34456250 OR 34461975 OR 34463835 OR 34465518 OR 34467791 OR 34480935 OR 34491527 OR 34498828 OR 34503198 OR 34524631 OR 34546611 OR 34554567 OR 34559724 OR 34567220 OR 34578787 OR 34578984 OR 34579142 OR 34586509 OR 34589720 OR 34590926 OR 34593453 OR 34598902 OR 34609311 OR 34638355 OR 34639353 OR 34649267 OR 34651554 OR 34664019 OR 34674806 OR 34677269 OR 34684403 OR 34709529 OR 34712520 OR 34721081 OR 34729835 OR 34733065 OR 34741653 OR 34777343 OR 34778552 OR 34789309 OR 34812521 OR 34816921 OR 34823494 OR 34836345 OR 34887491 OR 34898001 OR 34922621 OR 34989962 OR 34994945 OR 35012011 OR 35022884 OR 35025064 OR 35040076 OR 35045736 OR 35057525 OR 35087367 OR 35091272 OR 35107399 OR 35109799 OR 35110796 OR 35112212 OR 35130491 OR 35140880 OR 35156143 OR 35171707 OR 35182789 OR 35191900 OR 35200150 OR 35207294 OR 35244811 OR

35246219 OR 35253020 OR 35253682 OR 35257993 OR 35265005 OR 35277360 OR 35278203 OR 35289219 OR 35289926 OR 35314958 OR 35326679 OR 35328964 OR 35331230 OR 35342756 OR 35347550 OR 35363152 OR 35363502 OR 35366505 OR 35391574 OR 35398558 OR 35401377 OR 35416341 OR 35428285 OR 35431214 OR 35441995 OR 35455864 OR 35460441 OR 35471614 OR 35477489 OR 35477651 OR 35486425 OR 35488902 OR 35509876 OR 35533876 OR 35578196 OR 35619910 OR 35620805 OR 35639865 OR 35641019 OR 35681702 OR 35700977 OR 35712474 OR 35731554 OR 35733142 OR 35736016 OR 35738114 OR 35749052 OR 35749369 OR 35750628 OR 35756985 OR 35771607 OR 35775225 OR 35778782 OR 35788372 OR 35795051 OR 35829982 OR 35841719 OR 35853152 OR 35861215 OR 35881067 OR 35894167 OR 35895236 OR 35896295 OR 35902321 OR 35930212 OR 35940850 OR 35952956 OR 35954479 OR 35992855 OR 35993633 OR 35994321 OR 36004842 OR 36012025 OR 36066332 OR 36076044 OR 36084228 OR 36092248 OR 36110260 OR 36141372 OR 36158576 OR 36170550 OR 36201052 OR 36207046 OR 36212795 OR 36220629 OR 36231542 OR 36238756 OR 36254717 OR 36259288 OR 36263723 OR 36269525 OR 36301400 OR 36311982 OR 36314766 OR 36336249 OR 36342348 OR 36400655 OR 36401194 OR 36403344 OR 36407802 OR 36412916 OR 36441715 OR 36451297 OR 36453589 OR 36462139 OR 36468339 OR 36474321 OR 36479658 OR 36482346 OR 36512140 OR 36512157 OR 36517097 OR 36525089 OR 36526826 OR 36535489 OR 36539160 OR 36542098 OR 36542473 OR 36550258 OR 36568235 OR 36579554 OR 36600391 OR 36612340 OR 36618335 OR 36652087 OR 36658635 OR 36674436 OR 36683179 OR 36689989 OR 36692626 OR 36694356 OR 36695128 OR 36711885 OR 36715766 OR 36723801 OR 36736725 OR 36761969 OR 36776335 OR 36777230 OR 36790946 OR 36792620 OR 36800978 OR 36814313 OR 36826074 OR 36828705 OR 36836931 OR 36843066 OR 36847633 OR 36855214 OR 36874708 OR 36890601 OR 36892780 OR 36894449 OR 36901594 OR 36904284 OR 36912039 OR 36920460 OR 36933050)

Table 1b: APA PsycInfo® search strategy

Provider/Interface	Ovid
Database	APA PsycInfo®
Date searched	June 22, 2020; updated May 5, 2022; updated March 20, 2023; updated October 13, 2025
Database update	1806 to June Week 3 2020; update: 1806 to April Week 4 2022; update: 1806 to March Week 2 2023; updated: 1806 to October Week 1 2025
Search developer(s)	Helena VonVille
Limit to English	Yes
Date Range	2013-2020; 2013-2023; 2013-2026
Publication Types	Journal articles only
Search filter source	No search filter used

1	breast neoplasms/ or (breast adj4 (cancer* or neoplasm*)).ti,ab. or (breast cancer* or breast neoplasm*).id.
2	survivors/
3	survivor*.ti,ab,id.
4	1 or 2
5	1 and 4
6	exercise/ or physical activity/ or aerobic exercise/ or weightlifting/ or yoga/
7	(aerobics or biking or bicycling or dance or exercise or jogging or physical activit* or running or swimming or walking or yoga or ((strength or resistance) adj3 training)).ti,ab. or (aerobics or biking or bicycling or dance or exercise or jogging or physical activit* or resistance training or running or strength trainingor swimming or walking or yoga).id.
8	6 or 7
9	5 and 8
10	clinical trials/ or experimental design/
11	randomized controlled trials/ or randomized clinical trials/
12	(clinical trial or empirical study or followup study or longitudinal study or prospective study or treatment outcome).md.
13	((randomi?ed adj7 trial*) or ((single or doubl* or tripl* or treb*) and (blind* or mask*)) or (controlled adj3 trial*) or (clinical adj2 trial*)).ti,ab. or (clinical trial* or controlled trial* or random?ed trial).id.
14	10 or 11 or 12 or 13
15	9 and 14
16	limit 15 to (all journals and english language and yr="2013 - 2020")
	**May 2022 Updated search with items from previous search removed**
16	15 not ((arabic or chinese or dutch or "Farsi (Iranian)" or finnish or french or german or greek or hebrew or hungarian or italian or lithuanian or polish or portuguese or romanian or russian or slovene or slovak or spanish or turkish) not english).la.
17	limit 16 to (all journals and yr="2013 - 2023")
18	17 not (26869680 OR 33312762 OR 32535764 OR 23378138 OR 24176784 OR 29409128 OR 33491338 OR 35486425 OR 32930924 OR 23635341 OR 34355298 OR 34313858 OR 25645134 OR 34554567 OR 30694978 OR 31014267 OR 33010183 OR 26068412 OR 33956212 OR 33754246 OR 30982113 OR 29746264 OR 29168064 OR 33467265 OR 27490111 OR 23274615 OR 30368741 OR 26303657 OR 33929533 OR 30340503 OR 33292676 OR 31309977 OR 23529000 OR 24043292 OR 27738306 OR 27416835 OR 31791246 OR 33218558 OR 29260391 OR 22822181 OR 35477489 OR 26327866 OR 25171662 OR 27543047 OR 28666465 OR 24644304 OR 33977342 OR 35398558 OR

	27524376 OR 23293010 OR 30320304 OR 33312937 OR 35156143 OR 26801931 OR 34589720 OR 26703650 OR 27312845 OR 35277360 OR 31759342 OR 35022884 OR 24212124 OR 33172957 OR 33305350 OR 28632240 OR 35477651 OR 26713501 OR 26315617 OR 31144265 OR 29057279 OR 27988868 OR 30789349 OR 28922074 OR 30554276 OR 24633590 OR 32955446 OR 30912011 OR 30445595 OR 28470368 OR 26840439 OR 29498250 OR 30609982 OR 31168711 OR 31264183 OR 23766391 OR 25903195 OR 31475306 OR 22535636 OR 25148839 OR 31617564 OR 22879576 OR 28525464 OR 34135964 OR 29404842 OR 24419734 OR 23538987 OR 34389552 OR 33475511 OR 29086523 OR 23579251 OR 29617707 OR 28721384 OR 30663156 OR 30862228 OR 29869823 OR 30562305 OR 34609311 OR 32020706 OR 31041685 OR 33815828 OR 26769117 OR 34125072 OR 30554923 OR 27873046 OR 28736768 OR 26000593 OR 26640827 OR 24707505 OR 27857276 OR 26935995 OR 35289219 OR 29644889 OR 34578787 OR 23439424 OR 34311713 OR 35509876 OR 30067063 OR 32599641 OR 34664019 OR 25576214 OR 33064204 OR 27146839 OR 25920528 OR 24805910 OR 30400784 OR 25179578 OR 33624172 OR 35363502 OR 25672583 OR 28070770 OR 24804802 OR 34741653 OR 26970957 OR 25708515 OR 27646264 OR 34463835 OR 28588911 OR 28347291 OR 28624949 OR 27565830 OR 27838922 OR 33613314 OR 26679449 OR 29082588 OR 25394834 OR 23612029 OR 25132146 OR 31600424 OR 30972643 OR 25614924 OR 35253020 OR 31206630 OR 28894513 OR 24648018 OR 33983599 OR 22672363 OR 35182789 OR 34684403 OR 25833781 OR 26984227 OR 33935799 OR 32130627 OR 30339481 OR 26286486 OR 25602030 OR 25567329 OR 27531024 OR 28636526 OR 30485297 OR 23915045 OR 30543479 OR 29243165 OR 34314878 OR 23519041 OR 24310809 OR 26850265 OR 34836345 OR 26395825 OR 32808383 OR 34413391 OR 27925359 OR 28075038 OR 23430010 OR 25313756 OR 28639097 OR 33849087 OR 34147107 OR 35342756 OR 23876573 OR 34465518 OR 31853701 OR 28026901 OR 23213072 OR 24319454 OR 32803384 OR 32859615 OR 34559724 OR 27197276 OR 32694366 OR 30767964 OR 34274495 OR 28427925 OR 31415063 OR 31444192 OR 25965782 OR 31227537 OR 35091272 OR 31605312 OR 26820653 OR 34823494 OR 26199085 OR 32348688 OR 32698957 OR 33787643 OR 34579142 OR 27893938 OR 34066752 OR 26175059 OR 25814054 OR 25730596 OR 34498828 OR 31028212 OR 23017985 OR 34712520 OR 31087419 OR 35140880 OR 33752433 OR 33105793 OR 34305108 OR 29409922 OR 29880779 OR 35012011 OR 30874927 OR 32607599 OR 34777343 OR 33564742 OR 24869977 OR 31689771 OR 31804383 OR 35314958 OR 34204528 OR 28745131 OR 27539586 OR 27530961 OR 28702218 OR 33689150 OR 22831916 OR 35191900 OR 32071117 OR 32960338 OR 31617073 OR 32875536 OR 34207765 OR 34651554 OR 26880252 OR 29356607 OR 23184120 OR 35471614 OR 34359567 OR 28414894 OR 27821958 OR 27635602 OR 27295811 OR 27161493 OR 32019093 OR 28417203 OR 33737299 OR 27893212 OR 31173341 OR 34427806 OR 27110131 OR 27258052 OR 24337598 OR 27742668 OR 33036706 OR 28178355 OR 29026725 OR 33423594 OR 34789309 OR 29470705 OR 29961039 OR 34503198 OR 23963636 OR 34160294 OR 29936464 OR 23989030 OR 34898001 OR 25337546 OR 29259819 OR 32443355 OR 33403402 OR 29654416 OR 35040076 OR 29470804 OR 35366505 OR 23749481 OR 30980431 OR 27516182 OR 29141853 OR 30077574 OR 27417791 OR 25781872 OR 34467791 OR 25853030 OR 25181937 OR 31236699 OR 34357555 OR 31763746 OR 25417174 OR 32404725 OR 29076388 OR 25980790 OR 28551083 OR 23906102 OR 33452652 OR 26373696 OR 32307828 OR 34816921 OR 28410171 OR 24222132 OR 30874369 OR 30840336 OR 23326466 OR 34733065 OR 28965138 OR 27470258 OR 32314110 OR 33163551 OR 33178447 OR 22573338 OR 28392934 OR 28717378 OR 29943097 OR 23575310 OR 25899303 OR 33677781 OR 26782031 OR 26543382 OR 23860950 OR 24894145 OR 35363152 OR 25724409 OR 29511965 OR 32664946 OR 30376366 OR 23620675 OR 34258817 OR 28639157 OR 31121916 OR 32616883 OR 27883168 OR 32020356 OR 31998651 OR 27601139 OR 31994181 OR 34677269 OR 34480935 OR 35244811 OR 26016834 OR 25711667 OR 34146655 OR 25845088 OR 31454519 OR

	26543789 OR 23244677 OR 32510461 OR 24596359 OR 26925999 OR 27912947 OR 28150044 OR 29645327 OR 25975457 OR 26677207 OR 35455864 OR 32415529 OR 33550878 OR 33823832 OR 30864301 OR 26655430 OR 23620504 OR 35110796 OR 32249355 OR 35045736 OR 24495696 OR 25681782 OR 31290395 OR 28228661 OR 33072603 OR 29471477 OR 28665541 OR 30117760 OR 32886189 OR 27737976 OR 23615146 OR 26756114 OR 28620700 OR 28161788 OR 27192633 OR 31319398 OR 26512712 OR 32234652 OR 30488344 OR 33989021 OR 30723698 OR 26304504 OR 30850143 OR 35200150 OR 32111767 OR 25349070 OR 29847241 OR 28991039 OR 31354000 OR 33588948 OR 30912010 OR 33618699 OR 26586495 OR 25901376 OR 30500981 OR 32681303 OR 29932460 OR 33847588 OR 28836379 OR 34649267 OR 30233121 OR 25037667 OR 27498099 OR 23177321 OR 25749601 OR 22996743 OR 26945570 OR 29587682 OR 28253845 OR 27840340 OR 30658629 OR 32232722 OR 34189070 OR 33811517 OR 28189100 OR 35278203 OR 32819304 OR 33978132 OR 26427563 OR 35328964 OR 31244536 OR 34598902 OR 28624715 OR 33194622 OR 34281126 OR 33059741 OR 32885221 OR 27334210 OR 32304828 OR 33539554 OR 27981118 OR 32306100 OR 27718533 OR 32298407 OR 23026681 OR 34639353 OR 26855719 OR 32031221 OR 24916951 OR 33992279 OR 32462950 OR 30584782 OR 34922621 OR 23504137 OR 32868938 OR 33801189 OR 34444332 OR 35416341 OR 24390808 OR 27531738 OR 35428285 OR 26251693 OR 27562357 OR 27887610 OR 25711654 OR 33883475 OR 24997174 OR 29526243 OR 28410164 OR 28900822 OR 27254272 OR 33089371 OR 24065550 OR 30229557 OR 27333128 OR 29388687 OR 33570477 OR 24335923 OR 31520215 OR 22710259 OR 23604998 OR 25833659 OR 25545411 OR 28258052 OR 25338995 OR 24870782 OR 29273954 OR 34586509 OR 35257993 OR 32521652 OR 31642349 OR 29593021 OR 34456250 OR 25073541 OR 32605075 OR 28935620 OR 28561685 OR 30256657 OR 28410162 OR 23054109 OR 26284121 OR 24389524 OR 27793125 OR 27863058 OR 32558129 OR 34225091 OR 28427084 OR 32664375 OR 22544562 OR 32379234 OR 29043460 OR 33076863 OR 26602701 OR 24845177 OR 27061740 OR 25060288 OR 31012968 OR 32924562 OR 30766696 OR 28845551 OR 33840972 OR 33482396 OR 35171707 OR 35107399 OR 34130213 OR 31605514 OR 31377976 OR 24123482 OR 29390904 OR 24569944 OR 28444534 OR 33542853 OR 25792854 OR 31745553 OR 34365016 OR 29698983 OR 34567220 OR 27832718 OR 31187251 OR 28288595 OR 31773148 OR 25367403 OR 31165653 OR 31217021 OR 31237570 OR 30180192 OR 26915025 OR 26059936 OR 26896305 OR 34674806 OR 32877271 OR 27037811 OR 23940231 OR 23947581 OR 24739260 OR 24317968 OR 31970652 OR 25088804 OR 23439658 OR 29553651 OR 27529826 OR 29944706 OR 32940768 OR 31389108 OR 33689553 OR 28756991 OR 23564803 OR 28856470 OR 25304986 OR 31561728 OR 24578086 OR 23514347 OR 29330081 OR 34812521 OR 28166765 OR 25784579 OR 23242613 OR 32414347 OR 27612561 OR 30402781 OR 32059728 OR 34344171 OR 29402761 OR 33289669 OR 34994945 OR 28097626 OR 26110777 OR 29637645 OR 31269917 OR 25110844 OR 28766098 OR 28247126 OR 23402226 OR 34328623 OR 32673271 OR 33912450 OR 30272836 OR 35246219 OR 34436552 OR 30244781 OR 31604709 OR 23456557 OR 30361831 OR 26026737 OR 28538261 OR 23179496 OR 32240461 OR 31046885 OR 26051073 OR 26923090 OR 27232866 OR 34491527 OR 32193692 OR 25975675 OR 29881639 OR 29252103 OR 25752971 OR 27857248 OR 32546796 OR 23443319 OR 27821934 OR 27368882 OR 29579014 OR 27668296 OR 32385847 OR 28410174 OR 23505170 OR 33954891 OR 30138021 OR 29770553 OR 33212425 OR 24105359 OR 29789774 OR 27884071 OR 35441995 OR 34721081 OR 26606746 OR 24834485 OR 24882370 OR 32678375 OR 24131016 OR 30794107 OR 25832894 OR 31694687 OR 23829442 OR 32103358 OR 23663078 OR 28553684 OR 31367917 OR 26829081 OR 30917793 OR 32407010 OR 24013569 OR 31752830 OR 28602712 OR 32395379 OR 23515464 OR 27409075 OR 30021554 OR 30202391 OR 34778552 OR 30129907 OR 27464488 OR 27252076 OR 24486636 OR 29501739 OR 29344401 OR 31996235 OR 23041586 OR 25427610 OR 30128855 OR 32009481 OR 35265005 OR 27543065 OR

	26932848 OR 23888337 OR 35025064 OR 33396551 OR 24596360 OR 26518022 OR 29740782 OR 32112354 OR 27433356 OR 24911404 OR 26988367 OR 35326679 OR 34590926 OR 28698390 OR 31012970 OR 30312118 OR 28926676 OR 23918953 OR 25124456 OR 35289926 OR 26887585 OR 30062572 OR 24708832 OR 25452437 OR 32090192 OR 30071146 OR 27129840 OR 32147571 OR 30247961 OR 26598750 OR 28627254 OR 34089422 OR 34161602 OR 33503496 OR 34887491 OR 33976224 OR 27187092 OR 34426142 OR 34638355 OR 27629548 OR 33473331 OR 32406224 OR 29188593 OR 26706665 OR 29688834 OR 23266440 OR 27909546 OR 23488617 OR 29319387 OR 32307459 OR 32673270 OR 30826763 OR 31912361 OR 31712954 OR 33786507 OR 30638093 OR 32415386 OR 26715294 OR 35087367 OR 26593858 OR 32906761 OR 33677463 OR 29683152 OR 32326769 OR 28411330 OR 26282657 OR 33961667 OR 31033073 OR 33950477 OR 25417947 OR 31773112 OR 29187408 OR 26059937 OR 31003402 OR 35057525 OR 32699997 OR 29395306 OR 24894838 OR 30783858 OR 29474729 OR 33109503 OR 28084890 OR 34593453 OR 35331230 OR 28634625 OR 35391574 OR 33807611 OR 33896706 OR 28838886 OR 27752993 OR 22451113 OR 34546611 OR 26202538 OR 33128705 OR 32298326 OR 23907996 OR 27785639 OR 30775929 OR 25751587 OR 28881471 OR 31076901 OR 29097298 OR 34049837 OR 32754489 OR 31964669 OR 27881518 OR 34461975 OR 27455846 OR 35460441 OR 35253682 OR 32763011 OR 25563360 OR 27100859 OR 31860135 OR 26362355 OR 30077954 OR 34989962 OR 31187411 OR 30659130 OR 34729835 OR 31041830 OR 35431214 OR 29222705 OR 28489508 OR 25793000 OR 24254037 OR 25749595 OR 25555831 OR 33025451 OR 32314053 OR 33890857 OR 29325658 OR 23799886 OR 27332968 OR 25757733 OR 35207294 OR 26973913 OR 33709302 OR 26960972 OR 31272399 OR 24192320 OR 31275735 OR 30415752 OR 32344683 OR 32384481 OR 27858198 OR 28470507 OR 26777589 OR 35347550 OR 33123481 OR 25526904 OR 27976933 OR 23793468 OR 28012121 OR 24829866 OR 23969632 OR 25186211 OR 35109799 OR 29162619 OR 25338320 OR 30539102 OR 35130491 OR 27766453 OR 31933148 OR 27226430 OR 33025453 OR 28741202 OR 31780003 OR 35488902 OR 27293508 OR 30060250 OR 28444532 OR 27868156 OR 27557405 OR 35112212 OR 34011028 OR 31395070 OR 26714371 OR 22864470 OR 23370582 OR 29129704 OR 27865259 OR 24856854 OR 32883829 OR 35401377 OR 25935584 OR 25964257 OR 26605003 OR 24987274 OR 24891269 OR 31099634 OR 32631302 OR 29478430 OR 32441691 OR 29952241 OR 34578984 OR 28739540 OR 28242595 OR 28803675 OR 25739642 OR 25336068 OR 34213086 OR 33225208 OR 34524631 OR 30933317 OR 24703167 OR 34290337 OR 24470004).pm.
19	18 not (2016-29696-001 OR 2013-07538-019 OR 2018-05745-001 OR 2013-13121-009 OR 2014-50780-012 OR 2020-19179-001 OR 2016-28154-001 OR 2018-21990-004 OR 2014-44687-005 OR 2018-03660-054 OR 2014-48138-012 OR 2014-50780-017 OR 2017-08402-003 OR 2015-03463-002 OR 2015-41501-014 OR 2019-47527-001 OR 2013-00455-023 OR 2013-30980-018 OR 2015-44378-013 OR 2015-23963-011 OR 2013-00455-025 OR 2014-08497-013 OR 2019-29841-006 OR 2015-23963-009 OR 2018-45948-001 OR 2015-40705-007 OR 2017-01803-001 OR 2018-26529-001 OR 2013-43202-007 OR 2020-11757-003 OR 2018-07664-007 OR 2020-15834-010 OR 2016-21227-008 OR 2013-07538-014 OR 2015-47242-004 OR 2015-07580-008 OR 2016-29369-002 OR 2015-18465-012 OR 2014-33403-002 OR 2014-35122-009 OR 2017-20548-004 OR 2015-43287-001 OR 2013-38356-007 OR 2013-41342-009 OR 2013-09654-007 OR 2013-06915-012 OR 2019-43054-076 OR 2014-40602-011 OR 2015-16199-002 OR 2016-01027-011 OR 2018-23690-001 OR 2015-50576-015 OR 2013-25635-003 OR 2018-03660-034 OR 2014-45201-007 OR 2017-44272-001 OR 2017-44294-001 OR 2014-50780-014 OR 2013-39685-005 OR 2014-00063-005 OR 2018-44479-001 OR 2015-50886-009 OR 2016-04891-018 OR 2013-04703-013 OR 2017-46246-011 OR 2015-27533-001 OR 2017-08833-001 OR 2016-01558-004 OR 2016-01447-001 OR 2014-39992-006 OR 2015-00629-001 OR 2014-47714-001 OR 2013-31670-012 OR 2019-61351-002 OR 2017-38350-001 OR 2012-30427-001 OR 2013-05692-013 OR 2013-38734-003 OR 2013-04703-009 OR 2014-13065-007 OR 2018-65605-

	010 OR 2016-33872-001 OR 2014-25283-001 OR 2017-12973-001 OR 2013-41607-006 OR 2016-23824-004 OR 2013-14677-009 OR 2013-13874-006 OR 2013-20014-005 OR 2016-04813-015 OR 2015-38214-017 OR 2013-20214-014 OR 2018-65710-013).an.
	**March 2023 Updated search with items from previous search removed**
16	15 not ((arabic or chinese or dutch or "Farsi (Iranian)" or finnish or french or german or greek or hebrew or hungarian or italian or lithuanian or polish or portuguese or romanian or russian or slovene or slovak or spanish or turkish) not english).la.
17	limit 16 to (all journals and yr="2013 - 2023")
18	17 not (22451113 OR 22535636 OR 22544562 OR 22573338 OR 22672363 OR 22710259 OR 22822181 OR 22831916 OR 22864470 OR 22879576 OR 22996743 OR 23017985 OR 23026681 OR 23041586 OR 23054109 OR 23177321 OR 23179496 OR 23184120 OR 23213072 OR 23242613 OR 23244677 OR 23266440 OR 23274615 OR 23293010 OR 23326466 OR 23370582 OR 23378138 OR 23402226 OR 23430010 OR 23439424 OR 23439658 OR 23443319 OR 23456557 OR 23488617 OR 23504137 OR 23505170 OR 23514347 OR 23515464 OR 23519041 OR 23529000 OR 23538987 OR 23564803 OR 23575310 OR 23579251 OR 23604998 OR 23612029 OR 23615146 OR 23620504 OR 23620675 OR 23635341 OR 23663078 OR 23749481 OR 23766391 OR 23793468 OR 23799886 OR 23829442 OR 23860950 OR 23876573 OR 23888337 OR 23906102 OR 23907996 OR 23915045 OR 23918953 OR 23940231 OR 23947581 OR 23963636 OR 23969632 OR 23989030 OR 24013569 OR 24043292 OR 24065550 OR 24105359 OR 24123482 OR 24131016 OR 24176784 OR 24192320 OR 24212124 OR 24222132 OR 24254037 OR 24310809 OR 24317968 OR 24319454 OR 24335923 OR 24337598 OR 24389524 OR 24390808 OR 24419734 OR 24470004 OR 24486636 OR 24495696 OR 24569944 OR 24578086 OR 24596359 OR 24596360 OR 24633590 OR 24644304 OR 24648018 OR 24703167 OR 24707505 OR 24708832 OR 24739260 OR 24804802 OR 24805910 OR 24829866 OR 24834485 OR 24845177 OR 24856854 OR 24869977 OR 24870782 OR 24882370 OR 24891269 OR 24894145 OR 24894838 OR 24911404 OR 24916951 OR 24987274 OR 24997174 OR 25037667 OR 25060288 OR 25073541 OR 25088804 OR 25110844 OR 25124456 OR 25132146 OR 25148839 OR 25171662 OR 25179578 OR 25181937 OR 25186211 OR 25304986 OR 25313756 OR 25336068 OR 25337546 OR 25338320 OR 25338995 OR 25349070 OR 25367403 OR 25394834 OR 25417174 OR 25417947 OR 25427610 OR 25452437 OR 25526904 OR 25545411 OR 25555831 OR 25563360 OR 25567329 OR 25576214 OR 25602030 OR 25614924 OR 25645134 OR 25672583 OR 25681782 OR 25708515 OR 25711654 OR 25711667 OR 25724409 OR 25730596 OR 25739642 OR 25749595 OR 25749601 OR 25751587 OR 25752971 OR 25757733 OR 25781872 OR 25784579 OR 25792854 OR 25793000 OR 25814054 OR 25832894 OR 25833659 OR 25833781 OR 25845088 OR 25853030 OR 25899303 OR 25901376 OR 25903195 OR 25920528 OR 25935584 OR 25964257 OR 25965782 OR 25975457 OR 25975675 OR 25980790 OR 26000593 OR 26016834 OR 26026737 OR 26051073 OR 26059936 OR 26059937 OR 26068412 OR 26110777 OR 26175059 OR 26199085 OR 26202538 OR 26251693 OR 26282657 OR 26284121 OR 26286486 OR 26303657 OR 26304504 OR 26315617 OR 26327866 OR 26362355 OR 26373696 OR 26395825 OR 26427563 OR 26512712 OR 26518022 OR 26543382 OR 26543789 OR 26586495 OR 26593858 OR 26598750 OR 26602701 OR 26605003 OR 26606746 OR 26640827 OR 26655430 OR 26677207 OR 26679449 OR 26703650 OR 26706665 OR 26713501 OR 26714371 OR 26715294 OR 26756114 OR 26769117 OR 26777589 OR 26782031 OR 26801931 OR 26820653 OR 26829081 OR 26840439 OR 26850265 OR 26855719 OR 26869680 OR 26880252 OR 26887585 OR 26896305 OR 26915025 OR 26923090 OR 26925999 OR 26932848 OR 26935995 OR 26945570 OR 26960972 OR 26970957 OR 26973913 OR 26984227 OR 26988367 OR 27037811 OR 27061740 OR 27100859 OR 27110131 OR 27129840 OR 27146839 OR 27161493 OR 27187092 OR 27192633 OR 27197276 OR 27226430 OR 27232866 OR 27252076 OR 27254272 OR 27258052 OR 27293508 OR 27295811 OR 27312845 OR 27332968 OR

	27333128 OR 27334210 OR 27368882 OR 27409075 OR 27416835 OR 27417791 OR 27433356 OR 27455846 OR 27464488 OR 27470258 OR 27490111 OR 27498099 OR 27516182 OR 27524376 OR 27529826 OR 27530961 OR 27531024 OR 27531738 OR 27539586 OR 27543047 OR 27543065 OR 27557405 OR 27562357 OR 27565830 OR 27601139 OR 27612561 OR 27629548 OR 27635602 OR 27646264 OR 27668296 OR 27718533 OR 27737976 OR 27738306 OR 27742668 OR 27752993 OR 27766453 OR 27785639 OR 27793125 OR 27821934 OR 27821958 OR 27832718 OR 27838922 OR 27840340 OR 27857248 OR 27857276 OR 27858198 OR 27863058 OR 27865259 OR 27868156 OR 27873046 OR 27881518 OR 27883168 OR 27884071 OR 27887610 OR 27893212 OR 27893938 OR 27909546 OR 27912947 OR 27925359 OR 27976933 OR 27981118 OR 27988868 OR 28012121 OR 28026901 OR 28070770 OR 28075038 OR 28084890 OR 28097626 OR 28150044 OR 28161788 OR 28166765 OR 28178355 OR 28189100 OR 28228661 OR 28242595 OR 28247126 OR 28253845 OR 28258052 OR 28288595 OR 28347291 OR 28392934 OR 28410162 OR 28410164 OR 28410171 OR 28410174 OR 28411330 OR 28414894 OR 28417203 OR 28427084 OR 28427925 OR 28444532 OR 28444534 OR 28470368 OR 28470507 OR 28489508 OR 28525464 OR 28538261 OR 28551083 OR 28553684 OR 28561685 OR 28588911 OR 28602712 OR 28620700 OR 28624715 OR 28624949 OR 28627254 OR 28632240 OR 28634625 OR 28636526 OR 28639097 OR 28639157 OR 28665541 OR 28666465 OR 28698390 OR 28702218 OR 28717378 OR 28721384 OR 28736768 OR 28739540 OR 28741202 OR 28745131 OR 28756991 OR 28766098 OR 28803675 OR 28836379 OR 28838886 OR 28845551 OR 28856470 OR 28881471 OR 28894513 OR 28900822 OR 28922074 OR 28926676 OR 28935620 OR 28965138 OR 28991039 OR 29026725 OR 29043460 OR 29057279 OR 29076388 OR 29082588 OR 29086523 OR 29097298 OR 29129704 OR 29141853 OR 29162619 OR 29168064 OR 29187408 OR 29188593 OR 29222705 OR 29243165 OR 29252103 OR 29259819 OR 29260391 OR 29273954 OR 29319387 OR 29325658 OR 29330081 OR 29344401 OR 29356607 OR 29388687 OR 29390904 OR 29395306 OR 29402761 OR 29404842 OR 29409128 OR 29409922 OR 29470705 OR 29470804 OR 29471477 OR 29474729 OR 29478430 OR 29498250 OR 29501739 OR 29511965 OR 29526243 OR 29553651 OR 29579014 OR 29587682 OR 29593021 OR 29617707 OR 29637645 OR 29644889 OR 29645327 OR 29654416 OR 29683152 OR 29688834 OR 29698983 OR 29740782 OR 29746264 OR 29770553 OR 29789774 OR 29847241 OR 29869823 OR 29880779 OR 29881639 OR 29932460 OR 29936464 OR 29943097 OR 29944706 OR 29952241 OR 29961039 OR 30021554 OR 30060250 OR 30062572 OR 30067063 OR 30071146 OR 30077574 OR 30077954 OR 30117760 OR 30128855 OR 30129907 OR 30138021 OR 30180192 OR 30202391 OR 30229557 OR 30233121 OR 30244781 OR 30247961 OR 30256657 OR 30272836 OR 30312118 OR 30320304 OR 30339481 OR 30340503 OR 30361831 OR 30368741 OR 30376366 OR 30400784 OR 30402781 OR 30415752 OR 30445595 OR 30485297 OR 30488344 OR 30500981 OR 30539102 OR 30543479 OR 30554276 OR 30554923 OR 30562305 OR 30584782 OR 30609982 OR 30638093 OR 30658629 OR 30659130 OR 30663156 OR 30694978 OR 30723698 OR 30766696 OR 30767964 OR 30775929 OR 30783858 OR 30789349 OR 30794107 OR 30826763 OR 30840336 OR 30850143 OR 30862228 OR 30864301 OR 30874369 OR 30874927 OR 30912010 OR 30912011 OR 30917793 OR 30933317 OR 30972643 OR 30980431 OR 30982113 OR 31003402 OR 31012968 OR 31012970 OR 31014267 OR 31028212 OR 31033073 OR 31041685 OR 31041830 OR 31046885 OR 31076901 OR 31087419 OR 31099634 OR 31121916 OR 31144265 OR 31165653 OR 31168711 OR 31173341 OR 31187251 OR 31187411 OR 31206630 OR 31217021 OR 31227537 OR 31236699 OR 31237570 OR 31244536 OR 31264183 OR 31269917 OR 31272399 OR 31275735 OR 31290395 OR 31309977 OR 31319398 OR 31354000 OR 31367917 OR 31377976 OR 31389108 OR 31395070 OR 31415063 OR 31444192 OR 31454519 OR 31475306 OR 31520215 OR 31561728 OR 31600424 OR 31604709 OR 31605312 OR 31605514 OR 31617073 OR 31617564 OR 31642349 OR 31689771 OR 31694687 OR 31712954 OR 31745553 OR 31752830 OR 31759342 OR

	31763746 OR 31773112 OR 31773148 OR 31780003 OR 31791246 OR 31804383 OR 31853701 OR 31860135 OR 31912361 OR 31933148 OR 31964669 OR 31970652 OR 31994181 OR 31996235 OR 31998651 OR 32009481 OR 32019093 OR 32020356 OR 32020706 OR 32031221 OR 32059728 OR 32071117 OR 32090192 OR 32103358 OR 32111767 OR 32112354 OR 32130627 OR 32147571 OR 32193692 OR 32232722 OR 32234652 OR 32240461 OR 32249355 OR 32298326 OR 32298407 OR 32304828 OR 32306100 OR 32307459 OR 32307828 OR 32314053 OR 32314110 OR 32326769 OR 32344683 OR 32348688 OR 32379234 OR 32384481 OR 32385847 OR 32395379 OR 32404725 OR 32406224 OR 32407010 OR 32414347 OR 32415386 OR 32415529 OR 32441691 OR 32443355 OR 32462950 OR 32510461 OR 32521652 OR 32535764 OR 32546796 OR 32558129 OR 32599641 OR 32605075 OR 32607599 OR 32616883 OR 32631302 OR 32664375 OR 32664946 OR 32673270 OR 32673271 OR 32678375 OR 32681303 OR 32694366 OR 32698957 OR 32699997 OR 32754489 OR 32763011 OR 32803384 OR 32808383 OR 32819304 OR 32859615 OR 32868938 OR 32875536 OR 32877271 OR 32883829 OR 32885221 OR 32886189 OR 32906761 OR 32924562 OR 32930924 OR 32940768 OR 32955446 OR 32960338 OR 33010183 OR 33025451 OR 33025453 OR 33036706 OR 33059741 OR 33064204 OR 33072603 OR 33076863 OR 33089371 OR 33105793 OR 33109503 OR 33123481 OR 33128705 OR 33163551 OR 33172957 OR 33178447 OR 33194622 OR 33212425 OR 33218558 OR 33225208 OR 33289669 OR 33292676 OR 33305350 OR 33312762 OR 33312937 OR 33396551 OR 33403402 OR 33423594 OR 33452652 OR 33467265 OR 33473331 OR 33475511 OR 33482396 OR 33491338 OR 33503496 OR 33539554 OR 33550878 OR 33564742 OR 33570477 OR 33588948 OR 33613314 OR 33618699 OR 33624172 OR 33677463 OR 33677781 OR 33689150 OR 33689553 OR 33709302 OR 33737299 OR 33752433 OR 33754246 OR 33786507 OR 33787643 OR 33801189 OR 33807611 OR 33811517 OR 33815828 OR 33823832 OR 33840972 OR 33847588 OR 33849087 OR 33883475 OR 33890857 OR 33896706 OR 33912450 OR 33929533 OR 33935799 OR 33950477 OR 33954891 OR 33956212 OR 33961667 OR 33976224 OR 33977342 OR 33978132 OR 33983599 OR 33989021 OR 33992279 OR 34011028 OR 34049837 OR 34066752 OR 34089422 OR 34125072 OR 34130213 OR 34135964 OR 34146655 OR 34147107 OR 34160294 OR 34161602 OR 34189070 OR 34204528 OR 34207765 OR 34213086 OR 34225091 OR 34258817 OR 34274495 OR 34281126 OR 34290337 OR 34305108 OR 34311713 OR 34313858 OR 34314878 OR 34328623 OR 34344171 OR 34355298 OR 34357555 OR 34359567 OR 34365016 OR 34389552 OR 34413391 OR 34426142 OR 34427806 OR 34436552 OR 34444332 OR 34456250 OR 34461975 OR 34463835 OR 34465518 OR 34467791 OR 34480935 OR 34491527 OR 34498828 OR 34503198 OR 34524631 OR 34546611 OR 34554567 OR 34559724 OR 34567220 OR 34578787 OR 34578984 OR 34579142 OR 34586509 OR 34589720 OR 34590926 OR 34593453 OR 34598902 OR 34609311 OR 34638355 OR 34639353 OR 34649267 OR 34651554 OR 34664019 OR 34674806 OR 34677269 OR 34684403 OR 34709529 OR 34712520 OR 34721081 OR 34729835 OR 34733065 OR 34741653 OR 34777343 OR 34778552 OR 34789309 OR 34812521 OR 34816921 OR 34823494 OR 34836345 OR 34887491 OR 34898001 OR 34922621 OR 34989962 OR 34994945 OR 35012011 OR 35022884 OR 35025064 OR 35040076 OR 35045736 OR 35057525 OR 35087367 OR 35091272 OR 35107399 OR 35109799 OR 35110796 OR 35112212 OR 35130491 OR 35140880 OR 35156143 OR 35171707 OR 35182789 OR 35191900 OR 35200150 OR 35207294 OR 35244811 OR 35246219 OR 35253020 OR 35253682 OR 35257993 OR 35265005 OR 35277360 OR 35278203 OR 35289219 OR 35289926 OR 35314958 OR 35326679 OR 35328964 OR 35331230 OR 35342756 OR 35347550 OR 35363152 OR 35363502 OR 35366505 OR 35391574 OR 35398558 OR 35401377 OR 35416341 OR 35428285 OR 35431214 OR 35441995 OR 35455864 OR 35460441 OR 35471614 OR 35477489 OR 35477651 OR 35486425 OR 35488902 OR 35509876 OR 35533876 OR 35578196 OR 35619910 OR 35620805 OR 35639865 OR 35641019 OR 35681702 OR 35700977 OR 35712474 OR 35731554 OR 35733142 OR 35736016 OR 35738114 OR 35749052 OR

	35749369 OR 35750628 OR 35756985 OR 35771607 OR 35775225 OR 35778782 OR 35788372 OR 35795051 OR 35829982 OR 35841719 OR 35853152 OR 35861215 OR 35881067 OR 35894167 OR 35895236 OR 35896295 OR 35902321 OR 35930212 OR 35940850 OR 35952956 OR 35954479 OR 35992855 OR 35993633 OR 35994321 OR 36004842 OR 36012025 OR 36066332 OR 36076044 OR 36084228 OR 36092248 OR 36110260 OR 36141372 OR 36158576 OR 36170550 OR 36201052 OR 36207046 OR 36212795 OR 36220629 OR 36231542 OR 36238756 OR 36254717 OR 36259288 OR 36263723 OR 36269525 OR 36301400 OR 36311982 OR 36314766 OR 36336249 OR 36342348 OR 36400655 OR 36401194 OR 36403344 OR 36407802 OR 36412916 OR 36441715 OR 36451297 OR 36453589 OR 36462139 OR 36468339 OR 36474321 OR 36479658 OR 36482346 OR 36512140 OR 36512157 OR 36517097 OR 36525089 OR 36526826 OR 36535489 OR 36539160 OR 36542098 OR 36542473 OR 36550258 OR 36568235 OR 36579554 OR 36600391 OR 36612340 OR 36618335 OR 36652087 OR 36658635 OR 36674436 OR 36683179 OR 36689989 OR 36692626 OR 36694356 OR 36695128 OR 36711885 OR 36715766 OR 36723801 OR 36736725 OR 36761969 OR 36776335 OR 36777230 OR 36790946 OR 36792620 OR 36800978 OR 36814313 OR 36826074 OR 36828705 OR 36836931 OR 36843066 OR 36847633 OR 36855214 OR 36874708 OR 36890601 OR 36892780 OR 36894449 OR 36901594 OR 36904284 OR 36912039 OR 36920460 OR 36933050).pm.
19	18 not (2012-30427-001 OR 2013-00455-023 OR 2013-00455-025 OR 2013-04703-009 OR 2013-04703-013 OR 2013-05692-013 OR 2013-06915-012 OR 2013-07538-014 OR 2013-07538-019 OR 2013-09654-007 OR 2013-13121-009 OR 2013-13874-006 OR 2013-14677-009 OR 2013-20014-005 OR 2013-20214-014 OR 2013-25635-003 OR 2013-30980-018 OR 2013-31670-012 OR 2013-38356-007 OR 2013-38734-003 OR 2013-39685-005 OR 2013-41342-009 OR 2013-41607-006 OR 2013-43202-007 OR 2014-00063-005 OR 2014-08497-013 OR 2014-13065-007 OR 2014-25283-001 OR 2014-33403-002 OR 2014-35122-009 OR 2014-39992-006 OR 2014-40602-011 OR 2014-44687-005 OR 2014-45201-007 OR 2014-47714-001 OR 2014-48138-012 OR 2014-50780-012 OR 2014-50780-014 OR 2014-50780-017 OR 2015-00629-001 OR 2015-03463-002 OR 2015-07580-008 OR 2015-16199-002 OR 2015-18465-012 OR 2015-23963-009 OR 2015-23963-011 OR 2015-27533-001 OR 2015-38214-017 OR 2015-40705-007 OR 2015-41501-014 OR 2015-43287-001 OR 2015-44378-013 OR 2015-47242-004 OR 2015-50576-015 OR 2015-50886-009 OR 2016-01027-011 OR 2016-01447-001 OR 2016-01558-004 OR 2016-04813-015 OR 2016-04891-018 OR 2016-21227-008 OR 2016-23824-004 OR 2016-28154-001 OR 2016-29369-002 OR 2016-29696-001 OR 2016-33872-001 OR 2017-01803-001 OR 2017-08402-003 OR 2017-08833-001 OR 2017-12973-001 OR 2017-20548-004 OR 2017-38350-001 OR 2017-44272-001 OR 2017-44294-001 OR 2017-46246-011 OR 2018-03660-034 OR 2018-03660-054 OR 2018-05745-001 OR 2018-07664-007 OR 2018-21990-004 OR 2018-23690-001 OR 2018-26529-001 OR 2018-44479-001 OR 2018-45948-001 OR 2018-65605-010 OR 2018-65710-013 OR 2019-03823-011 OR 2019-12023-001 OR 2019-29841-006 OR 2019-43054-076 OR 2019-47527-001 OR 2019-58596-001 OR 2019-61351-002 OR 2020-11757-003 OR 2020-11998-001 OR 2020-15834-010 OR 2020-19179-001 OR 2020-29781-001 OR 2020-34988-011 OR 2020-43903-026 OR 2020-49053-006 OR 2020-51713-001 OR 2020-55054-001 OR 2020-57030-007 OR 2020-95076-011 OR 2021-11507-003 OR 2021-11507-016 OR 2021-41826-001 OR 2021-54248-001 OR 2022-12421-001).an.
update October 13, 2025	change to search strategy
1	breast neoplasms/ or (breast adj4 (cancer* or neoplasm*)).ti,ab. or (breast cancer* or breast neoplasm*).id.
2	survivors/ or survivor*.ti,ab,id.
3	1 and 2

4	exercise/ or physical activity/ or aerobic exercise/ or weightlifting/ or yoga/
5	(aerobics or biking or bicycling or dance or exercise or jogging or physical activit* or running or swimming or walking or yoga or ((strength or resistance) adj3 training)).ti,ab. or (aerobics or biking or bicycling or dance or exercise or jogging or physical activit* or resistance training or running or strength trainingor swimming or walking or yoga).id.
6	4 or 5
7	3 and 6
8	clinical trials/ or experimental design/
9	randomized controlled trials/ or randomized clinical trials/
10	(clinical trial or empirical study or followup study or longitudinal study or prospective study or treatment outcome).md.
11	((randomi?ed adj7 trial*) or ((single or doubl* or tripl* or treb*) and (blind* or mask*)) or (controlled adj3 trial*) or (clinical adj2 trial*)).ti,ab. or (clinical trial* or controlled trial* or random?ed trial).id.
12	8 or 9 or 10 or 11
13	7 and 12
14	limit 13 to (all journals and english language and yr="2013 - 2026")
15	14 not (22451113 or 22535636 or 22544562 or 22573338 or 22672363 or 22710259 or 22822181 or 22831916 or 22864470 or 22879576 or 22996743 or 23017985 or 23026681 or 23041586 or 23054109 or 23179496 or 23184120 or 23213072 or 23242613 or 23244677 or 23266440 or 23274615 or 23293010 or 23326466 or 23370582 or 23378138 or 23402226 or 23430010 or 23439424 or 23439658 or 23456557 or 23488617 or 23504137 or 23505170 or 23514347 or 23515464 or 23519041 or 23529000 or 23564803 or 23575310 or 23579251 or 23604998 or 23608116 or 23612029 or 23620504 or 23635341 or 23663078 or 23749481 or 23766391 or 23799886 or 23829442 or 23876573 or 23888337 or 23906102 or 23907996 or 23918953 or 23940231 or 23947581 or 23963636 or 23989030 or 24013569 or 24043292 or 24065550 or 24123482 or 24131016 or 24176784 or 24192320 or 24212124 or 24222132 or 24310809 or 24317968 or 24319454 or 24335923 or 24337598 or 24389524 or 24390808 or 24419734 or 24470004 or 24486636 or 24569944 or 24596359 or 24596360 or 24633590 or 24648018 or 24703167 or 24707505 or 24708832 or 24739260 or 24804802 or 24845177 or 24856854 or 24869977 or 24870782 or 24882370 or 24891269 or 24894145 or 24894838 or 24911404 or 24916951 or 24987274 or 24997174 or 25037667 or 25060288 or 25110844 or 25124456 or 25148839 or 25171662 or 25179578 or 25181937 or 25186211 or 25304986 or 25313756 or 25336068 or 25337546 or 25338995 or 25349070 or 25367403 or 25417174 or 25427610 or 25439319 or 25452437 or 25545411 or 25555831 or 25567329 or 25672583 or 25681782 or 25684491 or 25711667 or 25724409 or 25730596 or 25739642 or 25749595 or 25749601 or 25751587 or 25781872 or 25784579 or 25793000 or 25814054 or 25833659 or 25833781 or 25845088 or 25901376 or 25903195 or 25920528 or 25935584 or 25957010 or 25964257 or 25965782 or 25975457 or 25975675 or 26016834 or 26026737 or 26051073 or 26059936 or 26059937 or 26068412 or 26110777 or 26175059 or 26202538 or 26282657 or 26284121 or 26286486 or 26362355 or 26373696 or 26395825 or 26427563 or 26512712 or 26518022 or 26586495 or 26593858 or 26598750 or 26602701 or 26605003 or 26606746 or 26655430 or 26677207 or 26679449 or 26706665 or 26714371 or 26715294 or 26756114 or 26782031 or 26801931 or 26820653 or 26829081 or 26840439 or 26850265 or 26869680 or 26880252 or 26887585 or 26896305 or 26915025 or 26932848 or 26945570 or 26960972 or 26970957 or 26984227 or 26988367 or 27037811 or 27061740 or 27100859 or 27110131 or 27129840 or 27161493 or 27165213 or 27187092 or 27192633 or 27197276 or 27226430 or 27252076 or 27254272 or 27258052 or 27260627 or 27295811 or 27332968 or 27333128 or 27368882 or 27409075 or 27416835 or 27433356 or 27455846 or 27464488 or 27498099 or 27529826 or 27530961 or 27531024 or 27539586 or 27543047 or 27543065 or 27562357 or 27565830 or 27612561 or 27629548 or 27635602 or 27646264 or 27668296 or 27718533 or 27737976 or 27742668 or 27752993 or 27761518 or 27766453 or 27793125 or 27832718 or

	27838922 or 27840340 or 27863058 or 27865259 or 27873046 or 27881518 or 27883168 or 27884071 or 27887610 or 27893212 or 27893938 or 27909546 or 27912947 or 27925359 or 27988868 or 28026901 or 28070770 or 28075038 or 28084890 or 28097626 or 28150044 or 28166765 or 28178355 or 28189100 or 28228661 or 28242595 or 28253845 or 28258052 or 28288595 or 28347291 or 28392934 or 28410164 or 28410171 or 28411330 or 28414894 or 28417203 or 28427084 or 28427925 or 28444532 or 28470507 or 28489508 or 28538261 or 28547387 or 28551083 or 28561685 or 28588911 or 28624715 or 28627254 or 28634625 or 28636526 or 28639097 or 28639157 or 28698390 or 28702218 or 28721384 or 28739540 or 28745131 or 28803675 or 28836379 or 28838886 or 28881471 or 28900822 or 28922074 or 28926676 or 28935620 or 28965138 or 28991039 or 29076388 or 29082588 or 29086523 or 29097298 or 29129704 or 29141853 or 29162619 or 29168064 or 29187408 or 29222705 or 29243165 or 29252103 or 29259819 or 29273954 or 29319387 or 29325658 or 29330081 or 29344401 or 29356607 or 29388687 or 29390904 or 29395306 or 29402761 or 29409128 or 29409922 or 29470705 or 29470804 or 29471477 or 29474729 or 29478430 or 29501739 or 29511965 or 29526243 or 29553651 or 29587682 or 29617707 or 29637645 or 29654416 or 29683152 or 29688834 or 29698983 or 29713835 or 29740782 or 29746264 or 29770553 or 29847241 or 29869823 or 29880779 or 29881639 or 29932460 or 29936464 or 29943097 or 29944706 or 29952241 or 29961039 or 30021554 or 30060250 or 30062572 or 30067063 or 30071146 or 30077574 or 30077954 or 30117760 or 30128855 or 30136876 or 30138021 or 30180192 or 30202391 or 30229557 or 30233121 or 30247961 or 30272836 or 30312118 or 30339481 or 30340503 or 30368741 or 30376366 or 30400784 or 30402781 or 30415752 or 30485297 or 30500981 or 30539102 or 30554276 or 30554923 or 30562305 or 30584782 or 30658629 or 30659130 or 30663156 or 30694978 or 30766696 or 30783858 or 30789349 or 30826763 or 30840336 or 30850143 or 30864301 or 30874369 or 30896255 or 30912010 or 30917793 or 30933317 or 30972643 or 30982113 or 31012968 or 31012970 or 31014267 or 31028212 or 31033073 or 31041685 or 31041830 or 31046885 or 31076901 or 31087419 or 31099634 or 31121916 or 31144265 or 31165653 or 31168711 or 31173341 or 31187251 or 31187411 or 31227537 or 31236699 or 31244536 or 31264183 or 31269917 or 31272399 or 31275735 or 31309977 or 31319398 or 31354000 or 31389108 or 31395070 or 31415063 or 31420000 or 31444192 or 31454519 or 31520215 or 31561728 or 31600424 or 31605312 or 31605514 or 31617073 or 31689771 or 31694687 or 31712954 or 31745553 or 31759342 or 31763746 or 31773112 or 31773148 or 31780003 or 31791246 or 31804383 or 31853701 or 31860135 or 31912361 or 31933148 or 31964669 or 31970652 or 31994181 or 31996235 or 31998651 or 32019093 or 32020356 or 32020706 or 32059728 or 32071117 or 32090192 or 32103358 or 32111767 or 32112354 or 32147571 or 32173766 or 32193692 or 32234652 or 32249355 or 32277008 or 32298326 or 32298407 or 32304828 or 32306100 or 32307459 or 32307828 or 32314053 or 32348688 or 32384481 or 32385847 or 32395379 or 32404725 or 32406224 or 32414347 or 32415386 or 32415529 or 32441691 or 32443355 or 32462950 or 32510461 or 32521652 or 32546796 or 32599641 or 32605075 or 32616883 or 32664375 or 32664946 or 32673270 or 32681303 or 32698957 or 32699997 or 32763011 or 32803384 or 32808383 or 32819304 or 32859615 or 32875536 or 32883829 or 32885221 or 32886189 or 32924562 or 32930924 or 32940768 or 33010183 or 33025453 or 33036706 or 33059741 or 33064204 or 33072603 or 33089371 or 33123481 or 33163551 or 33172957 or 33178447 or 33212425 or 33218558 or 33225208 or 33289669 or 33292676 or 33305350 or 33312762 or 33312937 or 33396551 or 33403402 or 33423594 or 33452652 or 33482396 or 33491338 or 33503496 or 33539554 or 33542853 or 33564742 or 33588948 or 33618699 or 33624172 or 33677463 or 33677781 or 33689150 or 33689553 or 33709302 or 33737299 or 33752433 or 33754246 or 33786507 or 33787643 or 33801189 or 33807611 or 33840972 or 33847588 or 33848933 or 33849087 or 33877513 or 33883475 or 33890857 or 33896706 or 33912450 or 33929533 or 33935799 or 33954891 or 33961667 or 33976224 or 33977342 or 33978132 or 33992279 or 34011028 or 34018096 or 34049837 or 34066752 or 34089422 or 34125072 or 34130213 or 34147107 or 34160294 or 34161602 or 34189070 or 34204528 or 34213086 or 34225091 or 34258817 or 34274495 or 34290337 or 34291605 or 34305108 or 34311713 or 34314878 or 34328623 or 34344171 or 34355298 or 34359567 or 34365016 or 34389552 or

	34396161 or 34413391 or 34426142 or 34427806 or 34436552 or 34444332 or 34456250 or 34461975 or 34463835 or 34465518 or 34480935 or 34491527 or 34498828 or 34503198 or 34524631 or 34559724 or 34567220 or 34578787 or 34578984 or 34579142 or 34590926 or 34593453 or 34609311 or 34639353 or 34649267 or 34649590 or 34674806 or 34677269 or 34709529 or 34712520 or 34721081 or 34727558 or 34741653 or 34777343 or 34789309 or 34812521 or 34823494 or 34836345 or 34887491 or 34898001 or 34922621 or 34988476 or 34994945 or 35012011 or 35022884 or 35040076 or 35045736 or 35057525 or 35087367 or 35091272 or 35107399 or 35109799 or 35110796 or 35130491 or 35140880 or 35182789 or 35244811 or 35246219 or 35253020 or 35253682 or 35257993 or 35277360 or 35278203 or 35289926 or 35314958 or 35326679 or 35328964 or 35331230 or 35342756 or 35363152 or 35363502 or 35366505 or 35370404 or 35391574 or 35398558 or 35416341 or 35460441 or 35471614 or 35477489 or 35486425 or 35509876 or 35533876 or 35564858 or 35566652 or 35619910 or 35620805 or 35639865 or 35641019 or 35716452 or 35731554 or 35733142 or 35738114 or 35750628 or 35756985 or 35771607 or 35775225 or 35778782 or 35795051 or 35829982 or 35861215 or 35881067 or 35894167 or 35902321 or 35930212 or 35940850 or 35947284 or 35952956 or 35954479 or 35963975 or 35992855 or 35993633 or 35994321 or 36004842 or 36012025 or 36066332 or 36084228 or 36158576 or 36170550 or 36201052 or 36220629 or 36231542 or 36254717 or 36263723 or 36269525 or 36279903 or 36301400 or 36314766 or 36336249 or 36342348 or 36400655 or 36401194 or 36403344 or 36412916 or 36426642 or 36441715 or 36453589 or 36462139 or 36468339 or 36474321 or 36479658 or 36512140 or 36512157 or 36517097 or 36525089 or 36526826 or 36535489 or 36539160 or 36542098 or 36550261 or 36568235 or 36579554 or 36600391 or 36612340 or 36652087 or 36674436 or 36683179 or 36689989 or 36692626 or 36711885 or 36715766 or 36720187 or 36723801 or 36736725 or 36777230 or 36790946 or 36792620 or 36800978 or 36814313 or 36826074 or 36828705 or 36843066 or 36847633 or 36855214 or 36875555 or 36890601 or 36892780 or 36894449 or 36904284 or 36912039 or 36920460 or 36933050 or 36971688 or 36981785 or 36994212 or 37016839 or 37079635 or 37081460 or 37086916 or 37120460 or 37129687 or 37142314 or 37155603 or 37203237 or 37250507 or 37296883 or 37328178 or 37329478 or 37337948 or 37345506 or 37358262 or 37372662 or 37383767 or 37405560 or 37407950 or 37418169 or 37423313 or 37444490 or 37455988 or 37461666 or 37466433 or 37480454 or 37491890 or 37499853 or 37507530 or 37526252 or 37549004 or 37550769 or 37571390 or 37586849 or 37615928 or 37623003 or 37658464 or 37696693 or 37700300 or 37715884 or 37727203 or 37742423 or 37768420 or 37782420 or 37803095 or 37835412 or 37852895 or 37887560 or 37892669 or 37903570 or 37941759 or 37944798 or 37963373 or 37964457 or 37965797 or 37967297 or 38004192 or 38018376 or 38027927 or 38032257 or 38043674 or 38104536 or 38113082 or 38132376 or 38152415 or 38158601 or 38164562 or 38165970 or 38200265 or 38221574 or 38252151 or 38252854 or 38282225 or 38285111 or 38286772 or 38287041 or 38289508 or 38297229 or 38335025 or 38354372 or 38384575 or 38456933 or 38468817 or 38482528 or 38485475 or 38485730 or 38491821 or 38512563 or 38529687 or 38537525 or 38546907 or 38551752 or 38569461 or 38570403 or 38574414 or 38578682 or 38654515 or 38665959 or 38684135 or 38699636 or 38702085 or 38710001 or 38727753 or 38741781 or 38753415 or 38757745 or 38777285 or 38811516 or 38814454 or 38831804 or 38835046 or 38839248 or 38841642 or 38842686 or 38887915 or 38896452 or 38897104 or 38900505 or 38961182 or 38962217 or 38970716 or 38977654 or 39013733 or 39054321 or 39065685 or 39080238 or 39090218 or 39103710 or 39138559 or 39151159 or 39201283 or 39202741 or 39237867 or 39248010 or 39265784 or 39267010 or 39293779 or 39306632 or 39309736 or 39361906 or 39368335 or 39373766 or 39408155 or 39409993 or 39415360 or 39451857 or 39461048 or 39477901 or 39482990 or 39483324 or 39492334 or 39505121 or 39555335 or 39557760 or 39557768 or 39559650 or 39571113 or 39579274 or 39584292 or 39636574 or 39655778 or 39663171 or 39663241 or 39696111 or 39704364 or 39714637 or 39733433 or 39791636 or 39799368 or 39840373 or 39980552 or 39993013 or 39996124 or 39997304 or 40010019 or 40012466 or 40052614 or 40056311 or 40090889 or 40112254 or 40112490 or 40156386 or 40156396 or 40156397 or 40178706 or 40198065 or 40241061 or 40281585 or 40325652 or 40394850 or 40400827 or 40420153 or 40423894 or

	40467527 or 40467967 or 40473936 or 40483630 or 40488447 or 40505072 or 40514607 or 40537089 or 40537190 or 40563617 or 40570265 or 40579648 or 40584872 or 40593196 or 40593357 or 40600532 or 40603677 or 40608178 or 40625889 or 40643869 or 40651067 or 40658949 or 40659404 or 40671809 or 40678279 or 40681723 or 40706110 or 40714912 or 40715644 or 40719638 or 40731797 or 40735810 or 40736469 or 40744079 or 40752821 or 40764740 or 40776791 or 40781207 or 40783771 or 40795071 or 40841694 or 40846340 or 40859515 or 40880350 or 40893728 or 40903011 or 40944158 or 40954654 or 40996607 or 41005365 or 41018515 or 41027933 or 41033915 or 41042936).pm.
16	15 not (2012-30427-001 OR 2013-00455-023 OR 2013-00455-025 OR 2013-04703-009 OR 2013-04703-013 OR 2013-05692-013 OR 2013-06915-012 OR 2013-07538-014 OR 2013-07538-019 OR 2013-09654-007 OR 2013-13121-009 OR 2013-13874-006 OR 2013-14677-009 OR 2013-20014-005 OR 2013-20214-014 OR 2013-25635-003 OR 2013-30980-018 OR 2013-31670-012 OR 2013-38356-007 OR 2013-38734-003 OR 2013-39685-005 OR 2013-41342-009 OR 2013-41607-006 OR 2013-43202-007 OR 2014-00063-005 OR 2014-08497-013 OR 2014-13065-007 OR 2014-25283-001 OR 2014-33403-002 OR 2014-35122-009 OR 2014-39992-006 OR 2014-40602-011 OR 2014-44687-005 OR 2014-45201-007 OR 2014-47714-001 OR 2014-48138-012 OR 2014-50780-012 OR 2014-50780-014 OR 2014-50780-017 OR 2015-00629-001 OR 2015-03463-002 OR 2015-07580-008 OR 2015-16199-002 OR 2015-18465-012 OR 2015-23963-009 OR 2015-23963-011 OR 2015-27533-001 OR 2015-38214-017 OR 2015-40705-007 OR 2015-41501-014 OR 2015-43287-001 OR 2015-44378-013 OR 2015-47242-004 OR 2015-50576-015 OR 2015-50886-009 OR 2016-01027-011 OR 2016-01447-001 OR 2016-01558-004 OR 2016-04813-015 OR 2016-04891-018 OR 2016-21227-008 OR 2016-23824-004 OR 2016-28154-001 OR 2016-29369-002 OR 2016-29696-001 OR 2016-33872-001 OR 2017-01803-001 OR 2017-08402-003 OR 2017-08833-001 OR 2017-12973-001 OR 2017-20548-004 OR 2017-38350-001 OR 2017-44272-001 OR 2017-44294-001 OR 2017-46246-011 OR 2018-03660-034 OR 2018-03660-054 OR 2018-05745-001 OR 2018-07664-007 OR 2018-21990-004 OR 2018-23690-001 OR 2018-26529-001 OR 2018-44479-001 OR 2018-45948-001 OR 2018-65605-010 OR 2018-65710-013 OR 2019-03823-011 OR 2019-12023-001 OR 2019-29841-006 OR 2019-43054-076 OR 2019-47527-001 OR 2019-58596-001 OR 2019-61351-002 OR 2020-11757-003 OR 2020-11998-001 OR 2020-15834-010 OR 2020-19179-001 OR 2020-29781-001 OR 2020-34988-011 OR 2020-43903-026 OR 2020-49053-006 OR 2020-51713-001 OR 2020-55054-001 OR 2020-57030-007 OR 2020-95076-011 OR 2021-11507-003 OR 2021-11507-016 OR 2021-41826-001 OR 2021-54248-001 OR 2022-12421-001).an.

Table 1c: Embase® search strategy

Provider/Interface	Elsevier
Database	Embase®
Date searched	June 25, 2020; update 1: May 5, 2022; update 2: March 20, 2023; update 3: October 13, 2025
Database update	June 25, 2020; update 1: May 5, 2022; update 2: March 20, 2023; update 3: October 13, 2025
Search developer(s)	Helena M. VonVille
Limit to English	Yes
Date Range	Update 1: 2013-2022; Update 2: 2013-2023; Update 3: 2013-2026
Publication Types	Articles, Articles in press, Data papers
Search filter source	No search filter used

1	('breast cancer'/exp OR 'basal like breast cancer'/de OR 'breast carcinogenesis'/de OR 'breast tumor'/de OR 'breast carcinoma'/de OR 'breast adenocarcinoma'/de OR 'breast carcinoma in situ'/de OR 'breast sarcoma'/de OR 'metastatic breast cancer'/de)
2	breast:ti,ab,kw AND (cancer*:ti,ab,kw OR carcinoma*:ti,ab,kw OR neoplasm*:ti,ab,kw OR tumor*:ti,ab,kw OR tumour*:ti,ab,kw)
3	#1 OR #2
4	('cancer survivor'/de OR 'survivor'/de)
5	survivor*:ti,ab,kw
6	#4 OR #5
7	#3 AND #6
8	('anaerobic exercise'/de OR 'exercise'/de OR 'aerobic exercise'/de OR 'aquatic exercise'/de OR 'arm exercise'/de OR 'circuit training'/de OR 'dynamic exercise'/de OR 'endurance training'/de OR 'exercise intensity'/de OR 'high intensity interval training'/de OR 'isokinetic exercise'/de OR 'leg exercise'/de OR 'isometric exercise'/de OR 'isotonic exercise'/de OR 'muscle exercise'/de OR 'stretching exercise'/de OR 'pilates'/de OR 'plyometrics'/de OR 'resistance training'/de OR 'static exercise'/de)
9	aerobics:ti,ab,kw OR ballet:ti,ab,kw OR bicycling:ti,ab,kw OR biking:ti,ab,kw OR calisthenics:ti,ab,kw OR "circuit training":ti,ab,kw OR dance:ti,ab,kw OR exercise:ti,ab,kw OR physical activit*:ti,ab,kw OR "Physical Endurance":ti,ab,kw OR "Plyometric*":ti,ab,kw OR running:ti,ab,kw OR swimming:ti,ab,kw OR "tai chi":ti,ab,kw OR "tai ji":ti,ab,kw OR walking:ti,ab,kw OR yoga:ti,ab,kw OR ((strength:ti,ab,kw OR resistance:ti,ab,kw) AND training:ti,ab,kw)
10	#8 OR #9
11	#7 AND #10
12	('clinical trial'/de OR 'multicenter study'/de OR 'phase 4 clinical trial'/de OR 'controlled clinical trial'/de OR 'randomized controlled trial'/de OR 'community trial'/de OR 'intervention study'/de OR 'prospective study'/de OR 'longitudinal study'/de)
13	('evaluation study'/de OR 'program evaluation'/de OR 'program appropriateness'/de OR 'program acceptability'/de OR 'program effectiveness'/de OR 'program impact'/de)
14	((randomised:ti,ab,kw OR randomized:ti,ab,kw) AND (trial:ti,ab,kw OR trials:ti,ab,kw)) OR ((single:ti,ab,kw OR double:ti,ab,kw OR doubled:ti,ab,kw OR triple:ti,ab,kw OR tripled:ti,ab,kw OR treble:ti,ab,kw OR treble:ti,ab,kw) AND (blind*:ti,ab,kw OR mask*:ti,ab,kw)) OR ('4 arm':ti,ab,kw OR 'four arm':ti,ab,kw)
15	(pre-:ti,ab,kw AND post-:ti,ab,kw) OR (pretest:ti,ab,kw AND posttest:ti,ab,kw) OR (program*:ti,ab,kw AND (evaluate:ti,ab,kw OR evaluated:ti,ab,kw OR evaluates:ti,ab,kw OR evaluating:ti,ab,kw OR evaluation:ti,ab,kw OR evaluations:ti,ab,kw OR evaluator:ti,ab,kw OR evaluators:ti,ab,kw)) OR effectiveness:ti,ab,kw OR intervention:ti,ab,kw

16	#12 OR #13 OR #14 OR #15
17	#11 AND #16
18	#17 AND ([article]/lim OR [article in press]/lim OR [data papers]/lim)
19	#18 AND [english]/lim
	**May 2022 Updated search with items from previous search removed**
20	#19 AND [2013-2022]/py
21	#20 NOT (26869680:ui OR 33312762:ui OR 32535764:ui OR 23378138:ui OR 24176784:ui OR 29409128:ui OR 33491338:ui OR 35486425:ui OR 32930924:ui OR 23635341:ui OR 34355298:ui OR 34313858:ui OR 25645134:ui OR 34554567:ui OR 30694978:ui OR 31014267:ui OR 33010183:ui OR 26068412:ui OR 33956212:ui OR 33754246:ui OR 30982113:ui OR 29746264:ui OR 29168064:ui OR 33467265:ui OR 27490111:ui OR 23274615:ui OR 30368741:ui OR 26303657:ui OR 33929533:ui OR 30340503:ui OR 33292676:ui OR 31309977:ui OR 23529000:ui OR 24043292:ui OR 27738306:ui OR 27416835:ui OR 31791246:ui OR 33218558:ui OR 29260391:ui OR 22822181:ui OR 35477489:ui OR 26327866:ui OR 25171662:ui OR 27543047:ui OR 28666465:ui OR 24644304:ui OR 33977342:ui OR 35398558:ui OR 27524376:ui OR 23293010:ui OR 30320304:ui OR 33312937:ui OR 35156143:ui OR 26801931:ui OR 34589720:ui OR 26703650:ui OR 27312845:ui OR 35277360:ui OR 31759342:ui OR 35022884:ui OR 24212124:ui OR 33172957:ui OR 33305350:ui OR 28632240:ui OR 35477651:ui OR 26713501:ui OR 26315617:ui OR 31144265:ui OR 29057279:ui OR 27988868:ui OR 30789349:ui OR 28922074:ui OR 30554276:ui OR 24633590:ui OR 32955446:ui OR 30912011:ui OR 30445595:ui OR 28470368:ui OR 26840439:ui OR 29498250:ui OR 30609982:ui OR 31168711:ui OR 31264183:ui OR 23766391:ui OR 25903195:ui OR 31475306:ui OR 22535636:ui OR 25148839:ui OR 31617564:ui OR 22879576:ui OR 28525464:ui OR 34135964:ui OR 29404842:ui OR 24419734:ui OR 23538987:ui OR 34389552:ui OR 33475511:ui OR 29086523:ui OR 23579251:ui OR 29617707:ui OR 28721384:ui OR 30663156:ui OR 30862228:ui OR 29869823:ui OR 30562305:ui OR 34609311:ui OR 32020706:ui OR 31041685:ui OR 33815828:ui OR 26769117:ui OR 34125072:ui OR 30554923:ui OR 27873046:ui OR 28736768:ui OR 26000593:ui OR 26640827:ui OR 24707505:ui OR 27857276:ui OR 26935995:ui OR 35289219:ui OR 29644889:ui OR 34578787:ui OR 23439424:ui OR 34311713:ui OR 35509876:ui OR 30067063:ui OR 32599641:ui OR 34664019:ui OR 25576214:ui OR 33064204:ui OR 27146839:ui OR 25920528:ui OR 24805910:ui OR 30400784:ui OR 25179578:ui OR 33624172:ui OR 35363502:ui OR 25672583:ui OR 28070770:ui OR 24804802:ui OR 34741653:ui OR 26970957:ui OR 25708515:ui OR 27646264:ui OR 34463835:ui OR 28588911:ui OR 28347291:ui OR 28624949:ui OR 27565830:ui OR 27838922:ui OR 33613314:ui OR 26679449:ui OR 29082588:ui OR 25394834:ui OR 23612029:ui OR 25132146:ui OR 31600424:ui OR 30972643:ui OR 25614924:ui OR 35253020:ui OR 31206630:ui OR 28894513:ui OR 24648018:ui OR 33983599:ui OR 22672363:ui OR 35182789:ui OR 34684403:ui OR 25833781:ui OR 26984227:ui OR 33935799:ui OR 32130627:ui OR 30339481:ui OR 26286486:ui OR 25602030:ui OR 25567329:ui OR 27531024:ui OR 28636526:ui OR 30485297:ui OR 23915045:ui OR 30543479:ui OR 29243165:ui OR 34314878:ui OR 23519041:ui OR 24310809:ui OR 26850265:ui OR 34836345:ui OR 26395825:ui OR 32808383:ui OR 34413391:ui OR 27925359:ui OR 28075038:ui OR 23430010:ui OR 25313756:ui OR 28639097:ui OR 33849087:ui OR 34147107:ui OR 35342756:ui OR 23876573:ui OR 34465518:ui OR 31853701:ui OR 28026901:ui OR 23213072:ui OR 24319454:ui OR 32803384:ui OR 32859615:ui OR 34559724:ui OR 27197276:ui OR 32694366:ui OR 30767964:ui OR 34274495:ui OR 28427925:ui OR 31415063:ui OR 31444192:ui OR 25965782:ui OR 31227537:ui OR 35091272:ui OR 31605312:ui OR 26820653:ui OR 34823494:ui OR 26199085:ui OR 32348688:ui OR 32698957:ui OR 33787643:ui OR 34579142:ui OR 27893938:ui OR

34066752:ui OR 26175059:ui OR 25814054:ui OR 25730596:ui OR 34498828:ui OR
31028212:ui OR 23017985:ui OR 34712520:ui OR 31087419:ui OR 35140880:ui OR
33752433:ui OR 33105793:ui OR 34305108:ui OR 29409922:ui OR 29880779:ui OR
35012011:ui OR 30874927:ui OR 32607599:ui OR 34777343:ui OR 33564742:ui OR
24869977:ui OR 31689771:ui OR 31804383:ui OR 35314958:ui OR 34204528:ui OR
28745131:ui OR 27539586:ui OR 27530961:ui OR 28702218:ui OR 33689150:ui OR
22831916:ui OR 35191900:ui OR 32071117:ui OR 32960338:ui OR 31617073:ui OR
32875536:ui OR 34207765:ui OR 34651554:ui OR 26880252:ui OR 29356607:ui OR
23184120:ui OR 35471614:ui OR 34359567:ui OR 28414894:ui OR 27821958:ui OR
27635602:ui OR 27295811:ui OR 27161493:ui OR 32019093:ui OR 28417203:ui OR
33737299:ui OR 27893212:ui OR 31173341:ui OR 34427806:ui OR 27110131:ui OR
27258052:ui OR 24337598:ui OR 27742668:ui OR 33036706:ui OR 28178355:ui OR
29026725:ui OR 33423594:ui OR 34789309:ui OR 29470705:ui OR 29961039:ui OR
34503198:ui OR 23963636:ui OR 34160294:ui OR 29936464:ui OR 23989030:ui OR
34898001:ui OR 25337546:ui OR 29259819:ui OR 32443355:ui OR 33403402:ui OR
29654416:ui OR 35040076:ui OR 29470804:ui OR 35366505:ui OR 23749481:ui OR
30980431:ui OR 27516182:ui OR 29141853:ui OR 30077574:ui OR 27417791:ui OR
25781872:ui OR 34467791:ui OR 25853030:ui OR 25181937:ui OR 31236699:ui OR
34357555:ui OR 31763746:ui OR 25417174:ui OR 32404725:ui OR 29076388:ui OR
25980790:ui OR 28551083:ui OR 23906102:ui OR 33452652:ui OR 26373696:ui OR
32307828:ui OR 34816921:ui OR 28410171:ui OR 24222132:ui OR 30874369:ui OR
30840336:ui OR 23326466:ui OR 34733065:ui OR 28965138:ui OR 27470258:ui OR
32314110:ui OR 33163551:ui OR 33178447:ui OR 22573338:ui OR 28392934:ui OR
28717378:ui OR 29943097:ui OR 23575310:ui OR 25899303:ui OR 33677781:ui OR
26782031:ui OR 26543382:ui OR 23860950:ui OR 24894145:ui OR 35363152:ui OR
25724409:ui OR 29511965:ui OR 32664946:ui OR 30376366:ui OR 23620675:ui OR
34258817:ui OR 28639157:ui OR 31121916:ui OR 32616883:ui OR 27883168:ui OR
32020356:ui OR 31998651:ui OR 27601139:ui OR 31994181:ui OR 34677269:ui OR
34480935:ui OR 35244811:ui OR 26016834:ui OR 25711667:ui OR 34146655:ui OR
25845088:ui OR 31454519:ui OR 26543789:ui OR 23244677:ui OR 32510461:ui OR
24596359:ui OR 26925999:ui OR 27912947:ui OR 28150044:ui OR 29645327:ui OR
25975457:ui OR 26677207:ui OR 35455864:ui OR 32415529:ui OR 33550878:ui OR
33823832:ui OR 30864301:ui OR 26655430:ui OR 23620504:ui OR 35110796:ui OR
32249355:ui OR 35045736:ui OR 24495696:ui OR 25681782:ui OR 31290395:ui OR
28228661:ui OR 33072603:ui OR 29471477:ui OR 28665541:ui OR 30117760:ui OR
32886189:ui OR 27737976:ui OR 23615146:ui OR 26756114:ui OR 28620700:ui OR
28161788:ui OR 27192633:ui OR 31319398:ui OR 26512712:ui OR 32234652:ui OR
30488344:ui OR 33989021:ui OR 30723698:ui OR 26304504:ui OR 30850143:ui OR
35200150:ui OR 32111767:ui OR 25349070:ui OR 29847241:ui OR 28991039:ui OR
31354000:ui OR 33588948:ui OR 30912010:ui OR 33618699:ui OR 26586495:ui OR
25901376:ui OR 30500981:ui OR 32681303:ui OR 29932460:ui OR 33847588:ui OR
28836379:ui OR 34649267:ui OR 30233121:ui OR 25037667:ui OR 27498099:ui OR
23177321:ui OR 25749601:ui OR 22996743:ui OR 26945570:ui OR 29587682:ui OR
28253845:ui OR 27840340:ui OR 30658629:ui OR 32232722:ui OR 34189070:ui OR
33811517:ui OR 28189100:ui OR 35278203:ui OR 32819304:ui OR 33978132:ui OR
26427563:ui OR 35328964:ui OR 31244536:ui OR 34598902:ui OR 28624715:ui OR
33194622:ui OR 34281126:ui OR 33059741:ui OR 32885221:ui OR 27334210:ui OR
32304828:ui OR 33539554:ui OR 27981118:ui OR 32306100:ui OR 27718533:ui OR
32298407:ui OR 23026681:ui OR 34639353:ui OR 26855719:ui OR 32031221:ui OR
24916951:ui OR 33992279:ui OR 32462950:ui OR 30584782:ui OR 34922621:ui OR
23504137:ui OR 32868938:ui OR 33801189:ui OR 34444332:ui OR 35416341:ui OR
24390808:ui OR 27531738:ui OR 35428285:ui OR 26251693:ui OR 27562357:ui OR
27887610:ui OR 25711654:ui OR 33883475:ui OR 24997174:ui OR 29526243:ui OR
28410164:ui OR 28900822:ui OR 27254272:ui OR 33089371:ui OR 24065550:ui OR

	30229557:ui OR 27333128:ui OR 29388687:ui OR 33570477:ui OR 24335923:ui OR<br>31520215:ui OR 22710259:ui OR 23604998:ui OR 25833659:ui OR 25545411:ui OR<br>28258052:ui OR 25338995:ui OR 24870782:ui OR 29273954:ui OR 34586509:ui OR<br>35257993:ui OR 32521652:ui OR 31642349:ui OR 29593021:ui OR 34456250:ui OR<br>25073541:ui OR 32605075:ui OR 28935620:ui OR 28561685:ui OR 30256657:ui OR<br>28410162:ui OR 23054109:ui OR 26284121:ui OR 24389524:ui OR 27793125:ui OR<br>27863058:ui OR 32558129:ui OR 34225091:ui OR 28427084:ui OR 32664375:ui OR<br>22544562:ui OR 32379234:ui OR 29043460:ui OR 33076863:ui OR 26602701:ui OR<br>24845177:ui OR 27061740:ui OR 25060288:ui OR 31012968:ui OR 32924562:ui OR<br>30766696:ui OR 28845551:ui OR 33840972:ui OR 33482396:ui OR 35171707:ui OR<br>35107399:ui OR 34130213:ui OR 31605514:ui OR 31377976:ui OR 24123482:ui OR<br>29390904:ui OR 24569944:ui OR 28444534:ui OR 33542853:ui OR 25792854:ui OR<br>31745553:ui OR 34365016:ui OR 29698983:ui OR 34567220:ui OR 27832718:ui OR<br>31187251:ui OR 28288595:ui OR 31773148:ui OR 25367403:ui OR 31165653:ui OR<br>31217021:ui OR 31237570:ui OR 30180192:ui OR 26915025:ui OR 26059936:ui OR<br>26896305:ui OR 34674806:ui OR 32877271:ui OR 27037811:ui OR 23940231:ui OR<br>23947581:ui OR 24739260:ui OR 24317968:ui OR 31970652:ui OR 25088804:ui OR<br>23439658:ui OR 29553651:ui OR 27529826:ui OR 29944706:ui OR 32940768:ui OR<br>31389108:ui OR 33689553:ui OR 28756991:ui OR 23564803:ui OR 28856470:ui OR<br>25304986:ui OR 31561728:ui OR 24578086:ui OR 23514347:ui OR 29330081:ui OR<br>34812521:ui OR 28166765:ui OR 25784579:ui OR 23242613:ui OR 32414347:ui OR<br>27612561:ui OR 30402781:ui OR 32059728:ui OR 34344171:ui OR 29402761:ui OR<br>33289669:ui OR 34994945:ui OR 28097626:ui OR 26110777:ui OR 29637645:ui OR<br>31269917:ui OR 25110844:ui OR 28766098:ui OR 28247126:ui OR 23402226:ui OR<br>34328623:ui OR 32673271:ui OR 33912450:ui OR 30272836:ui OR 35246219:ui OR<br>34436552:ui OR 30244781:ui OR 31604709:ui OR 23456557:ui OR 30361831:ui OR<br>26026737:ui OR 28538261:ui OR 23179496:ui OR 32240461:ui OR 31046885:ui OR<br>26051073:ui OR 26923090:ui OR 27232866:ui OR 34491527:ui OR 32193692:ui OR<br>25975675:ui OR 29881639:ui OR 29252103:ui OR 25752971:ui OR 27857248:ui OR<br>32546796:ui OR 23443319:ui OR 27821934:ui OR 27368882:ui OR 29579014:ui OR<br>27668296:ui OR 32385847:ui OR 28410174:ui OR 23505170:ui OR 33954891:ui OR<br>30138021:ui OR 29770553:ui OR 33212425:ui OR 24105359:ui OR 29789774:ui OR<br>27884071:ui OR 35441995:ui OR 34721081:ui OR 26606746:ui OR 24834485:ui OR<br>24882370:ui OR 32678375:ui OR 24131016:ui OR 30794107:ui OR 25832894:ui OR<br>31694687:ui OR 23829442:ui OR 32103358:ui OR 23663078:ui OR 28553684:ui OR<br>31367917:ui OR 26829081:ui OR 30917793:ui OR 32407010:ui OR 24013569:ui OR<br>31752830:ui OR 28602712:ui OR 32395379:ui OR 23515464:ui OR 27409075:ui OR<br>30021554:ui OR 30202391:ui OR 34778552:ui OR 30129907:ui OR 27464488:ui OR<br>27252076:ui OR 24486636:ui OR 29501739:ui OR 29344401:ui OR 31996235:ui OR<br>23041586:ui OR 25427610:ui OR 30128855:ui OR 32009481:ui OR 35265005:ui OR<br>27543065:ui OR 26932848:ui OR 23888337:ui OR 35025064:ui OR 33396551:ui OR<br>24596360:ui OR 26518022:ui OR 29740782:ui OR 32112354:ui OR 27433356:ui OR<br>24911404:ui OR 26988367:ui OR 35326679:ui OR 34590926:ui OR 28698390:ui OR<br>31012970:ui OR 30312118:ui OR 28926676:ui OR 23918953:ui OR 25124456:ui OR<br>35289926:ui OR 26887585:ui OR 30062572:ui OR 24708832:ui OR 25452437:ui OR<br>32090192:ui OR 30071146:ui OR 27129840:ui OR 32147571:ui OR 30247961:ui OR<br>26598750:ui OR 28627254:ui OR 34089422:ui OR 34161602:ui OR 33503496:ui OR<br>34887491:ui OR 33976224:ui OR 27187092:ui OR 34426142:ui OR 34638355:ui OR<br>27629548:ui OR 33473331:ui OR 32406224:ui OR 29188593:ui OR 26706665:ui OR<br>29688834:ui OR 23266440:ui OR 27909546:ui OR 23488617:ui OR 29319387:ui OR<br>32307459:ui OR 32673270:ui OR 30826763:ui OR 31912361:ui OR 31712954:ui OR<br>33786507:ui OR 30638093:ui OR 32415386:ui OR 26715294:ui OR 35087367:ui OR<br>26593858:ui OR 32906761:ui OR 33677463:ui OR 29683152:ui OR 32326769:ui OR<br>28411330:ui OR 26282657:ui OR 33961667:ui OR 31033073:ui OR 33950477:ui OR

	25417947:ui OR 31773112:ui OR 29187408:ui OR 26059937:ui OR 31003402:ui OR 35057525:ui OR 32699997:ui OR 29395306:ui OR 24894838:ui OR 30783858:ui OR 29474729:ui OR 33109503:ui OR 28084890:ui OR 34593453:ui OR 35331230:ui OR 28634625:ui OR 35391574:ui OR 33807611:ui OR 33896706:ui OR 28838886:ui OR 27752993:ui OR 22451113:ui OR 34546611:ui OR 26202538:ui OR 33128705:ui OR 32298326:ui OR 23907996:ui OR 27785639:ui OR 30775929:ui OR 25751587:ui OR 28881471:ui OR 31076901:ui OR 29097298:ui OR 34049837:ui OR 32754489:ui OR 31964669:ui OR 27881518:ui OR 34461975:ui OR 27455846:ui OR 35460441:ui OR 35253682:ui OR 32763011:ui OR 25563360:ui OR 27100859:ui OR 31860135:ui OR 26362355:ui OR 30077954:ui OR 34989962:ui OR 31187411:ui OR 30659130:ui OR 34729835:ui OR 31041830:ui OR 35431214:ui OR 29222705:ui OR 28489508:ui OR 25793000:ui OR 24254037:ui OR 25749595:ui OR 25555831:ui OR 33025451:ui OR 32314053:ui OR 33890857:ui OR 29325658:ui OR 23799886:ui OR 27332968:ui OR 25757733:ui OR 35207294:ui OR 26973913:ui OR 33709302:ui OR 26960972:ui OR 31272399:ui OR 24192320:ui OR 31275735:ui OR 30415752:ui OR 32344683:ui OR 32384481:ui OR 27858198:ui OR 28470507:ui OR 26777589:ui OR 35347550:ui OR 33123481:ui OR 25526904:ui OR 27976933:ui OR 23793468:ui OR 28012121:ui OR 24829866:ui OR 23969632:ui OR 25186211:ui OR 35109799:ui OR 29162619:ui OR 25338320:ui OR 30539102:ui OR 35130491:ui OR 27766453:ui OR 31933148:ui OR 27226430:ui OR 33025453:ui OR 28741202:ui OR 31780003:ui OR 35488902:ui OR 27293508:ui OR 30060250:ui OR 28444532:ui OR 27868156:ui OR 27557405:ui OR 35112212:ui OR 34011028:ui OR 31395070:ui OR 26714371:ui OR 22864470:ui OR 23370582:ui OR 29129704:ui OR 27865259:ui OR 24856854:ui OR 32883829:ui OR 35401377:ui OR 25935584:ui OR 25964257:ui OR 26605003:ui OR 24987274:ui OR 24891269:ui OR 31099634:ui OR 32631302:ui OR 29478430:ui OR 32441691:ui OR 29952241:ui OR 34578984:ui OR 28739540:ui OR 28242595:ui OR 28803675:ui OR 25739642:ui OR 25336068:ui OR 34213086:ui OR 33225208:ui OR 34524631:ui OR 30933317:ui OR 24703167:ui OR 34290337:ui OR 24470004:ui)
22	#21 NOT (L2004213604:an OR L618024921:an OR L621313978:an OR L608886223:an OR L2002711216:an OR L628959553:an OR L52555312:an OR L608561063:an OR L52565658:an OR L625229244:an OR L615271273:an OR L626730189:an OR L628279065:an OR L616142672:an OR L605609389:an OR L607924117:an OR L631807844:an OR L620586989:an OR L600142265:an OR L2002709267:an OR L603289820:an OR L616879941:an OR L2002738219:an OR L52177351:an OR L607030060:an OR L626330149:an OR L52346123:an OR L620404486:an OR L614400856:an OR L624411021:an OR L618332824:an OR L631056884:an OR L607342849:an OR L617188119:an OR L622188278:an OR L627105684:an OR L53015657:an OR L619429847:an OR L629742090:an OR L631481961:an OR L2001078602:an OR L619905629:an OR L630324517:an OR L2004454291:an OR L2003479541:an OR L600224982:an OR L614133805:an OR L622131813:an OR L2003382155:an OR L2004229495:an OR L622441017:an OR L369338009:an OR L626237321:an OR L617727911:an OR L615221376:an OR L614521889:an OR L2002777051:an OR L2005164693:an OR L2004465943:an OR L613222581:an OR L604328654:an OR L604778618:an OR L368956618:an OR L628754993:an OR L616745154:an OR L623794814:an OR L52407873:an OR L614364570:an OR L622904891:an OR L369116620:an OR L372232613:an OR L607132520:an OR L613228719:an OR L603646984:an OR L2002394223:an OR L627212438:an OR L2002842553:an OR L627105521:an OR L369338755:an OR L373883705:an OR L612752357:an OR L620712028:an OR L52498998:an OR L2003439466:an OR L611639801:an OR L624516729:an OR L628831742:an OR L2002879057:an OR L631056612:an OR L617261272:an OR L607384595:an OR L373482131:an OR L373747305:an OR L372885445:an OR L607061919:an OR L627801557:an OR L616056465:an OR L615715402:an OR L624162880:an OR L369380088:an OR L52503157:an OR L622789887:an OR L626439506:an OR L627135496:an OR

	L2002136690:an OR L630951125:an OR L600346372:an OR L603620183:an OR L2003183624:an OR L616155069:an OR L602628656:an OR L605114137:an OR L601148833:an)
25	**March 2023 Updated search with items from previous searches removed**
20	#19 AND [2013-2023]/py
21	#20 not (22451113:ui OR 22535636:ui OR 22544562:ui OR 22573338:ui OR 22672363:ui OR 22710259:ui OR 22822181:ui OR 22831916:ui OR 22864470:ui OR 22879576:ui OR 22996743:ui OR 23017985:ui OR 23026681:ui OR 23041586:ui OR 23054109:ui OR 23177321:ui OR 23179496:ui OR 23184120:ui OR 23213072:ui OR 23242613:ui OR 23244677:ui OR 23266440:ui OR 23274615:ui OR 23293010:ui OR 23326466:ui OR 23370582:ui OR 23378138:ui OR 23402226:ui OR 23430010:ui OR 23439424:ui OR 23439658:ui OR 23443319:ui OR 23456557:ui OR 23488617:ui OR 23504137:ui OR 23505170:ui OR 23514347:ui OR 23515464:ui OR 23519041:ui OR 23529000:ui OR 23538987:ui OR 23564803:ui OR 23575310:ui OR 23579251:ui OR 23604998:ui OR 23612029:ui OR 23615146:ui OR 23620504:ui OR 23620675:ui OR 23635341:ui OR 23663078:ui OR 23749481:ui OR 23766391:ui OR 23793468:ui OR 23799886:ui OR 23829442:ui OR 23860950:ui OR 23876573:ui OR 23888337:ui OR 23906102:ui OR 23907996:ui OR 23915045:ui OR 23918953:ui OR 23940231:ui OR 23947581:ui OR 23963636:ui OR 23969632:ui OR 23989030:ui OR 24013569:ui OR 24043292:ui OR 24065550:ui OR 24105359:ui OR 24123482:ui OR 24131016:ui OR 24176784:ui OR 24192320:ui OR 24212124:ui OR 24222132:ui OR 24254037:ui OR 24310809:ui OR 24317968:ui OR 24319454:ui OR 24335923:ui OR 24337598:ui OR 24389524:ui OR 24390808:ui OR 24419734:ui OR 24470004:ui OR 24486636:ui OR 24495696:ui OR 24569944:ui OR 24578086:ui OR 24596359:ui OR 24596360:ui OR 24633590:ui OR 24644304:ui OR 24648018:ui OR 24703167:ui OR 24707505:ui OR 24708832:ui OR 24739260:ui OR 24804802:ui OR 24805910:ui OR 24829866:ui OR 24834485:ui OR 24845177:ui OR 24856854:ui OR 24869977:ui OR 24870782:ui OR 24882370:ui OR 24891269:ui OR 24894145:ui OR 24894838:ui OR 24911404:ui OR 24916951:ui OR 24987274:ui OR 24997174:ui OR 25037667:ui OR 25060288:ui OR 25073541:ui OR 25088804:ui OR 25110844:ui OR 25124456:ui OR 25132146:ui OR 25148839:ui OR 25171662:ui OR 25179578:ui OR 25181937:ui OR 25186211:ui OR 25304986:ui OR 25313756:ui OR 25336068:ui OR 25337546:ui OR 25338320:ui OR 25338995:ui OR 25349070:ui OR 25367403:ui OR 25394834:ui OR 25417174:ui OR 25417947:ui OR 25427610:ui OR 25452437:ui OR 25526904:ui OR 25545411:ui OR 25555831:ui OR 25563360:ui OR 25567329:ui OR 25576214:ui OR 25602030:ui OR 25614924:ui OR 25645134:ui OR 25672583:ui OR 25681782:ui OR 25708515:ui OR 25711654:ui OR 25711667:ui OR 25724409:ui OR 25730596:ui OR 25739642:ui OR 25749595:ui OR 25749601:ui OR 25751587:ui OR 25752971:ui OR 25757733:ui OR 25781872:ui OR 25784579:ui OR 25792854:ui OR 25793000:ui OR 25814054:ui OR 25832894:ui OR 25833659:ui OR 25833781:ui OR 25845088:ui OR 25853030:ui OR 25899303:ui OR 25901376:ui OR 25903195:ui OR 25920528:ui OR 25935584:ui OR 25964257:ui OR 25965782:ui OR 25975457:ui OR 25975675:ui OR 25980790:ui OR 26000593:ui OR 26016834:ui OR 26026737:ui OR 26051073:ui OR 26059936:ui OR 26059937:ui OR 26068412:ui OR 26110777:ui OR 26175059:ui OR 26199085:ui OR 26202538:ui OR 26251693:ui OR 26282657:ui OR 26284121:ui OR 26286486:ui OR 26303657:ui OR 26304504:ui OR 26315617:ui OR 26327866:ui OR 26362355:ui OR 26373696:ui OR 26395825:ui OR 26427563:ui OR 26512712:ui OR 26518022:ui OR 26543382:ui OR 26543789:ui OR 26586495:ui OR 26593858:ui OR 26598750:ui OR 26602701:ui OR 26605003:ui OR 26606746:ui OR 26640827:ui OR 26655430:ui OR 26677207:ui OR 26679449:ui OR 26703650:ui OR 26706665:ui OR 26713501:ui OR 26714371:ui OR 26715294:ui OR 26756114:ui OR 26769117:ui OR 26777589:ui OR 26782031:ui OR 26801931:ui OR 26820653:ui OR 26829081:ui OR 26840439:ui OR 26850265:ui OR 26855719:ui OR 26869680:ui OR 26880252:ui OR 26887585:ui OR 26896305:ui OR

	26915025:ui OR 26923090:ui OR 26925999:ui OR 26932848:ui OR 26935995:ui OR 26945570:ui OR 26960972:ui OR 26970957:ui OR 26973913:ui OR 26984227:ui OR 26988367:ui OR 27037811:ui OR 27061740:ui OR 27100859:ui OR 27110131:ui OR 27129840:ui OR 27146839:ui OR 27161493:ui OR 27187092:ui OR 27192633:ui OR 27197276:ui OR 27226430:ui OR 27232866:ui OR 27252076:ui OR 27254272:ui OR 27258052:ui OR 27293508:ui OR 27295811:ui OR 27312845:ui OR 27332968:ui OR 27333128:ui OR 27334210:ui OR 27368882:ui OR 27409075:ui OR 27416835:ui OR 27417791:ui OR 27433356:ui OR 27455846:ui OR 27464488:ui OR 27470258:ui OR 27490111:ui OR 27498099:ui OR 27516182:ui OR 27524376:ui OR 27529826:ui OR 27530961:ui OR 27531024:ui OR 27531738:ui OR 27539586:ui OR 27543047:ui OR 27543065:ui OR 27557405:ui OR 27562357:ui OR 27565830:ui OR 27601139:ui OR 27612561:ui OR 27629548:ui OR 27635602:ui OR 27646264:ui OR 27668296:ui OR 27718533:ui OR 27737976:ui OR 27738306:ui OR 27742668:ui OR 27752993:ui OR 27766453:ui OR 27785639:ui OR 27793125:ui OR 27821934:ui OR 27821958:ui OR 27832718:ui OR 27838922:ui OR 27840340:ui OR 27857248:ui OR 27857276:ui OR 27858198:ui OR 27863058:ui OR 27865259:ui OR 27868156:ui OR 27873046:ui OR 27881518:ui OR 27883168:ui OR 27884071:ui OR 27887610:ui OR 27893212:ui OR 27893938:ui OR 27909546:ui OR 27912947:ui OR 27925359:ui OR 27976933:ui OR 27981118:ui OR 27988868:ui OR 28012121:ui OR 28026901:ui OR 28070770:ui OR 28075038:ui OR 28084890:ui OR 28097626:ui OR 28150044:ui OR 28161788:ui OR 28166765:ui OR 28178355:ui OR 28189100:ui OR 28228661:ui OR 28242595:ui OR 28247126:ui OR 28253845:ui OR 28258052:ui OR 28288595:ui OR 28347291:ui OR 28392934:ui OR 28410162:ui OR 28410164:ui OR 28410171:ui OR 28410174:ui OR 28411330:ui OR 28414894:ui OR 28417203:ui OR 28427084:ui OR 28427925:ui OR 28444532:ui OR 28444534:ui OR 28470368:ui OR 28470507:ui OR 28489508:ui OR 28525464:ui OR 28538261:ui OR 28551083:ui OR 28553684:ui OR 28561685:ui OR 28588911:ui OR 28602712:ui OR 28620700:ui OR 28624715:ui OR 28624949:ui OR 28627254:ui OR 28632240:ui OR 28634625:ui OR 28636526:ui OR 28639097:ui OR 28639157:ui OR 28665541:ui OR 28666465:ui OR 28698390:ui OR 28702218:ui OR 28717378:ui OR 28721384:ui OR 28736768:ui OR 28739540:ui OR 28741202:ui OR 28745131:ui OR 28756991:ui OR 28766098:ui OR 28803675:ui OR 28836379:ui OR 28838886:ui OR 28845551:ui OR 28856470:ui OR 28881471:ui OR 28894513:ui OR 28900822:ui OR 28922074:ui OR 28926676:ui OR 28935620:ui OR 28965138:ui OR 28991039:ui OR 29026725:ui OR 29043460:ui OR 29057279:ui OR 29076388:ui OR 29082588:ui OR 29086523:ui OR 29097298:ui OR 29129704:ui OR 29141853:ui OR 29162619:ui OR 29168064:ui OR 29187408:ui OR 29188593:ui OR 29222705:ui OR 29243165:ui OR 29252103:ui OR 29259819:ui OR 29260391:ui OR 29273954:ui OR 29319387:ui OR 29325658:ui OR 29330081:ui OR 29344401:ui OR 29356607:ui OR 29388687:ui OR 29390904:ui OR 29395306:ui OR 29402761:ui OR 29404842:ui OR 29409128:ui OR 29409922:ui OR 29470705:ui OR 29470804:ui OR 29471477:ui OR 29474729:ui OR 29478430:ui OR 29498250:ui OR 29501739:ui OR 29511965:ui OR 29526243:ui OR 29553651:ui OR 29579014:ui OR 29587682:ui OR 29593021:ui OR 29617707:ui OR 29637645:ui OR 29644889:ui OR 29645327:ui OR 29654416:ui OR 29683152:ui OR 29688834:ui OR 29698983:ui OR 29740782:ui OR 29746264:ui OR 29770553:ui OR 29789774:ui OR 29847241:ui OR 29869823:ui OR 29880779:ui OR 29881639:ui OR 29932460:ui OR 29936464:ui OR 29943097:ui OR 29944706:ui OR 29952241:ui OR 29961039:ui OR 30021554:ui OR 30060250:ui OR 30062572:ui OR 30067063:ui OR 30071146:ui OR 30077574:ui OR 30077954:ui OR 30117760:ui OR 30128855:ui OR 30129907:ui OR 30138021:ui OR 30180192:ui OR 30202391:ui OR 30229557:ui OR 30233121:ui OR 30244781:ui OR 30247961:ui OR 30256657:ui OR 30272836:ui OR 30312118:ui OR 30320304:ui OR 30339481:ui OR 30340503:ui OR 30361831:ui OR 30368741:ui OR 30376366:ui OR 30400784:ui OR 30402781:ui OR 30415752:ui OR 30445595:ui OR 30485297:ui OR 30488344:ui OR 30500981:ui OR 30539102:ui OR 30543479:ui OR 30554276:ui OR 30554923:ui OR 30562305:ui OR

	30584782:ui OR 30609982:ui OR 30638093:ui OR 30658629:ui OR 30659130:ui OR<br>30663156:ui OR 30694978:ui OR 30723698:ui OR 30766696:ui OR 30767964:ui OR<br>30775929:ui OR 30783858:ui OR 30789349:ui OR 30794107:ui OR 30826763:ui OR<br>30840336:ui OR 30850143:ui OR 30862228:ui OR 30864301:ui OR 30874369:ui OR<br>30874927:ui OR 30912010:ui OR 30912011:ui OR 30917793:ui OR 30933317:ui OR<br>30972643:ui OR 30980431:ui OR 30982113:ui OR 31003402:ui OR 31012968:ui OR<br>31012970:ui OR 31014267:ui OR 31028212:ui OR 31033073:ui OR 31041685:ui OR<br>31041830:ui OR 31046885:ui OR 31076901:ui OR 31087419:ui OR 31099634:ui OR<br>31121916:ui OR 31144265:ui OR 31165653:ui OR 31168711:ui OR 31173341:ui OR<br>31187251:ui OR 31187411:ui OR 31206630:ui OR 31217021:ui OR 31227537:ui OR<br>31236699:ui OR 31237570:ui OR 31244536:ui OR 31264183:ui OR 31269917:ui OR<br>31272399:ui OR 31275735:ui OR 31290395:ui OR 31309977:ui OR 31319398:ui OR<br>31354000:ui OR 31367917:ui OR 31377976:ui OR 31389108:ui OR 31395070:ui OR<br>31415063:ui OR 31444192:ui OR 31454519:ui OR 31475306:ui OR 31520215:ui OR<br>31561728:ui OR 31600424:ui OR 31604709:ui OR 31605312:ui OR 31605514:ui OR<br>31617073:ui OR 31617564:ui OR 31642349:ui OR 31689771:ui OR 31694687:ui OR<br>31712954:ui OR 31745553:ui OR 31752830:ui OR 31759342:ui OR 31763746:ui OR<br>31773112:ui OR 31773148:ui OR 31780003:ui OR 31791246:ui OR 31804383:ui OR<br>31853701:ui OR 31860135:ui OR 31912361:ui OR 31933148:ui OR 31964669:ui OR<br>31970652:ui OR 31994181:ui OR 31996235:ui OR 31998651:ui OR 32009481:ui OR<br>32019093:ui OR 32020356:ui OR 32020706:ui OR 32031221:ui OR 32059728:ui OR<br>32071117:ui OR 32090192:ui OR 32103358:ui OR 32111767:ui OR 32112354:ui OR<br>32130627:ui OR 32147571:ui OR 32193692:ui OR 32232722:ui OR 32234652:ui OR<br>32240461:ui OR 32249355:ui OR 32298326:ui OR 32298407:ui OR 32304828:ui OR<br>32306100:ui OR 32307459:ui OR 32307828:ui OR 32314053:ui OR 32314110:ui OR<br>32326769:ui OR 32344683:ui OR 32348688:ui OR 32379234:ui OR 32384481:ui OR<br>32385847:ui OR 32395379:ui OR 32404725:ui OR 32406224:ui OR 32407010:ui OR<br>32414347:ui OR 32415386:ui OR 32415529:ui OR 32441691:ui OR 32443355:ui OR<br>32462950:ui OR 32510461:ui OR 32521652:ui OR 32535764:ui OR 32546796:ui OR<br>32558129:ui OR 32599641:ui OR 32605075:ui OR 32607599:ui OR 32616883:ui OR<br>32631302:ui OR 32664375:ui OR 32664946:ui OR 32673270:ui OR 32673271:ui OR<br>32678375:ui OR 32681303:ui OR 32694366:ui OR 32698957:ui OR 32699997:ui OR<br>32754489:ui OR 32763011:ui OR 32803384:ui OR 32808383:ui OR 32819304:ui OR<br>32859615:ui OR 32868938:ui OR 32875536:ui OR 32877271:ui OR 32883829:ui OR<br>32885221:ui OR 32886189:ui OR 32906761:ui OR 32924562:ui OR 32930924:ui OR<br>32940768:ui OR 32955446:ui OR 32960338:ui OR 33010183:ui OR 33025451:ui OR<br>33025453:ui OR 33036706:ui OR 33059741:ui OR 33064204:ui OR 33072603:ui OR<br>33076863:ui OR 33089371:ui OR 33105793:ui OR 33109503:ui OR 33123481:ui OR<br>33128705:ui OR 33163551:ui OR 33172957:ui OR 33178447:ui OR 33194622:ui OR<br>33212425:ui OR 33218558:ui OR 33225208:ui OR 33289669:ui OR 33292676:ui OR<br>33305350:ui OR 33312762:ui OR 33312937:ui OR 33396551:ui OR 33403402:ui OR<br>33423594:ui OR 33452652:ui OR 33467265:ui OR 33473331:ui OR 33475511:ui OR<br>33482396:ui OR 33491338:ui OR 33503496:ui OR 33539554:ui OR 33550878:ui OR<br>33564742:ui OR 33570477:ui OR 33588948:ui OR 33613314:ui OR 33618699:ui OR<br>33624172:ui OR 33677463:ui OR 33677781:ui OR 33689150:ui OR 33689553:ui OR<br>33709302:ui OR 33737299:ui OR 33752433:ui OR 33754246:ui OR 33786507:ui OR<br>33787643:ui OR 33801189:ui OR 33807611:ui OR 33811517:ui OR 33815828:ui OR<br>33823832:ui OR 33840972:ui OR 33847588:ui OR 33849087:ui OR 33883475:ui OR<br>33890857:ui OR 33896706:ui OR 33912450:ui OR 33929533:ui OR 33935799:ui OR<br>33950477:ui OR 33954891:ui OR 33956212:ui OR 33961667:ui OR 33976224:ui OR<br>33977342:ui OR 33978132:ui OR 33983599:ui OR 33989021:ui OR 33992279:ui OR<br>34011028:ui OR 34049837:ui OR 34066752:ui OR 34089422:ui OR 34125072:ui OR<br>34130213:ui OR 34135964:ui OR 34146655:ui OR 34147107:ui OR 34160294:ui OR<br>34161602:ui OR 34189070:ui OR 34204528:ui OR 34207765:ui OR 34213086:ui OR

	34225091:ui OR 34258817:ui OR 34274495:ui OR 34281126:ui OR 34290337:ui OR 34305108:ui OR 34311713:ui OR 34313858:ui OR 34314878:ui OR 34328623:ui OR 34344171:ui OR 34355298:ui OR 34357555:ui OR 34359567:ui OR 34365016:ui OR 34389552:ui OR 34413391:ui OR 34426142:ui OR 34427806:ui OR 34436552:ui OR 34444332:ui OR 34456250:ui OR 34461975:ui OR 34463835:ui OR 34465518:ui OR 34467791:ui OR 34480935:ui OR 34491527:ui OR 34498828:ui OR 34503198:ui OR 34524631:ui OR 34546611:ui OR 34554567:ui OR 34559724:ui OR 34567220:ui OR 34578787:ui OR 34578984:ui OR 34579142:ui OR 34586509:ui OR 34589720:ui OR 34590926:ui OR 34593453:ui OR 34598902:ui OR 34609311:ui OR 34638355:ui OR 34639353:ui OR 34649267:ui OR 34651554:ui OR 34664019:ui OR 34674806:ui OR 34677269:ui OR 34684403:ui OR 34709529:ui OR 34712520:ui OR 34721081:ui OR 34729835:ui OR 34733065:ui OR 34741653:ui OR 34777343:ui OR 34778552:ui OR 34789309:ui OR 34812521:ui OR 34816921:ui OR 34823494:ui OR 34836345:ui OR 34887491:ui OR 34898001:ui OR 34922621:ui OR 34989962:ui OR 34994945:ui OR 35012011:ui OR 35022884:ui OR 35025064:ui OR 35040076:ui OR 35045736:ui OR 35057525:ui OR 35087367:ui OR 35091272:ui OR 35107399:ui OR 35109799:ui OR 35110796:ui OR 35112212:ui OR 35130491:ui OR 35140880:ui OR 35156143:ui OR 35171707:ui OR 35182789:ui OR 35191900:ui OR 35200150:ui OR 35207294:ui OR 35244811:ui OR 35246219:ui OR 35253020:ui OR 35253682:ui OR 35257993:ui OR 35265005:ui OR 35277360:ui OR 35278203:ui OR 35289219:ui OR 35289926:ui OR 35314958:ui OR 35326679:ui OR 35328964:ui OR 35331230:ui OR 35342756:ui OR 35347550:ui OR 35363152:ui OR 35363502:ui OR 35366505:ui OR 35391574:ui OR 35398558:ui OR 35401377:ui OR 35416341:ui OR 35428285:ui OR 35431214:ui OR 35441995:ui OR 35455864:ui OR 35460441:ui OR 35471614:ui OR 35477489:ui OR 35477651:ui OR 35486425:ui OR 35488902:ui OR 35509876:ui OR 35533876:ui OR 35578196:ui OR 35619910:ui OR 35620805:ui OR 35639865:ui OR 35641019:ui OR 35681702:ui OR 35700977:ui OR 35712474:ui OR 35731554:ui OR 35733142:ui OR 35736016:ui OR 35738114:ui OR 35749052:ui OR 35749369:ui OR 35750628:ui OR 35756985:ui OR 35771607:ui OR 35775225:ui OR 35778782:ui OR 35788372:ui OR 35795051:ui OR 35829982:ui OR 35841719:ui OR 35853152:ui OR 35861215:ui OR 35881067:ui OR 35894167:ui OR 35895236:ui OR 35896295:ui OR 35902321:ui OR 35930212:ui OR 35940850:ui OR 35952956:ui OR 35954479:ui OR 35992855:ui OR 35993633:ui OR 35994321:ui OR 36004842:ui OR 36012025:ui OR 36066332:ui OR 36076044:ui OR 36084228:ui OR 36092248:ui OR 36110260:ui OR 36141372:ui OR 36158576:ui OR 36170550:ui OR 36201052:ui OR 36207046:ui OR 36212795:ui OR 36220629:ui OR 36231542:ui OR 36238756:ui OR 36254717:ui OR 36259288:ui OR 36263723:ui OR 36269525:ui OR 36301400:ui OR 36311982:ui OR 36314766:ui OR 36336249:ui OR 36342348:ui OR 36400655:ui OR 36401194:ui OR 36403344:ui OR 36407802:ui OR 36412916:ui OR 36441715:ui OR 36451297:ui OR 36453589:ui OR 36462139:ui OR 36468339:ui OR 36474321:ui OR 36479658:ui OR 36482346:ui OR 36512140:ui OR 36512157:ui OR 36517097:ui OR 36525089:ui OR 36526826:ui OR 36535489:ui OR 36539160:ui OR 36542098:ui OR 36542473:ui OR 36550258:ui OR 36568235:ui OR 36579554:ui OR 36600391:ui OR 36612340:ui OR 36618335:ui OR 36652087:ui OR 36658635:ui OR 36674436:ui OR 36683179:ui OR 36689989:ui OR 36692626:ui OR 36694356:ui OR 36695128:ui OR 36711885:ui OR 36715766:ui OR 36723801:ui OR 36736725:ui OR 36761969:ui OR 36776335:ui OR 36777230:ui OR 36790946:ui OR 36792620:ui OR 36800978:ui OR 36814313:ui OR 36826074:ui OR 36828705:ui OR 36836931:ui OR 36843066:ui OR 36847633:ui OR 36855214:ui OR 36874708:ui OR 36890601:ui OR 36892780:ui OR 36894449:ui OR 36901594:ui OR 36904284:ui OR 36912039:ui OR 36920460:ui OR 36933050:ui)
22	#21 not (22926087:ui OR 23225743:ui OR 23352440:ui OR 23438359:ui OR 23494869:ui OR 23519566:ui OR 23576689:ui OR 23596106:ui OR 23647980:ui OR 23695926:ui OR 23775970:ui OR 23844919:ui OR 23957903:ui OR 24554388:ui OR 24753546:ui OR 24934783:ui OR 25144625:ui OR 25153791:ui OR 25537522:ui OR 25637285:ui OR

	25668509:ui OR 25695466:ui OR 25957010:ui OR 26059937:ui OR 26252280:ui OR 26324364:ui OR 26343172:ui OR 26503386:ui OR 26651471:ui OR 26809289:ui OR 26882537:ui OR 27151593:ui OR 27387096:ui OR 27529756:ui OR 27832718:ui OR 27881009:ui OR 28094664:ui OR 28133022:ui OR 28154107:ui OR 28187725:ui OR 28322759:ui OR 28391397:ui OR 28453622:ui OR 28497387:ui OR 28642039:ui OR 28806753:ui OR 28929925:ui OR 29270829:ui OR 29523666:ui OR 29649913:ui OR 29864112:ui OR 29951713:ui OR 30056387:ui OR 30136876:ui OR 30272598:ui OR 30286723:ui OR 30312190:ui OR 30535101:ui OR 30652562:ui OR 30710242:ui OR 30783858:ui OR 30802224:ui OR 30919252:ui OR 30943543:ui OR 30950445:ui OR 30969906:ui OR 30972643:ui OR 31094843:ui OR 31120271:ui OR 31157323:ui OR 31267779:ui OR 31274335:ui OR 31330023:ui OR 31375907:ui OR 31524020:ui OR 31617590:ui OR 31672949:ui OR 31780003:ui OR 31805025:ui OR 31925532:ui OR 32020433:ui OR 32060166:ui OR 32172321:ui OR 32173766:ui OR 32202706:ui OR 32207884:ui OR 32239145:ui OR 32277008:ui OR 32371507:ui OR 32506338:ui OR 32554133:ui OR 32571351:ui OR 32575442:ui OR 32618207:ui OR 32642951:ui OR 32645252:ui OR 32658377:ui OR 32715730:ui OR 32926116:ui OR 32935450:ui OR 33029754:ui OR 33107982:ui OR 33185782:ui OR 33221667:ui OR 33243013:ui OR 33484565:ui OR 33507443:ui OR 33565036:ui OR 33721925:ui OR 33821320:ui OR 33883478:ui OR 33899978:ui OR 34018096:ui OR 34050402:ui OR 34242744:ui OR 34289212:ui OR 34315289:ui OR 34353286:ui OR 34380488:ui OR 34387728:ui OR 34452552:ui OR 34499632:ui OR 34620115:ui OR 34825858:ui OR 34840208:ui OR 34853914:ui OR 34895583:ui OR 34948517:ui OR 35064432:ui OR 35098347:ui)
23	#22 NOT (l2001078602 OR l2002136690 OR l2002394223 OR l2002709267 OR l2002711216 OR l2002738219 OR l2002777051 OR l2002842553 OR l2002879057 OR l2003183624 OR l2003382155 OR l2003439466 OR l2003479541 OR l2004048127 OR l2004213604 OR l2004229495 OR l2004454291 OR l2004465943 OR l2004474406 OR l2004501225 OR l2004581621 OR l2004711273 OR l2005131908 OR l2005153371 OR l2005164693 OR l2005290693 OR l2005473372 OR l2005519102 OR l2005519240 OR l2005697435 OR l2005843605 OR l2005852118 OR l2005893027 OR l2006733731 OR l2006919579 OR l2007019558 OR l2007027068 OR l2007081793 OR l2007460952 OR l2007973516 OR l2007973737 OR l2008387513 OR l2010039293 OR l2010303414 OR l2010489508 OR l2010621175 OR l2011025169 OR l2011070432 OR l2011391203 OR l2011670402 OR l2012331407 OR l2013210947 OR l2013309680 OR l2013337637 OR l2013378513 OR l2013408646 OR l2013444748 OR l2013558001 OR l2013891223 OR l2013928639 OR l2014072488 OR l2014306533 OR l2014343872 OR l2014774962 OR l2014799366 OR l2014873775 OR l2014904875 OR l2015008830 OR l2015207786 OR l2015237148 OR l2015800934 OR l2015943523 OR l2016158539 OR l2016556154 OR l2016581939 OR l2016908618 OR l2017015461 OR l2017492561 OR l368956618 OR l369116620 OR l369338009 OR l369338755 OR l369380088 OR l372232613 OR l372885445 OR l373482131 OR l373747305 OR l373883705 OR l52177351 OR l52346123 OR l52407873 OR l52498998 OR l52503157 OR l52555312 OR l52565658 OR l53015657 OR l600142265 OR l600224982 OR l600346372 OR l601148833 OR l602628656 OR l603289820 OR l603620183 OR l603646984 OR l604328654 OR l605114137 OR l605609389 OR l606179334 OR l607030060 OR l607061919 OR l607132520 OR l607342849 OR l607384595 OR l607924117 OR l608561063 OR l608886223 OR l611639801 OR l612752357 OR l613222581 OR l613228719 OR l613318391 OR l614133805 OR l614364570 OR l614400856 OR l614521889 OR l615221376 OR l615271273 OR l615715402 OR l616056465 OR l616142672 OR l616155069 OR l616745154 OR l616879941 OR l617188119 OR l617261272 OR l617727911 OR l618024921 OR l618332824 OR l619429847 OR l619905629 OR l620404486 OR l620586989 OR l620712028 OR l621313978 OR l622131813 OR l622188278 OR l622441017 OR l622789887 OR l622904891 OR l623794814 OR l624162880 OR l624411021 OR l624516729 OR l625229244 OR l626237321 OR l626330149 OR l626439506 OR l626730189 OR l627105521 OR l627105684 OR l627135496 OR

	l627212438 OR l627801557 OR l628279065 OR l628611564 OR l628754993 OR l628831742 OR l628959553 OR l629742090 OR l630324517 OR l630951125 OR l631056612 OR l631056884 OR l631261515 OR l631422502 OR l631481961 OR l631807844 OR l632912007 OR l632948309 OR l633137060 OR l633280368 OR l633413983 OR l634246668 OR l634716876 OR l635136064 OR l635274975 OR l636017419 OR l636108074)
update October 13, 2025	
20	#19 AND [2013-2026]/py
21	#20 NOT (22451113:ui OR 22535636:ui OR 22544562:ui OR 22573338:ui OR 22672363:ui OR 22710259:ui OR 22822181:ui OR 22831916:ui OR 22864470:ui OR 22879576:ui OR 22996743:ui OR 23017985:ui OR 23026681:ui OR 23041586:ui OR 23054109:ui OR 23177321:ui OR 23179496:ui OR 23184120:ui OR 23213072:ui OR 23242613:ui OR 23244677:ui OR 23266440:ui OR 23274615:ui OR 23293010:ui OR 23326466:ui OR 23370582:ui OR 23378138:ui OR 23402226:ui OR 23430010:ui OR 23439424:ui OR 23439658:ui OR 23443319:ui OR 23456557:ui OR 23488617:ui OR 23504137:ui OR 23505170:ui OR 23514347:ui OR 23515464:ui OR 23519041:ui OR 23529000:ui OR 23538987:ui OR 23564803:ui OR 23575310:ui OR 23579251:ui OR 23604998:ui OR 23612029:ui OR 23615146:ui OR 23620504:ui OR 23620675:ui OR 23635341:ui OR 23663078:ui OR 23749481:ui OR 23766391:ui OR 23793468:ui OR 23799886:ui OR 23829442:ui OR 23860950:ui OR 23876573:ui OR 23888337:ui OR 23906102:ui OR 23907996:ui OR 23915045:ui OR 23918953:ui OR 23940231:ui OR 23947581:ui OR 23963636:ui OR 23969632:ui OR 23989030:ui OR 24013569:ui OR 24043292:ui OR 24065550:ui OR 24105359:ui OR 24123482:ui OR 24131016:ui OR 24176784:ui OR 24192320:ui OR 24212124:ui OR 24222132:ui OR 24254037:ui OR 24310809:ui OR 24317968:ui OR 24319454:ui OR 24335923:ui OR 24337598:ui OR 24389524:ui OR 24390808:ui OR 24419734:ui OR 24470004:ui OR 24486636:ui OR 24495696:ui OR 24569944:ui OR 24578086:ui OR 24596359:ui OR 24596360:ui OR 24633590:ui OR 24644304:ui OR 24648018:ui OR 24703167:ui OR 24707505:ui OR 24708832:ui OR 24739260:ui OR 24804802:ui OR 24805910:ui OR 24829866:ui OR 24834485:ui OR 24845177:ui OR 24856854:ui OR 24869977:ui OR 24870782:ui OR 24882370:ui OR 24891269:ui OR 24894145:ui OR 24894838:ui OR 24911404:ui OR 24916951:ui OR 24987274:ui OR 24997174:ui OR 25037667:ui OR 25060288:ui OR 25073541:ui OR 25088804:ui OR 25110844:ui OR 25124456:ui OR 25132146:ui OR 25148839:ui OR 25171662:ui OR 25179578:ui OR 25181937:ui OR 25186211:ui OR 25304986:ui OR 25313756:ui OR 25336068:ui OR 25337546:ui OR 25338320:ui OR 25338995:ui OR 25349070:ui OR 25367403:ui OR 25394834:ui OR 25417174:ui OR 25417947:ui OR 25427610:ui OR 25452437:ui OR 25526904:ui OR 25545411:ui OR 25555831:ui OR 25563360:ui OR 25567329:ui OR 25576214:ui OR 25602030:ui OR 25614924:ui OR 25645134:ui OR 25672583:ui OR 25681782:ui OR 25708515:ui OR 25711654:ui OR 25711667:ui OR 25724409:ui OR 25730596:ui OR 25739642:ui OR 25749595:ui OR 25749601:ui OR 25751587:ui OR 25752971:ui OR 25757733:ui OR 25781872:ui OR 25784579:ui OR 25792854:ui OR 25793000:ui OR 25814054:ui OR 25832894:ui OR 25833659:ui OR 25833781:ui OR 25845088:ui OR 25853030:ui OR 25899303:ui OR 25901376:ui OR 25903195:ui OR 25920528:ui OR 25935584:ui OR 25964257:ui OR 25965782:ui OR 25975457:ui OR 25975675:ui OR 25980790:ui OR 26000593:ui OR 26016834:ui OR 26026737:ui OR 26051073:ui OR 26059936:ui OR 26059937:ui OR 26068412:ui OR 26110777:ui OR 26175059:ui OR 26199085:ui OR 26202538:ui OR 26251693:ui OR 26282657:ui OR 26284121:ui OR 26286486:ui OR 26303657:ui OR 26304504:ui OR 26315617:ui OR 26327866:ui OR 26362355:ui OR 26373696:ui OR 26395825:ui OR 26427563:ui OR 26512712:ui OR 26518022:ui OR 26543382:ui OR 26543789:ui OR 26586495:ui OR 26593858:ui OR 26598750:ui OR 26602701:ui OR

26605003:ui OR 26606746:ui OR 26640827:ui OR 26655430:ui OR 26677207:ui OR
26679449:ui OR 26703650:ui OR 26706665:ui OR 26713501:ui OR 26714371:ui OR
26715294:ui OR 26756114:ui OR 26769117:ui OR 26777589:ui OR 26782031:ui OR
26801931:ui OR 26820653:ui OR 26829081:ui OR 26840439:ui OR 26850265:ui OR
26855719:ui OR 26869680:ui OR 26880252:ui OR 26887585:ui OR 26896305:ui OR
26915025:ui OR 26923090:ui OR 26925999:ui OR 26932848:ui OR 26935995:ui OR
26945570:ui OR 26960972:ui OR 26970957:ui OR 26973913:ui OR 26984227:ui OR
26988367:ui OR 27037811:ui OR 27061740:ui OR 27100859:ui OR 27110131:ui OR
27129840:ui OR 27146839:ui OR 27161493:ui OR 27187092:ui OR 27192633:ui OR
27197276:ui OR 27226430:ui OR 27232866:ui OR 27252076:ui OR 27254272:ui OR
27258052:ui OR 27293508:ui OR 27295811:ui OR 27312845:ui OR 27332968:ui OR
27333128:ui OR 27334210:ui OR 27368882:ui OR 27409075:ui OR 27416835:ui OR
27417791:ui OR 27433356:ui OR 27455846:ui OR 27464488:ui OR 27470258:ui OR
27490111:ui OR 27498099:ui OR 27516182:ui OR 27524376:ui OR 27529826:ui OR
27530961:ui OR 27531024:ui OR 27531738:ui OR 27539586:ui OR 27543047:ui OR
27543065:ui OR 27557405:ui OR 27562357:ui OR 27565830:ui OR 27601139:ui OR
27612561:ui OR 27629548:ui OR 27635602:ui OR 27646264:ui OR 27668296:ui OR
27718533:ui OR 27737976:ui OR 27738306:ui OR 27742668:ui OR 27752993:ui OR
27766453:ui OR 27785639:ui OR 27793125:ui OR 27821934:ui OR 27821958:ui OR
27832718:ui OR 27838922:ui OR 27840340:ui OR 27857248:ui OR 27857276:ui OR
27858198:ui OR 27863058:ui OR 27865259:ui OR 27868156:ui OR 27873046:ui OR
27881518:ui OR 27883168:ui OR 27884071:ui OR 27887610:ui OR 27893212:ui OR
27893938:ui OR 27909546:ui OR 27912947:ui OR 27925359:ui OR 27976933:ui OR
27981118:ui OR 27988868:ui OR 28012121:ui OR 28026901:ui OR 28070770:ui OR
28075038:ui OR 28084890:ui OR 28097626:ui OR 28150044:ui OR 28161788:ui OR
28166765:ui OR 28178355:ui OR 28189100:ui OR 28228661:ui OR 28242595:ui OR
28247126:ui OR 28253845:ui OR 28258052:ui OR 28288595:ui OR 28347291:ui OR
28392934:ui OR 28410162:ui OR 28410164:ui OR 28410171:ui OR 28410174:ui OR
28411330:ui OR 28414894:ui OR 28417203:ui OR 28427084:ui OR 28427925:ui OR
28444532:ui OR 28444534:ui OR 28470368:ui OR 28470507:ui OR 28489508:ui OR
28525464:ui OR 28538261:ui OR 28551083:ui OR 28553684:ui OR 28561685:ui OR
28588911:ui OR 28602712:ui OR 28620700:ui OR 28624715:ui OR 28624949:ui OR
28627254:ui OR 28632240:ui OR 28634625:ui OR 28636526:ui OR 28639097:ui OR
28639157:ui OR 28665541:ui OR 28666465:ui OR 28698390:ui OR 28702218:ui OR
28717378:ui OR 28721384:ui OR 28736768:ui OR 28739540:ui OR 28741202:ui OR
28745131:ui OR 28756991:ui OR 28766098:ui OR 28803675:ui OR 28836379:ui OR
28838886:ui OR 28845551:ui OR 28856470:ui OR 28881471:ui OR 28894513:ui OR
28900822:ui OR 28922074:ui OR 28926676:ui OR 28935620:ui OR 28965138:ui OR
28991039:ui OR 29026725:ui OR 29043460:ui OR 29057279:ui OR 29076388:ui OR
29082588:ui OR 29086523:ui OR 29097298:ui OR 29129704:ui OR 29141853:ui OR
29162619:ui OR 29168064:ui OR 29187408:ui OR 29188593:ui OR 29222705:ui OR
29243165:ui OR 29252103:ui OR 29259819:ui OR 29260391:ui OR 29273954:ui OR
29319387:ui OR 29325658:ui OR 29330081:ui OR 29344401:ui OR 29356607:ui OR
29388687:ui OR 29390904:ui OR 29395306:ui OR 29402761:ui OR 29404842:ui OR
29409128:ui OR 29409922:ui OR 29470705:ui OR 29470804:ui OR 29471477:ui OR
29474729:ui OR 29478430:ui OR 29498250:ui OR 29501739:ui OR 29511965:ui OR
29526243:ui OR 29553651:ui OR 29579014:ui OR 29587682:ui OR 29593021:ui OR
29617707:ui OR 29637645:ui OR 29644889:ui OR 29645327:ui OR 29654416:ui OR
29683152:ui OR 29688834:ui OR 29698983:ui OR 29740782:ui OR 29746264:ui OR
29770553:ui OR 29789774:ui OR 29847241:ui OR 29869823:ui OR 29880779:ui OR
29881639:ui OR 29932460:ui OR 29936464:ui OR 29943097:ui OR 29944706:ui OR
29952241:ui OR 29961039:ui OR 30021554:ui OR 30060250:ui OR 30062572:ui OR
30067063:ui OR 30071146:ui OR 30077574:ui OR 30077954:ui OR 30117760:ui OR
30128855:ui OR 30129907:ui OR 30138021:ui OR 30180192:ui OR 30202391:ui OR

	30229557:ui OR 30233121:ui OR 30244781:ui OR 30247961:ui OR 30256657:ui OR 30272836:ui OR 30312118:ui OR 30320304:ui OR 30339481:ui OR 30340503:ui OR 30361831:ui OR 30368741:ui OR 30376366:ui OR 30400784:ui OR 30402781:ui OR 30415752:ui OR 30445595:ui OR 30485297:ui OR 30488344:ui OR 30500981:ui OR 30539102:ui OR 30543479:ui OR 30554276:ui OR 30554923:ui OR 30562305:ui OR 30584782:ui OR 30609982:ui OR 30638093:ui OR 30658629:ui OR 30659130:ui OR 30663156:ui OR 30694978:ui OR 30723698:ui OR 30766696:ui OR 30767964:ui OR 30775929:ui OR 30783858:ui OR 30789349:ui OR 30794107:ui OR 30826763:ui OR 30840336:ui OR 30850143:ui OR 30862228:ui OR 30864301:ui OR 30874369:ui OR 30874927:ui OR 30912010:ui OR 30912011:ui OR 30917793:ui OR 30933317:ui OR 30972643:ui OR 30980431:ui OR 30982113:ui OR 31003402:ui OR 31012968:ui OR 31012970:ui OR 31014267:ui OR 31028212:ui OR 31033073:ui OR 31041685:ui OR 31041830:ui OR 31046885:ui OR 31076901:ui OR 31087419:ui OR 31099634:ui OR 31121916:ui OR 31144265:ui OR 31165653:ui OR 31168711:ui OR 31173341:ui OR 31187251:ui OR 31187411:ui OR 31206630:ui OR 31217021:ui OR 31227537:ui OR 31236699:ui OR 31237570:ui OR 31244536:ui OR 31264183:ui OR 31269917:ui OR 31272399:ui OR 31275735:ui OR 31290395:ui OR 31309977:ui OR 31319398:ui OR 31354000:ui OR 31367917:ui OR 31377976:ui OR 31389108:ui OR 31395070:ui OR 31415063:ui OR 31444192:ui OR 31454519:ui OR 31475306:ui OR 31520215:ui OR 31561728:ui OR 31600424:ui OR 31604709:ui OR 31605312:ui OR 31605514:ui OR 31617073:ui OR 31617564:ui OR 31642349:ui OR 31689771:ui OR 31694687:ui OR 31712954:ui OR 31745553:ui OR 31752830:ui OR 31759342:ui OR 31763746:ui OR 31773112:ui OR 31773148:ui OR 31780003:ui OR 31791246:ui OR 31804383:ui OR 31853701:ui OR 31860135:ui OR 31912361:ui OR 31933148:ui OR 31964669:ui OR 31970652:ui OR 31994181:ui OR 31996235:ui OR 31998651:ui OR 32009481:ui OR 32019093:ui OR 32020356:ui OR 32020706:ui OR 32031221:ui OR 32059728:ui OR 32071117:ui OR 32090192:ui OR 32103358:ui OR 32111767:ui OR 32112354:ui OR 32130627:ui OR 32147571:ui OR 32193692:ui OR 32232722:ui OR 32234652:ui OR 32240461:ui OR 32249355:ui OR 32298326:ui OR 32298407:ui OR 32304828:ui OR 32306100:ui OR 32307459:ui OR 32307828:ui OR 32314053:ui OR 32314110:ui OR 32326769:ui OR 32344683:ui OR 32348688:ui OR 32379234:ui OR 32384481:ui OR 32385847:ui OR 32395379:ui OR 32404725:ui OR 32406224:ui OR 32407010:ui OR 32414347:ui OR 32415386:ui OR 32415529:ui OR 32441691:ui OR 32443355:ui OR 32462950:ui OR 32510461:ui OR 32521652:ui OR 32535764:ui OR 32546796:ui OR 32558129:ui OR 32599641:ui OR 32605075:ui OR 32607599:ui OR 32616883:ui OR 32631302:ui OR 32664375:ui OR 32664946:ui OR 32673270:ui OR 32673271:ui OR 32678375:ui OR 32681303:ui OR 32694366:ui OR 32698957:ui OR 32699997:ui OR 32754489:ui OR 32763011:ui OR 32803384:ui OR 32808383:ui OR 32819304:ui OR 32859615:ui OR 32868938:ui OR 32875536:ui OR 32877271:ui OR 32883829:ui OR 32885221:ui OR 32886189:ui OR 32906761:ui OR 32924562:ui OR 32930924:ui OR 32940768:ui OR 32955446:ui OR 32960338:ui OR 33010183:ui OR 33025451:ui OR 33025453:ui OR 33036706:ui OR 33059741:ui OR 33064204:ui OR 33072603:ui OR 33076863:ui OR 33089371:ui OR 33105793:ui OR 33109503:ui OR 33123481:ui OR 33128705:ui OR 33163551:ui OR 33172957:ui OR 33178447:ui OR 33194622:ui OR 33212425:ui OR 33218558:ui OR 33225208:ui OR 33289669:ui OR 33292676:ui OR 33305350:ui OR 33312762:ui OR 33312937:ui OR 33396551:ui OR 33403402:ui OR 33423594:ui OR 33452652:ui OR 33467265:ui OR 33473331:ui OR 33475511:ui OR 33482396:ui OR 33491338:ui OR 33503496:ui OR 33539554:ui OR 33550878:ui OR 33564742:ui OR 33570477:ui OR 33588948:ui OR 33613314:ui OR 33618699:ui OR 33624172:ui OR 33677463:ui OR 33677781:ui OR 33689150:ui OR 33689553:ui OR 33709302:ui OR 33737299:ui OR 33752433:ui OR 33754246:ui OR 33786507:ui OR 33787643:ui OR 33801189:ui OR 33807611:ui OR 33811517:ui OR 33815828:ui OR 33823832:ui OR 33840972:ui OR 33847588:ui OR 33849087:ui OR 33883475:ui OR 33890857:ui OR 33896706:ui OR 33912450:ui OR 33929533:ui OR 33935799:ui OR

	33950477:ui OR 33954891:ui OR 33956212:ui OR 33961667:ui OR 33976224:ui OR 33977342:ui OR 33978132:ui OR 33983599:ui OR 33989021:ui OR 33992279:ui OR 34011028:ui OR 34049837:ui OR 34066752:ui OR 34089422:ui OR 34125072:ui OR 34130213:ui OR 34135964:ui OR 34146655:ui OR 34147107:ui OR 34160294:ui OR 34161602:ui OR 34189070:ui OR 34204528:ui OR 34207765:ui OR 34213086:ui OR 34225091:ui OR 34258817:ui OR 34274495:ui OR 34281126:ui OR 34290337:ui OR 34305108:ui OR 34311713:ui OR 34313858:ui OR 34314878:ui OR 34328623:ui OR 34344171:ui OR 34355298:ui OR 34357555:ui OR 34359567:ui OR 34365016:ui OR 34389552:ui OR 34413391:ui OR 34426142:ui OR 34427806:ui OR 34436552:ui OR 34444332:ui OR 34456250:ui OR 34461975:ui OR 34463835:ui OR 34465518:ui OR 34467791:ui OR 34480935:ui OR 34491527:ui OR 34498828:ui OR 34503198:ui OR 34524631:ui OR 34546611:ui OR 34554567:ui OR 34559724:ui OR 34567220:ui OR 34578787:ui OR 34578984:ui OR 34579142:ui OR 34586509:ui OR 34589720:ui OR 34590926:ui OR 34593453:ui OR 34598902:ui OR 34609311:ui OR 34638355:ui OR 34639353:ui OR 34649267:ui OR 34651554:ui OR 34664019:ui OR 34674806:ui OR 34677269:ui OR 34684403:ui OR 34709529:ui OR 34712520:ui OR 34721081:ui OR 34729835:ui OR 34733065:ui OR 34741653:ui OR 34777343:ui OR 34778552:ui OR 34789309:ui OR 34812521:ui OR 34816921:ui OR 34823494:ui OR 34836345:ui OR 34887491:ui OR 34898001:ui OR 34922621:ui OR 34989962:ui OR 34994945:ui OR 35012011:ui OR 35022884:ui OR 35025064:ui OR 35040076:ui OR 35045736:ui OR 35057525:ui OR 35087367:ui OR 35091272:ui OR 35107399:ui OR 35109799:ui OR 35110796:ui OR 35112212:ui OR 35130491:ui OR 35140880:ui OR 35156143:ui OR 35171707:ui OR 35182789:ui OR 35191900:ui OR 35200150:ui OR 35207294:ui OR 35244811:ui OR 35246219:ui OR 35253020:ui OR 35253682:ui OR 35257993:ui OR 35265005:ui OR 35277360:ui OR 35278203:ui OR 35289219:ui OR 35289926:ui OR 35314958:ui OR 35326679:ui OR 35328964:ui OR 35331230:ui OR 35342756:ui OR 35347550:ui OR 35363152:ui OR 35363502:ui OR 35366505:ui OR 35391574:ui OR 35398558:ui OR 35401377:ui OR 35416341:ui OR 35428285:ui OR 35431214:ui OR 35441995:ui OR 35455864:ui OR 35460441:ui OR 35471614:ui OR 35477489:ui OR 35477651:ui OR 35486425:ui OR 35488902:ui OR 35509876:ui OR 35533876:ui OR 35578196:ui OR 35619910:ui OR 35620805:ui OR 35639865:ui OR 35641019:ui OR 35681702:ui OR 35700977:ui OR 35712474:ui OR 35731554:ui OR 35733142:ui OR 35736016:ui OR 35738114:ui OR 35749052:ui OR 35749369:ui OR 35750628:ui OR 35756985:ui OR 35771607:ui OR 35775225:ui OR 35778782:ui OR 35788372:ui OR 35795051:ui OR 35829982:ui OR 35841719:ui OR 35853152:ui OR 35861215:ui OR 35881067:ui OR 35894167:ui OR 35895236:ui OR 35896295:ui OR 35902321:ui OR 35930212:ui OR 35940850:ui OR 35952956:ui OR 35954479:ui OR 35992855:ui OR 35993633:ui OR 35994321:ui OR 36004842:ui OR 36012025:ui OR 36066332:ui OR 36076044:ui OR 36084228:ui OR 36092248:ui OR 36110260:ui OR 36141372:ui OR 36158576:ui OR 36170550:ui OR 36201052:ui OR 36207046:ui OR 36212795:ui OR 36220629:ui OR 36231542:ui OR 36238756:ui OR 36254717:ui OR 36259288:ui OR 36263723:ui OR 36269525:ui OR 36301400:ui OR 36311982:ui OR 36314766:ui OR 36336249:ui OR 36342348:ui OR 36400655:ui OR 36401194:ui OR 36403344:ui OR 36407802:ui OR 36412916:ui OR 36441715:ui OR 36451297:ui OR 36453589:ui OR 36462139:ui OR 36468339:ui OR 36474321:ui OR 36479658:ui OR 36482346:ui OR 36512140:ui OR 36512157:ui OR 36517097:ui OR 36525089:ui OR 36526826:ui OR 36535489:ui OR 36539160:ui OR 36542098:ui OR 36542473:ui OR 36550258:ui OR 36568235:ui OR 36579554:ui OR 36600391:ui OR 36612340:ui OR 36618335:ui OR 36652087:ui OR 36658635:ui OR 36674436:ui OR 36683179:ui OR 36689989:ui OR 36692626:ui OR 36694356:ui OR 36695128:ui OR 36711885:ui OR 36715766:ui OR 36723801:ui OR 36736725:ui OR 36761969:ui OR 36776335:ui OR 36777230:ui OR 36790946:ui OR 36792620:ui OR 36800978:ui OR 36814313:ui OR 36826074:ui OR 36828705:ui OR 36836931:ui OR 36843066:ui OR 36847633:ui OR 36855214:ui OR

	36874708:ui OR 36890601:ui OR 36892780:ui OR 36894449:ui OR 36901594:ui OR 36904284:ui OR 36912039:ui OR 36920460:ui OR 36933050:ui)
22	#21 NOT (22926087:ui OR 23225743:ui OR 23352440:ui OR 23438359:ui OR 23494869:ui OR 23519566:ui OR 23576689:ui OR 23596106:ui OR 23647980:ui OR 23695926:ui OR 23775970:ui OR 23844919:ui OR 23957903:ui OR 24554388:ui OR 24753546:ui OR 24934783:ui OR 25144625:ui OR 25153791:ui OR 25537522:ui OR 25637285:ui OR 25668509:ui OR 25695466:ui OR 25957010:ui OR 26059937:ui OR 26252280:ui OR 26324364:ui OR 26343172:ui OR 26503386:ui OR 26651471:ui OR 26809289:ui OR 26882537:ui OR 27151593:ui OR 27387096:ui OR 27529756:ui OR 27832718:ui OR 27881009:ui OR 28094664:ui OR 28133022:ui OR 28154107:ui OR 28187725:ui OR 28322759:ui OR 28391397:ui OR 28453622:ui OR 28497387:ui OR 28642039:ui OR 28806753:ui OR 28929925:ui OR 29270829:ui OR 29523666:ui OR 29649913:ui OR 29864112:ui OR 29951713:ui OR 30056387:ui OR 30136876:ui OR 30272598:ui OR 30286723:ui OR 30312190:ui OR 30535101:ui OR 30652562:ui OR 30710242:ui OR 30783858:ui OR 30802224:ui OR 30919252:ui OR 30943543:ui OR 30950445:ui OR 30969906:ui OR 30972643:ui OR 31094843:ui OR 31120271:ui OR 31157323:ui OR 31267779:ui OR 31274335:ui OR 31330023:ui OR 31375907:ui OR 31524020:ui OR 31617590:ui OR 31672949:ui OR 31780003:ui OR 31805025:ui OR 31925532:ui OR 32020433:ui OR 32060166:ui OR 32172321:ui OR 32173766:ui OR 32202706:ui OR 32207884:ui OR 32239145:ui OR 32277008:ui OR 32371507:ui OR 32506338:ui OR 32554133:ui OR 32571351:ui OR 32575442:ui OR 32618207:ui OR 32642951:ui OR 32645252:ui OR 32658377:ui OR 32715730:ui OR 32926116:ui OR 32935450:ui OR 33029754:ui OR 33107982:ui OR 33185782:ui OR 33221667:ui OR 33243013:ui OR 33484565:ui OR 33507443:ui OR 33565036:ui OR 33721925:ui OR 33821320:ui OR 33883478:ui OR 33899978:ui OR 34018096:ui OR 34050402:ui OR 34242744:ui OR 34289212:ui OR 34315289:ui OR 34353286:ui OR 34380488:ui OR 34387728:ui OR 34452552:ui OR 34499632:ui OR 34620115:ui OR 34825858:ui OR 34840208:ui OR 34853914:ui OR 34895583:ui OR 34948517:ui OR 35064432:ui OR 35098347:ui)
23	#22 NOT (l2001078602 OR l2002136690 OR l2002394223 OR l2002709267 OR l2002711216 OR l2002738219 OR l2002777051 OR l2002842553 OR l2002879057 OR l2003183624 OR l2003382155 OR l2003439466 OR l2003479541 OR l2004048127 OR l2004213604 OR l2004229495 OR l2004454291 OR l2004465943 OR l2004474406 OR l2004501225 OR l2004581621 OR l2004711273 OR l2005131908 OR l2005153371 OR l2005164693 OR l2005290693 OR l2005473372 OR l2005519102 OR l2005519240 OR l2005697435 OR l2005843605 OR l2005852118 OR l2005893027 OR l2006733731 OR l2006919579 OR l2007019558 OR l2007027068 OR l2007081793 OR l2007460952 OR l2007973516 OR l2007973737 OR l2008387513 OR l2010039293 OR l2010303414 OR l2010489508 OR l2010621175 OR l2011025169 OR l2011070432 OR l2011391203 OR l2011670402 OR l2012331407 OR l2013210947 OR l2013309680 OR l2013337637 OR l2013378513 OR l2013408646 OR l2013444748 OR l2013558001 OR l2013891223 OR l2013928639 OR l2014072488 OR l2014306533 OR l2014343872 OR l2014774962 OR l2014799366 OR l2014873775 OR l2014904875 OR l2015008830 OR l2015207786 OR l2015237148 OR l2015800934 OR l2015943523 OR l2016158539 OR l2016556154 OR l2016581939 OR l2016908618 OR l2017015461 OR l2017492561 OR l368956618 OR l369116620 OR l369338009 OR l369338755 OR l369380088 OR l372232613 OR l372885445 OR l373482131 OR l373747305 OR l373883705 OR l52177351 OR l52346123 OR l52407873 OR l52498998 OR l52503157 OR l52555312 OR l52565658 OR l53015657 OR l600142265 OR l600224982 OR l600346372 OR l601148833 OR l602628656 OR l603289820 OR l603620183 OR l603646984 OR l604328654 OR l605114137 OR l605609389 OR l606179334 OR l607030060 OR l607061919 OR l607132520 OR l607342849 OR l607384595 OR l607924117 OR l608561063 OR l608886223 OR l611639801 OR l612752357 OR l613222581 OR l613228719 OR l613318391 OR l614133805 OR l614364570 OR l614400856 OR l614521889 OR l615221376 OR l615271273 OR l615715402 OR l616056465 OR l616142672 OR l616155069 OR

	l616745154 OR l616879941 OR l617188119 OR l617261272 OR l617727911 OR l618024921 OR l618332824 OR l619429847 OR l619905629 OR l620404486 OR l620586989 OR l620712028 OR l621313978 OR l622131813 OR l622188278 OR l622441017 OR l622789887 OR l622904891 OR l623794814 OR l624162880 OR l624411021 OR l624516729 OR l625229244 OR l626237321 OR l626330149 OR l626439506 OR l626730189 OR l627105521 OR l627105684 OR l627135496 OR l627212438 OR l627801557 OR l628279065 OR l628611564 OR l628754993 OR l628831742 OR l628959553 OR l629742090 OR l630324517 OR l630951125 OR l631056612 OR l631056884 OR l631261515 OR l631422502 OR l631481961 OR l631807844 OR l632912007 OR l632948309 OR l633137060 OR l633280368 OR l633413983 OR l634246668 OR l634716876 OR l635136064 OR l635274975 OR l636017419 OR l636108074)

Table 1d: CINAHL® search strategy

Provider/Interface	Ebsco
Database	CINAHL® Plus with Full Text
Date searched	June 25, 2020
Database update	June 25, 2020
Search developer(s)	Helena M. VonVille
Limit to English	Yes
Date Range	2013-2020
Publication Types	No limit by publication type
Search filter source	No search filter used

S1	(TI (physical activit* or exercise or jogging or walking or running or swimming or biking or bicycling or aerobics or (strength N3 training) or (resistance n3 training) or yoga or dance)) or (AB (physical activit* or exercise or jogging or walking or running or swimming or biking or bicycling or aerobics or (strength N3 training) or (resistance n3 training) or yoga or dance))
S2	(MH "Exercise+") OR (MH "Physical Fitness")
S3	(MH "Motor Activity")
S4	(MH "Physical Endurance+")
S5	S1 or S2 or S3 or S4
S6	(MH "Breast Neoplasms") OR (MH "Carcinoma, Ductal, Breast") OR (MH "Hereditary Breast and Ovarian Cancer Syndrome") OR TI ((breast n4 cancer*) OR (breast n4 neoplasm*)) OR AB ((breast n4 cancer*) OR (breast n4 neoplasm*))
S7	S5 and S6
S8	(MH "Survivors") OR (MH "Cancer Survivors")
S9	TI survivor* OR AB survivor*
S10	S8 OR S9
S11	(MH "Health Promotion") OR (MH "Health Education") OR (MH "Experimental Studies+") OR (MH "Evaluation Research") OR (MH "Comparative Studies") OR (MH "Behavioral Research")
S12	TI (((randomised n7 trial*) OR (randomized n7 trial*) OR (controlled n3 trial*) OR (clinical n2 trial*) OR ((single OR doubl* OR tripl* OR treb*) and (blind* OR mask*)))) OR AB (((randomised n7 trial*) OR (randomized n7 trial*) OR (controlled n3 trial*) OR (clinical n2 trial*) OR ((single OR doubl* OR tripl* OR treb*) and (blind* OR mask*))))
S13	TI (intervention* OR health promotion OR health education) OR AB (intervention* OR health promotion OR health education)
S14	S11 OR S12 OR S13
S15	S5 and S7 and S10 and S14
S16	S15 Limiters - Published Date: 20130101-20201231; English Language
