# Supplementary material for: Updated systematic review and meta-analysis: taking the next step in physical activity behavioral interventions for post-treatment breast cancer survivors
Source: Breast Cancer Res Treat. 2026 Jan 10;215(2):56. doi: 10.1007/s10549-025-07892-3 (PMC12790553; doi:10.1007/s10549-025-07892-3)
Supplement: Supplementary file 2 — Supplementary file2 (PDF 98 KB) [file 10549_2025_7892_MOESM2_ESM.pdf]

Online Resource 2: Other Data Elements Extracted

Article Title: Updated systematic review and meta-analysis: taking the next step in physical activity behavioral interventions for post-treatment breast cancer survivors

Journal name: Breast Cancer Research and Treatment

Authors: Brianna N Leitzelar[a,b] , Alana R. Willis[c], Sarah N. Price[b], Janet A. Tooze[c], Helena M. VonVille[d], Rachel Lintz[e], Shirley M. Bluethmann[b]

Affiliations:
[a] School of Kinesiology, University of Minnesota-Twin Cities, Minneapolis, MN, USA
[b] Department of Social Sciences and Health Policy, Wake Forest University School of Medicine, Winston-Salem, NC, USA
[c] Department of Biostatistics and Data Science, Wake Forest University School of Medicine, Winston-Salem, NC, USA
[d] Health Sciences Library System, University of Pittsburgh, Pittsburgh, PA, USA
[e] Department of Public Health Sciences, Penn State College of Medicine, Hershey, PA, USA

Corresponding Author:
Brianna N. Leitzelar
Email: leitz025@umn.edu

Category	Data Element
Participant Characteristics	Age (range, mean, median)
	Age-related eligibility criteria
	Race/ethnicity (counts, predominant)
	Years since diagnosis (range, mean, median)
	Years since treatment (range, mean, median)
	Stage at diagnosis
	Treatment received
	Geographic origin: City (US, major metropolitan area (1M people or more); Medium (US, medium sized metropolitan area (250K to 999,999 people); Small (US, small population (50K to 250K people); Rural (US, sparsely populated or rural setting (less than 50K); Mixed (US, a mix of the above options); Abroad (Outside the US)
	Income level (lowest, highest, mean, median)
	Baseline physical activity levels
Study Characteristics	Study type (Randomized Controlled Trial (RCT) or quasi-RCT)
	Sample size (total and by group)
	Number of treatment groups
	Equivalence testing at baseline (yes/no, differences identified)
	Type of control group (receives nothing, wait list or delayed treatment, contact control, usual care, attention placebo, exercise comparison)
Intervention Characteristics	Intervention features
	Directions to participants
	Intervention setting (clinic, community, home, mixed)
	Intervention duration (weeks)
	Number of sessions (total, exercise only, behavioral counseling only, both)
	Minutes per session
	Session frequency
	Use of behavioral theory (yes/no)
	PA guidelines included (yes/no)
