# Supplementary material for: Updated systematic review and meta-analysis: taking the next step in physical activity behavioral interventions for post-treatment breast cancer survivors
Source: Breast Cancer Res Treat. 2026 Jan 10;215(2):56. doi: 10.1007/s10549-025-07892-3 (PMC12790553; doi:10.1007/s10549-025-07892-3)
Supplement: Supplementary file 3 — Supplementary file3 (PDF 1147 KB) [file 10549_2025_7892_MOESM3_ESM.pdf]

Online Resource 3: Supplemental Figures

Article Title: Updated systematic review and meta-analysis: taking the next step in physical activity behavioral interventions for post-treatment breast cancer survivors

Journal name: Breast Cancer Research and Treatment

Authors: Brianna N Leitzelar[a,b], Alana R. Willis[c], Sarah N. Price[b], Janet A. Tooze[c], Helena M. VonVille[d], Rachel Lintz[e], Shirley M. Bluethmann[b]

Affiliations:

[a] School of Kinesiology, University of Minnesota-Twin Cities, Minneapolis, MN, USA

[b] Department of Social Sciences and Health Policy, Wake Forest University School of Medicine, Winston-Salem, NC, USA

[c] Department of Biostatistics and Data Science, Wake Forest University School of Medicine, Winston-Salem, NC, USA

[d] Health Sciences Library System, University of Pittsburgh, Pittsburgh, PA, USA

[e] Department of Public Health Sciences, Penn State College of Medicine, Hershey, PA, USA

Corresponding Author:

Brianna N. Leitzelar

Email: leitz025@umn.edu

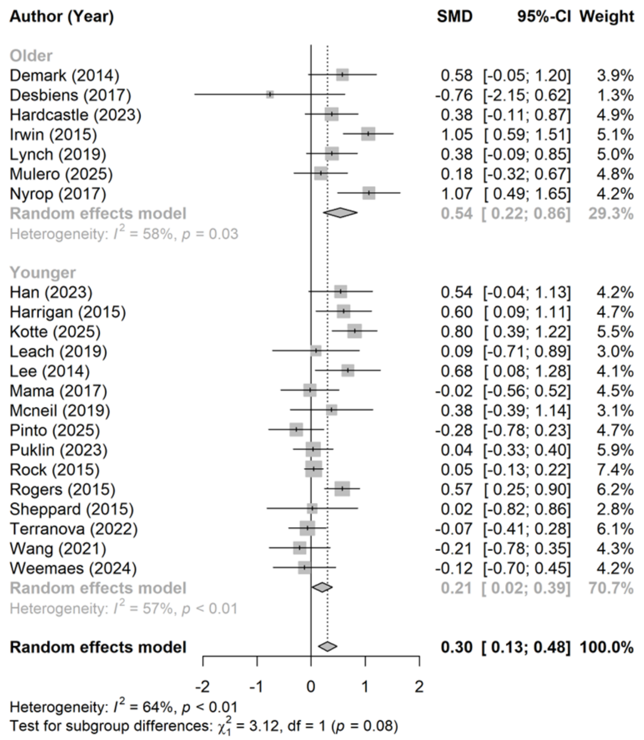

Figure OR3.1. Forest plot illustrating the subgroup effects of participant age group (older, younger) on the intervention's impact on physical activity outcomes. Studies were categorized using the average participant age weighted by the study sample size using a cut point of 60 years. Standardized mean differences (SMD; Cohen's d) with 95% confidence intervals (CIs) were calculated using the methods described by Wilson (2017) [24]. Data were pooled within each subgroup using a random-effects model, and subgroup differences were tested using a chi-squared test. Each grey box represents the SMD for an individual study, with horizontal lines indicating the corresponding 95% CI. Grey diamonds indicate the pooled effect and 95% CI for each subgroup and the overall effect. The vertical dotted line represents the overall pooled effect across all studies. Positive values reflect a favorable effect in the direction of the intervention group

Online Resource 3: Supplemental Figures

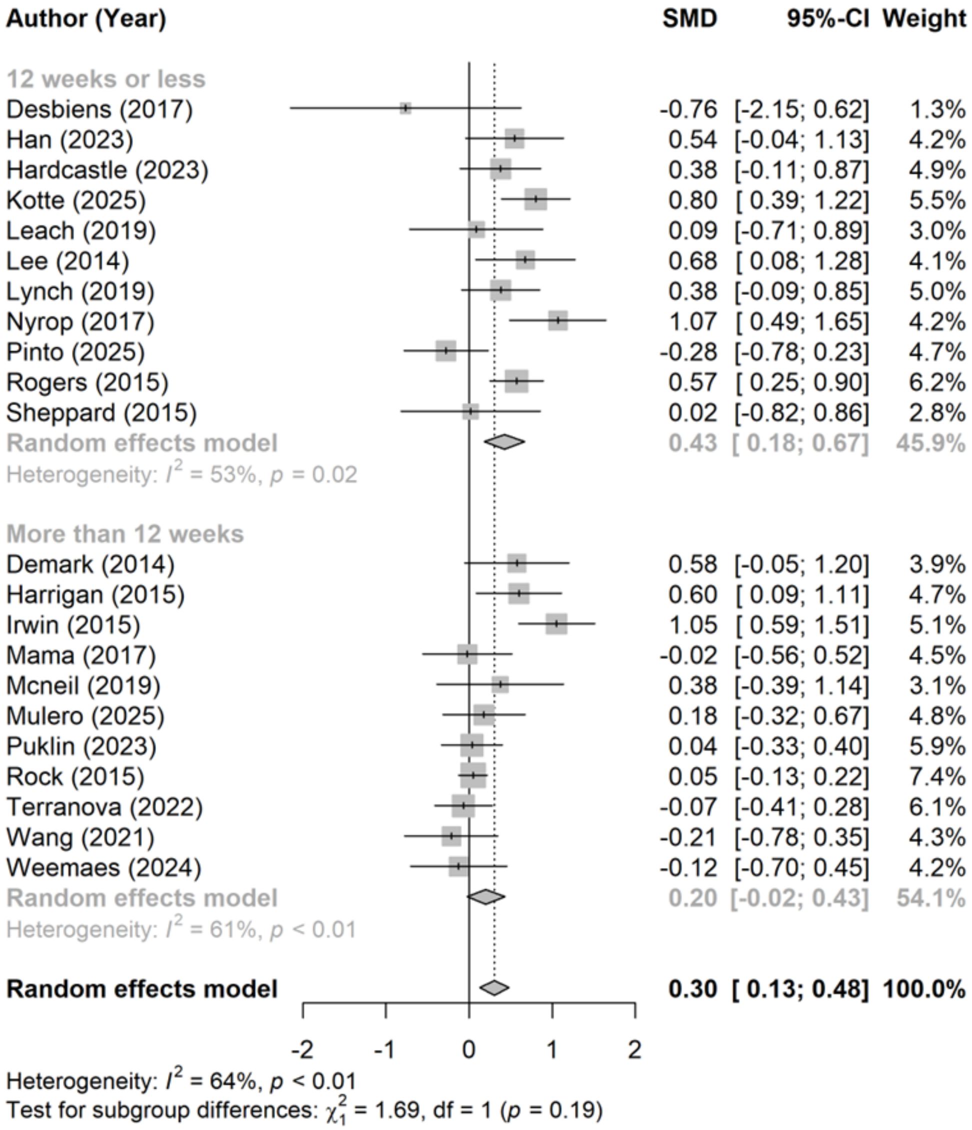

Figure OR3.2 Forest plot illustrating the subgroup effects of intervention duration (12 weeks or less, more than 12 weeks) on the intervention's impact on physical activity outcomes. Standardized mean differences (SMD; Cohen's d) with 95% confidence intervals (CIs) were calculated using the methods described by Wilson (2017). Data were pooled within each subgroup using a random-effects model, and subgroup differences were tested using a chi-squared test. Each grey box represents the SMD for an individual study, with horizontal lines indicating the corresponding 95% CI. Grey diamonds indicate the pooled effect and 95% CI for each subgroup and the overall effect. The vertical dotted line represents the overall pooled effect across all studies. Positive values reflect a favorable effect in the direction of the intervention group

Online Resource 3: Supplemental Figures

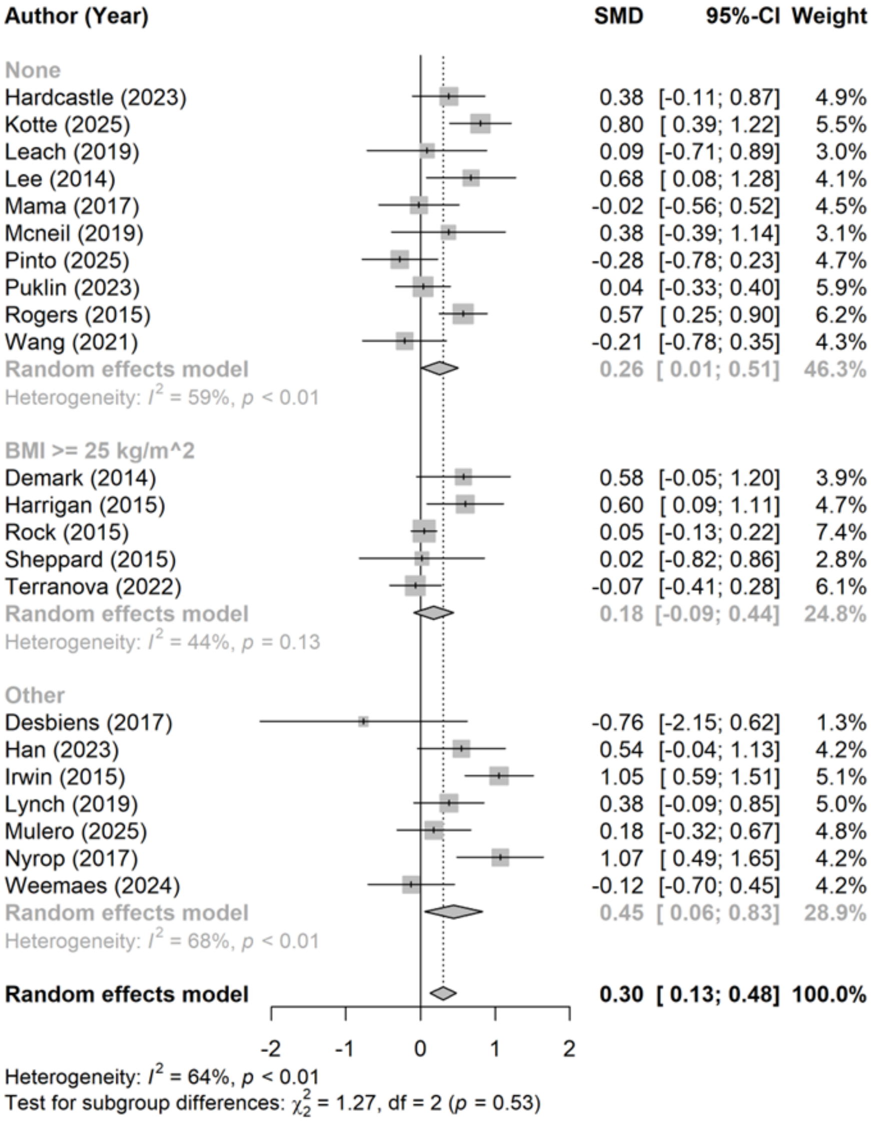

Figure OR3.3 Forest plot illustrating the subgroup effects of eligibility criteria on the intervention's impact on physical activity outcomes. All studies included breast cancer survivors (BCS) $\leq$5 years post-treatment, with some applying additional eligibility criteria. Studies were categorized as follows: None (no additional eligibility criteria), BMI of $\geq$ 25 kg/m^2 (additional criteria based on BMI), or other (post-menopausal, 50 years or older, taking AIs, experiencing symptoms such as pain or fatigue). Standardized mean differences (SMD; Cohen's d) with 95% confidence intervals (CIs) were calculated using the methods described by Wilson (2017). Data were pooled within each subgroup using a random-effects model, and subgroup differences were tested using a chi-squared test. Each grey box represents the SMD for an individual study, with horizontal lines indicating the corresponding 95% CI. Grey diamonds indicate the pooled effect and 95% CI for each subgroup and the overall effect. The vertical dotted line represents the overall pooled effect across all studies. Positive values reflect a favorable effect in the direction of the intervention group

BMI = Body mass index; AI = aromatase inhibitors

Online Resource 3: Supplemental Figures

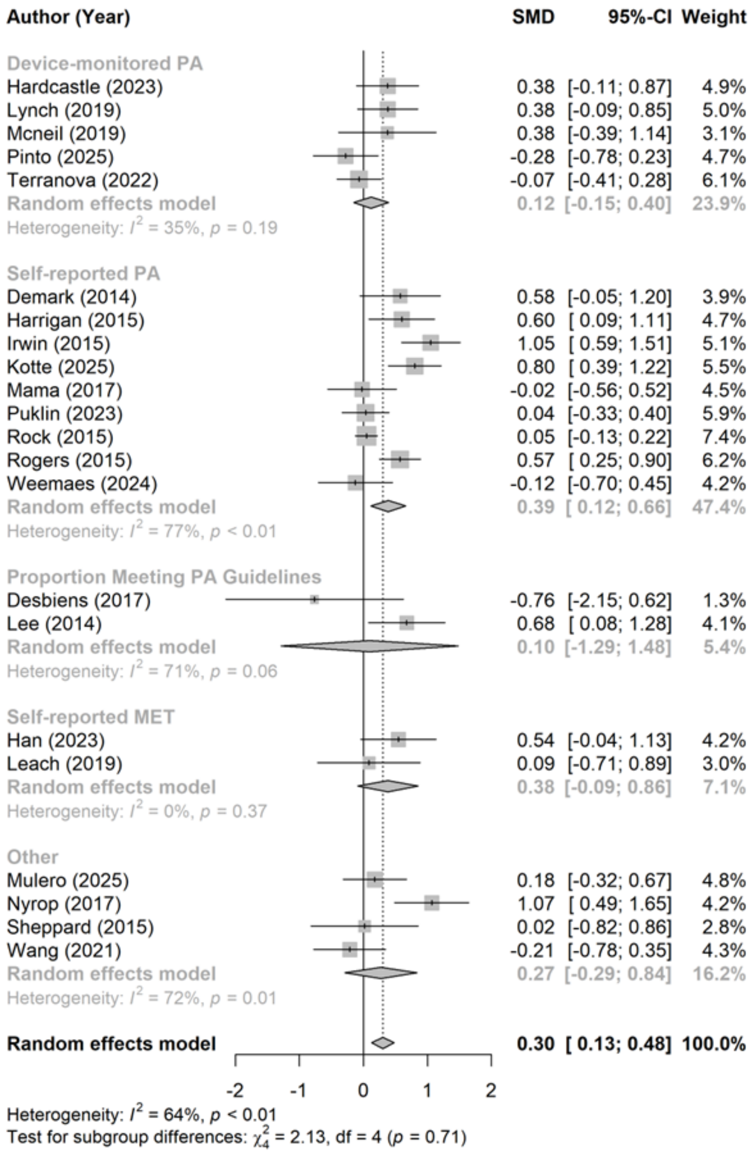

Figure OR3.4 Forest plot illustrating the subgroup effects of type of physical activity (PA) outcome assessed on the intervention's impact on PA outcomes. For each study, the outcome most closely aligned with moderate-to-vigorous physical activity (MVPA) was selected for analysis, though the specific measure varied. PA outcomes were categorized as follows: device-monitored PA (MVPA per week); self-reported PA (MVPA per week), Proportion Meeting PA Guidelines (activity classification based on proportion of participants meeting PA guidelines or categorized as insufficiently active, moderately active/active), self-reported MET (metabolic equivalent hours per week); and Other (walking minutes per week, total PA minutes per week, total leisure PA, or steps per day. Standardized mean differences (SMD; Cohen's d) with 95% confidence intervals (CIs) were calculated using the methods described by Wilson (2017). Data were pooled within each subgroup using a random-effects model, and subgroup differences were tested using a chi-squared test. Each grey box represents the SMD for an individual study, with horizontal lines indicating the corresponding 95% CI. Grey diamonds indicate the pooled effect and 95% CI for each subgroup and the overall effect. The vertical dotted line represents the overall pooled effect across all studies. Positive values reflect a favorable effect in the direction of the intervention group
